# Novel soil-inhabiting clades fill gaps in the fungal tree of life

**DOI:** 10.1186/s40168-017-0259-5

**Published:** 2017-04-08

**Authors:** Leho Tedersoo, Mohammad Bahram, Rasmus Puusepp, R. Henrik Nilsson, Timothy Y. James

**Affiliations:** 1 Natural History Museum, University of Tartu, 14a Ravila, 50411 Tartu, Estonia; 2 Institute of Ecology and Earth Sciences, University of Tartu, 14a Ravila, 50411 Tartu, Estonia; 3 Systematic Biology, Evolutionary Biology Centre, Uppsala University, Norbyvägen 18D, 75236 Uppsala, Sweden; 4 Department of Biological and Environmental Sciences, University of Gothenburg, Box 463, 405 30 Göteborg, Sweden; 5 Department of Ecology and Evolutionary Biology, University of Michigan, Ann Arbor, MI 48109 USA

**Keywords:** Phylogenetic lineages, Kingdom Fungi, Niche modelling, Random forest, Biogeography

## Abstract

**Background:**

Fungi are a diverse eukaryotic group of degraders, pathogens, and symbionts, with many lineages known only from DNA sequences in soil, sediments, air, and water.

**Results:**

We provide rough phylogenetic placement and principal niche analysis for >40 previously unrecognized fungal groups at the order and class level from global soil samples based on combined 18S (nSSU) and 28S (nLSU) rRNA gene sequences. Especially, Rozellomycota (Cryptomycota), Zygomycota *s.lat*, Ascomycota, and Basidiomycota are rich in novel fungal lineages, most of which exhibit distinct preferences for climate and soil pH.

**Conclusions:**

This study uncovers the great phylogenetic richness of previously unrecognized order- to phylum-level fungal lineages. Most of these rare groups are distributed in different ecosystems of the world but exhibit distinct ecological preferences for climate or soil pH. Across the fungal kingdom, tropical and non-tropical habitats are equally likely to harbor novel groups. We advocate that a combination of traditional and high-throughput sequencing methods enable efficient recovery and phylogenetic placement of such unknown taxonomic groups.

**Electronic supplementary material:**

The online version of this article (doi:10.1186/s40168-017-0259-5) contains supplementary material, which is available to authorized users.

## Background

Fungi are one of the key microbial groups in terrestrial ecosystems that enabled colonization of land by plants and facilitated development of soil that supports most of the biota on Earth [1, 2]. The kingdom Fungi is one of the most diverse groups of life with an estimated 1.5–6 million species that represent heterotrophic mutualists, pathogens, and saprotrophs [3, 4]. The 70,000–100,000 currently recognized species are distributed among 156 orders, 46 classes, and 12 phyla [3, 5, 6]. Fungi have traditionally been identified and classified based on morphological characters of fruiting bodies and living cultures. Similar to bacteria and archaea, merely <1% of fungal species have been cultivated with established protocols, which renders large taxonomic groups undescribed and virtually unknown to science [6, 7]. Roughly 80% of all soil-inhabiting fungal taxa cannot be identified at the species level, and 20% cannot be reliably assigned to known orders [8].

For the last two decades, molecular discovery and characterization of fungi have rapidly outpaced traditional morphological description. Public sequence databases have accumulated internal transcribed spacer (ITS) barcodes [9] representing hundreds of groups of closely related fungal species with no taxonomic identity due to the paucity of relevant reference sequences and lack of phylogenetically informative ribosomal RNA (rRNA) genes [10] (Additional file 1). Studies using a single molecular marker have shed light on several divergent but undescribed lineages of marine and terrestrial organisms among bacteria [11], protists [12], and fungi [13, 14]. Analysis of multiple genetic markers obtained from vegetative tissues, single-cell genomics, or whole metagenome assays of the environment has improved the phylogenetic placement and classification for many of these previously unknown organisms [14–17], but many more remain overlooked [10]. Because many of these lineages are not known from voucher material, the inability to name organisms only on the basis of sequence data hinders higher-level classification of fungi and other taxa [18].

**Fig. 1:**
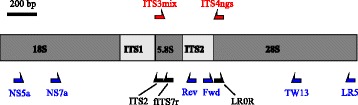
Primer map indicating the construction of long 18S-ITS-28S rRNA gene sequences from 454 pyrosequencing-based ITS2 amplicons (in *red*) and Sanger sequencing of flanking conservative genes using taxon-specific reverse (*Rev*) and forward (*Fwd*) primers in combination with eukaryote primers (in *blue*). Primer information is given in Additional file 2: Table S1

Here, we aim to determine the phylogenetic placement of previously unclassified soil fungi by developing 452 taxon-specific primers (Additional file 2: Table S1) targeting nuclear 18S (nSSU) and 28S (nLSU) rRNA genes in 263 ITS-based operational taxonomic units (OTUs) from global soil samples analyzed by Tedersoo et al. [8]. Since the long 18S-ITS-28S rRNA gene sequences were generated by combining several amplicons from Sanger sequencing and 454 pyrosequencing (Fig. 1), we performed a multi-step quality control to exclude any potentially artefactual entities. For the recovered novel soil fungal lineages, our purpose was to establish broad ecological niches for climatic and edaphic parameters and to determine geographic distribution together with endemicity patterns. We hypothesized that tropical soils harbor relatively more enigmatic fungal lineages, because (i) tropical habitats exhibit greater speciation but lower extinction rates [19], (ii) tropical forests harbor greater fungal richness [8], and (iii) lower latitudes are relatively poorly covered by biodiversity and taxonomic research [3].

## Results and discussion

### Novel clades of fungi

Phylogenetic analyses revealed 37 major clades and seven single branches (singleton lineages) of previously unrecognized or unclassified fungi with distinct phylogenetic position that warrant at least order-level classification (Additional file 1: Text S1). In the 18S rRNA gene and concatenated gene analyses, the clade GS01 was placed in a sister position to all remaining fungi, although the statistical support for this and most other early branching configurations remained poor (Additional file 1: Figures S1-S3).

Altogether, 11 clades (GS2–GS12) and three distinct branches (32%) of previously unclassified soil fungi were placed within Rozellomycota (Cryptomycota). Our findings highlight that the remarkable phylogenetic diversity of Rozellomycota from aquatic ecosystems [14, 20] is also observed in terrestrial habitats. Unlike in recent analyses [21], Rozellomycota was separated from the phylum Aphelidea that accommodates the clade GS16, a large and well-supported group with no taxonomically characterized representatives. Other zoosporic phyla accommodated fewer undescribed fungal clades. Chytridiomycota harbored two distinct environmental groups, the clade GS13 with an unsettled position, and the clade GS14 in a sister position to Spizellomycetales. The clade GS15 formed a long branch within the Blastocladiomycota, albeit with low support (BS <70). Two clades of closely related soil fungi clustered with the enigmatic “chytrid” genus *Olpidium* that warrants a (sub)phylum of its own [22]. Taxonomically uncharacterized novel lineages of Chytridiomycota s.lat. are particularly common in freshwater [23] and marine environments [24].

Among the former zygomycetes, the clade GS19 formed a deep lineage at the base of Kickxellomycotina and Zoopagomycotina. Clades GS20, GS21, and GS22 were loosely associated with Endogonales (Mucoromycotina), whereas a single group (clade GS23) formed a monophyletic branch with Umbelopsidaceae (Mucoromycotina). All these groups warrant at least class-level distinction from other mucoralean taxa [25]. A single novel clade of Glomeromycota—clade GS24—displayed strong affinities to Paraglomerales. From this group, a single spore collection (INSD accession JN936327) has been sequenced but not yet described.

Three class-level clades were related to the subphylum Pucciniomycotina of the Basidiomycota. Clades GS25 and GS26 represented successive sister groups to the remaining Pucciniomycotina, whereas the clade GS27 formed a sister group to Agaricostilbomycetes. The latter clade includes an 18S rRNA gene (Sanger) sequence from the voucher specimen RB1040 named as *Platygloea* sp. that appears distantly related to other Platygloeales and other Pucciniomycotina. Three novel clades (GS28–GS30) and branches were identified within the early-diverging Agaricomycetes, but their sister groups remained poorly resolved (BS <70). Multiple divergent sequences were also recovered in the orders Sebacinales, Trechisporales, Agaricales, Thelephorales, Hymenochaetales, and Atheliales.

Within Ascomycota, the Taphrinomycotina subphylum included a well-supported sister group (clade GS31) to the Archaeorhizomycetes, a recently described class that is largely composed of environmental sequences [16]. The clades GS32 and GS33 were closely related to the Orbiliales within Orbiliomycetes. Several additional unidentified taxa clustered within Pezizomycetes, but no deep lineages were evident in this group. Phylogenetic relationships of other classes of the Pezizomycotina were more poorly resolved, but these comprised four previously unidentified order-level clades (GS34–GS37) and two prominent branches as well as multiple taxa with clear affinities to known orders. These clades were related to the Eurotiomycetes, Lecanoromycetes, Sordariomycetes, or Symbiotaphrinales, albeit with no support. In contrast to multiple novel lineages in the early diverging fungal phyla, no such deep undescribed lineages of Dikarya were evident from aquatic environments [24].

### Distribution of previously unrecognized clades

Niche modelling of the clades and prominent branches revealed that the distribution of most groups is significantly related to climatic or edaphic conditions. Across the 41 most common groups, the mean annual temperature (MAT), mean annual precipitation (MAP), time since last fire, and soil pH accounted for the strongest predictors in 44, 20, 15, and 12% of the taxa, respectively (Fig. 3, Additional file 1: Figures S4-S8). Soil C concentration and soil P concentration had a predominant effect in only a few cases (Additional file 1: Figures S4, S8). Altogether 46% of the groups had a preference for tropical climate as judged by their distribution patterns relative to MAT and MAP (Additional file 1: Figures S5, S6; Text S1). In contrast, 32% of the groups were distinctly more frequent in cool temperate climate, whereas 7 and 5% of the groups peaked in warm temperate soils and tundra soils, respectively.

While 39% of the groups had a unimodal relationship with pH, peaking at moderately acidic values, some 32 and 7% of the groups exhibited preference for highly acidic and neutral soil, respectively (Additional file 1: Figure S7; Text S1). In terms of soil pH and climate, similar preference patterns were described for the most species-rich classes of fungi [8]. The more common niche development in acidic soils relative to neutral soils may be related to the characteristic substrate of saprotrophic fungi in strongly or moderately acidic humus derived from litter. It is also possible that less intense sampling in neutral soils may have rendered selection of the rare alkaliphilous groups less likely and that it may have favored non-selective groups instead.

Several groups of Rozellomycota exhibited preference for either of the extreme pH conditions, although the whole phylum taken together did not respond to soil pH. Except for the clades GS10 and GS11, all divergent groups of Rozellomycota were relatively more common in cool temperate or subarctic climate, which stands in stark contrast to the suggested niche of early diverging fungal lineages in tropical latitudes [26]. Frequent clade formation of the Rozellomycota isolates from soil with those from freshwater, marine, and anoxic habitats suggests that specialization for physical habitat is relatively limited, but distribution of these groups may be influenced by substrate pH at the clade level. It is also possible that the definition of the Rozellomycota clades is too broad for detecting environmental patterns, because their age may exceed that of relatively more recently evolved phyla in Dikarya [27]. As all known members of Rozellomycota (incl. Microsporidia) and Aphelidea are obligate pathogens of various other eukaryotes, such as amoebae, algae, and other fungi [20], the distribution of these species may depend indirectly on interaction specificity and habitat preference of host organisms.

In contrast to Rozellomycota, the undescribed ascomycete clades were generally more prominent in warm and moist tropical climates, and their relative abundance peaked in moderately acidic soils. The most common ascomycete classes varied greatly in their preference for climate and pH [8]. These group-specific responses and the presence of multiple functional groups caution against phylum-level analyses of fungal ecological patterns [28].

**Fig. 2:**
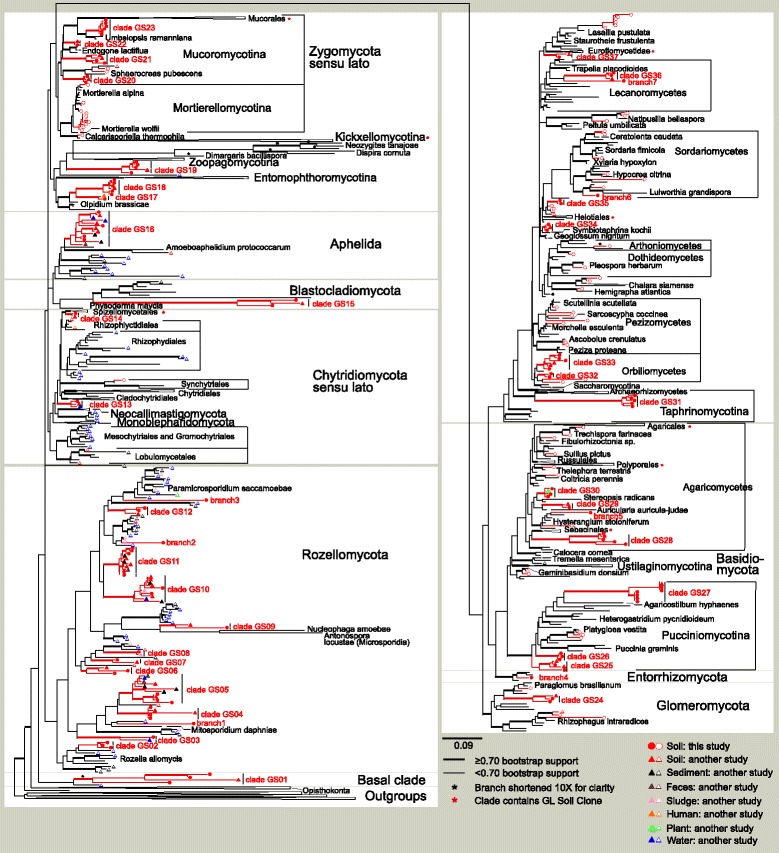
Phylogenetic placement of soil-inhabiting fungi among identified taxa and other sequences from the environment based on a simplified concatenated 18S and 28S rRNA gene maximum likelihood phylogram. Several branches were collapsed for clarity. *Circles* and *triangles* denote sequences from the present and other studies, respectively. *Closed symbols* indicate sequences belonging to the 44 defined clades and prominent branches; *open symbols* indicate sequences belonging to other groups. *Terminal taxa without symbols* represent identified collections, of which the most critical ones are shown for clarity

Most of the undescribed clades and branches were rare but nonetheless widely distributed in different habitats. The niche analysis revealed that roughly half of the groups had significant differences in geographic distribution among biomes and regions (Table 1). In particular, Europe, Central America, and Southern South America stood out as focal geographic regions for a large proportion of the undescribed groups. The groups branch5 (four OTUs), clades GS06 (five OTUs), and GS26 (four OTUs) exhibited the strongest endemicity, being distributed exclusively in Australia, Europe, and Northern South America, respectively. These extreme patterns are at least partly attributable to geographically aggregated and insufficient taxonomic sampling of the uncommon groups. For many other undescribed clades, the complementary information in sequence databases provides ample evidence for more widespread distribution in soil and furthermore suggests that several clades of the early-diverging fungal phyla may actually be relatively more common in aquatic environments (Fig. 2).

**Table 1:** Niche analysis of clades and branches of undescribed fungi

Group	Representative: accession; OTU; sample	No sequences; occurrences; OTUs	Niche and habitat
Clade GS01, unassigned phylum	UDB014611; GL00251; S114	230; 52; 26	Low MAT; Europe, Southern South America
Clade GS02, Rozellomycota	UDB014756; GL09833; G2846	78; 19; 6	Low MAP, near-neutral soils; Europe, temperate dec. forest
Clade GS03, Rozellomycota	UDB014679; GL04110; S136	31; 14; 9	Tolerates recent fire, low MAT; tundra
Branch1, Rozellomycota	UDB014728; GL07679; S234	16; 8; 4	Tolerates recent fire, high soil C; Central America
Clade GS04, Rozellomycota	UDB014664; GL03020; G2840	12; 7; 4	Intolerant of recent fire, low pH*; tundra
Clade GS05, Rozellomycota	UDB014721; GL06927; S132	276; 144; 63	Avoids recent fire; low MAT
Clade GS06, Rozellomycota	UDB014815; GL19521; G2819	37; 15; 5	Very low MAT** and MAP***; tundra and boreal forest, Europe
Clade GS07, Rozellomycota	UDB014956; GL50970; G2794	4; 4; 1	nd
Clade GS08, Rozellomycota	UDB014958; GL51158; G2819	7; 7; 2	Low MAT*** and MAP**; cool temperate forests
Clade GS09, Rozellomycota	UDB014949; GL48063; S131	8; 6; 3	Tolerates recent fire, low MAT***; cool temperate habitats
Clade GS10, Rozellomycota	UDB014882; GL31339; S084	189; 26; 10	High MAP, low pH; India
Clade GS11, Rozellomycota	UDB014836; GL23025; s206	2509; 716; 219	Low soil pH; moist tropical and temperate dec. forest
Branch2, Rozellomycota	UDB014923; GL39891; G2732	4; 2; 1	nd
Clade GS12, Rozellomycota	UDB014881; GL30957; G2839	31; 16; 8	Very low MAT; tundra and boreal forest
Branch3, Rozellomycota	UDB014895; GL33834; G2677	3; 2; 1	nd
Clade GS13, Chytridiomycota	UDB014650; GL02368; G2750	29; 10; 6	Very high MAT***; Australia; tropical dry forest
Clade GS14, Chytridiomycota	UDB014658; GL02816; S002	77; 12; 7	Warm temperate and tropical climate; Gondwanan
Clade GS15, Chytridiomycota	UDB014729; GL08046; S188	37; 26; 15	Moderately low pH; Southern South America
Clade GS16, Aphelida	UDB014619; GL00457; S238	25; 16; 7	Moderately low soil P; warm temperate climate
Clade GS17, Zygomycota *s.lat.*	UDB014847; GL23867; s124	57; 17; 3	Low MAP*** and MAT***, moderately low pH; Laurasian
Clade GS18, Zygomycota *s.lat.*	UDB014671; GL03481; G2835	162; 55; 14	Temperate climate, low pH; Eurasia
Clade GS19, Zygomycota *s.lat.*	UDB014747; GL09098; S008	312; 116; 75	Humid tropical climate, low pH; SE Asia
Clade GS20, Zygomycota *s.lat.*	UDB014697; GL04809; G2660	2364; 289; 36	High MAT, low pH; tropical rain forest, savannas
Clade GS21, Zygomycota *s.lat.*	UDB014852; GL24622; S049	14; 6; 6	High MAT*** and MAP***, low pH*
Clade GS22, Zygomycota *s.lat.*	UDB014740; GL08312; S171	52; 37; 11	Moderate MAT, very low pH; New Zealand
Clade GS23, Zygomycota *s.lat.*	UDB014792; GL15602; G2643	438; 80; 22	Very low pH; tropical rain forest
Clade GS24, Glomeromycota	UDB014833; GL22083; S045	38; 20; 16	Neutral pH; tropical climate
Branch4, Entorrhizomycota	UDB014934; GL42909; G2745	10; 6; 3	Tropical savannas
Clade GS25, Basidiomycota	UDB014764; GL10954; S159	63; 10; 2	Warm temperate climate
Clade GS26, Basidiomycota	UDB014713; GL06120; S060	161; 14; 4	High MAP*** and MAT**, very low pH***; Northern South America
Clade GS27, Basidiomycota	UDB014864; GL26681; S114	159; 102; 18	Low MAT; boreal and temperate deciduous forest
Clade GS28, Basidiomycota	UDB014693; GL04630; S004	187; 39; 14	High MAT*** and MAP***, very low pH; tropical moist forest
Branch5, Basidiomycota	UDB014858; GL26492; G2647	12; 5; 4	Prefers recent fire, high MAT*** and MAP**; Australia
Clade GS29, Basidiomycota	UDB014802; GL16303; AV123	140; 5; 3	Very high MAP** and MAT
Clade GS30, Basidiomycota	UDB014766; GL11329; G2641	212; 43; 12	High soil P, moderate MAT***; Gondwanan
Clade GS31, Ascomycota	UDB014859; GL26545; S046	341; 36; 18	Tropical climate, moderate pH; Central America
Clade GS32, Ascomycota	UDB014870; GL29325; G2660	18; 6; 3	High MAT*** and MAP***; Central America
Clade GS33, Ascomycota	UDB014886; GL32399; S049	80; 32; 21	Moderate MAP; Australia, tropical savannas
Clade GS34, Ascomycota	UDB014912; GL45481; G2629	43; 26; 14	Warm temperate climate
Clade GS35, Ascomycota	UDB014945; GL45252; S163	989; 177; 60	Tropical climate; Central America and Africa, grasslands
Branch6, Ascomycota	UDB014790; GL15471; G2658	113; 38; 18	Very high MAT, neutral soil pH; tropical dry forest
Branch7, Ascomycota	UDB014800; GL16288; AV103	115; 71; 20	Very high MAT*** and MAP***, very low pH***; Northern South America
Clade GS36, Ascomycota	UDB014939; GL43498; G2736	92; 37; 20	High MAT; montane rain forest
Clade GS37, Ascomycota	UDB014659; GL02919; S123	40; 15; 4	Moderate MAT*** and soil pH***; Southern South America

The groups are arranged by increasing distance from the fungal root. Asterisks indicate a significantly more narrow distribution compared with the null distribution (***P < 0.001; **P < 0.01; *P < 0.05)

*nd* not determined

### Implications of cryptic microbial diversity

Our study highlights the presence of multiple previously undescribed fungal groups and approximates their phylogenetic position within fungi. These clades and branches seem to represent only a tip of the iceberg in the ocean of unknown fungal lineages, because the groups recovered here matched at >80% similarity to only 13 out of >1000 compound clusters of ITS sequences with no order-level described representatives [10, 29] and we focused solely on a prominent but still limited subset of soil-inhabiting taxa. Contrary to our hypothesis of higher diversity of novel clades in the tropics, the preferred niche of undescribed groups was equally likely to be tropical or non-tropical. It is notable that nearly one third of these clades were also recovered from soil in a single comprehensively sampled field experiment in NC, USA [30], suggesting that numerous undescribed and widespread fungal lineages await discovery and formal description in single habitats. Most importantly, all fungal phyla accommodate previously unrecognized fungal groups, but Rozellomycota stands out as particularly understudied phylogenetically and taxonomically both in aquatic habitats [20, 24] and in soil. The great phylogenetic richness of Rozellomycota is probably related to their ecologically successful obligate energy parasitism on protists, fungi, and algae and a more recent switch (Microsporidia) to an intracellular habitat in animals. This may have resulted in their early radiation and accelerated evolution of various genes as well as overall genome compaction [20, 31].

DNA barcoding of culture collections and fungaria, as well as release of sequence data for public use, will certainly uncover true vouchered representatives of several of our undescribed clades and facilitate formal taxonomic description of these groups. Both fruiting bodies and cultures form an excellent basis for genomic analysis to understand the functional capacities of undescribed taxa and improve phylogenetic resolution [16, 32, 33]. Metagenomics and single-cell genomic analyses offer promising tools for taxonomic and functional characterization of bacteria [17] and aquatic microeukaryotes [34] in their intimate environment, and these methods may provide satisfactory results also for unicellular zoosporic fungi [20]. They nevertheless remain a major challenge in the context of multicellular fungi and other eukaryotes due to the typical growth of these taxa inside substrates, the 10–100 times greater genome size compared to bacteria, and the arrangement of genetic information in multiple chromosomes [35]. We predict that the combination of targeted DNA capture and sequencing of long metagenomics fragments will soon provide unprecedented insights into the phylogeny and function of eukaryotic microorganisms and shed light on tens to hundreds of previously unrecognized lineages of life.

We nevertheless fear that a non-trivial proportion of our undescribed lineages will cede little ground to immediate scientific scrutiny. The combination of uncultivability and not forming appreciable fruiting bodies or other tangible morphological structures is particularly problematic from a genomics point of view. Indeed, that very combination precludes both straightforward genome sequencing and formal description of the underlying species [18]. It will presumably take a long time before all the taxa presented here will have formal names. We hope that the scientific community is prepared to address these lineages using informal names, such as “clade GS01” (Additional file 1: Text S1), in the meanwhile. These taxa are every bit as real and worthy of scientific study as taxa bearing formal Latin classifications. The ecological roles and functional capacities of these undescribed lineages remain poorly understood, which makes their exploration all the more pressing given that fungi including the early diverging lineages represent important sources for pharmacy and the enzyme industry [36]. There is, furthermore, little reason to think that soil is the sole source for previously undescribed fungal lineages; it is likely that habitats and substrates such as water, sediments, and other organisms will prove to be equally rich sources of taxonomic dark matter [37, 38].

## Conclusions

This study extends and illustrates previous findings that the soil habitat harbors thousands of undescribed fungal taxa [8, 10, 13, 14], which we place to >30 previously unrecognized well-supported fungal lineages. More importantly, these order- and class-level groups are distributed throughout the fungal tree of life and exhibit specific ecological preferences and/or biogeographic distribution patterns. To enable cross-communication of these major phylogenetic clades among research groups, we propose a provisional naming system until their valid taxonomic description or matching with hitherto unsequenced species. These clade names are linked to fungal ITS and rRNA gene sequences in the UNITE database. Combining fluorescent probing and single-cell sequencing to cover nearly full-length rRNA genes will certainly improve our understanding about the ecophysiology and evolution of these enigmatic fungal clades.

## Methods

### Data generation

We used the global soil DNA samples and fungal ITS2 data set from 365 localities in 38 countries [8] to address phylogenetic and ecological hypotheses about the distribution of previously unknown fungal lineages. In brief, 40 subsamples of soil (50-mm diam. to 50-mm depth) were collected from each 2500-m^2^ site, pooled, air-dried, and pulverized. The soil powder was subjected to chemical analysis of macro- and micronutrients and DNA extraction (2 g) and 454 pyrosequencing, followed by quality filtering, clustering at 98% sequence similarity, and removal of singletons [8]. From the final data set of 50,589 operational taxonomic units (OTUs), we identified taxa originally assigned to fungi or rare protist groups as well as taxa with unknown taxonomic affiliations that displayed sequence similarity <80% to any species with a Latin binomial using BLASTn queries against an annotated copy of the International Nucleotide Sequence Databases (INSDc) as maintained in UNITE [39]. Depending on taxa, 80% ITS sequence similarity roughly corresponds to the family or order in fungi [8, 9]. Nearly 15% of all OTUs corresponded to this criterion, suggesting the presence of numerous new taxa at the family level or higher. Representative sequences of these OTUs were further clustered at 80% sequence similarity using single-linkage clustering and at least a 100-base coverage in Sequencher 5.1 (GeneCodes Corp., Ann Arbor, MI, USA) to assign individual OTUs to larger taxonomic groups. To ensure that all major taxonomic clusters (>10 OTUs) were covered, we selected 203 individual OTUs and 23 groups of closely related OTUs (altogether comprising 60 OTUs with sequence similarity >95% within groups) for design of taxon-specific primers and more detailed phylogenetic analyses. At 80% similarity level, the selected OTUs represented 1111 OTUs and 15,515 sequences. We sought to amplify the 3′ part of the 18S rRNA gene and the 5′ part of the 28S rRNA genes to allow phylogenetic inference at the kingdom level. For each of these taxa, we designed reverse and forward primers in the variable part of the ITS region according to the following criteria: (i) melting temperature of primers 54–58 °C; (ii) AT/CG ratio 33–62%; (iii) primer length 16–21 bases; (iv) perfect match of the last 10 bases to <20 OTUs in the whole data set (usually matching no other OTUs); and (v) distance from the flanking 5.8S and 28S rRNA genes >20 bases to allow detection of unspecific amplification.

To amplify the 18S rRNA gene, the specific reverse primers were paired with the NS5a and NS7a primers (Additional file 2: Table S1). To amplify the 28S rRNA gene, we combined the specific forward primers with TW13 and LR5. PCR with specific primers was performed for both of the two rRNA gene regions and two alternative primer combinations for 443 samples representing 263 OTUs. Sanger sequencing was performed bidirectionally using the universal PCR primers and the primers ITS2 and/or fITS7R for 18S rRNA gene or LR0R for 28S rRNA gene (Additional file 2: Table S1). Contigs were assembled in Sequencher with manual quality trimming. The reads obtained using 18S and 28S rRNA gene primers typically overlapped at least partly with the pyrosequenced ITS2 fragment, which allowed us to exercise initial chimera control. Individual sequences were further BLASTn-queried against GenBank to detect inconsistencies in the identification of 18S rRNA gene, ITS1, ITS2, and 28S rRNA gene sequences. Full-length sequences were also subjected to chimera detection using UCHIME [40] against other taxa in the data set and all INSDc entries spanning from 18S to 28S rRNA genes. These analyses revealed five potentially chimeric constructs that were removed. PCR and Sanger sequencing were successful for 244 samples of 18S (168 OTUs) and 298 samples of 28S (193 OTUs) rRNA genes. Altogether, 138 OTUs were represented by both 18S and 28S rRNA gene sequences, whereas sequencing failed completely for 25 OTUs. The most common issues with specific primers included (i) multiple amplicons seen as smear on the gel (18S rRNA gene), no amplification (18S and 28S rRNA genes), and poor fitting of the complementary sequencing primer, resulting in poor signal (18S rRNA gene). Individual reads were generally of high quality, indicating the sequence origin to be that of a single organism.

We obtained high-quality 18S and/or 28S rRNA gene Sanger sequences for 90.5% of the targeted OTUs, including all but two major groups (>10 OTUs). High-quality sequences were mainly recovered from samples with relatively high abundance of target DNA (>0.2% of ITS sequences), but in many cases, 18S and 28S rRNA gene data could be recovered from singletons, i.e., taxa contributing to <0.05% of all sequences per sample. Certain samples and OTUs failed to yield any amplicons, suggesting DNA degradation and unsuitability of the designed or eukaryote primers, respectively.

### Phylogenetic analyses

For phylogenetic inference, we used (i) the core 18S + 28S rRNA gene data set of James et al. [15] supplemented with (ii) 18S and 28S rRNA gene sequences of more recently obtained specimens or cultures of early diverging fungal lineages, (iii) 18S and 28S rRNA gene sequences of at least one representative of all fungal orders (except ascomycetes, for which representatives of ca. 70% orders and all classes were included), and 18S or 28S rRNA gene sequences of the best BLASTn hits (at least 600 bases) of our OTUs. Whenever possible, we included 18S and 28S rRNA gene sequences from the same specimen and preferably from the type species of that taxon for taxonomic reliability. Since we included best-matching sequences, the 18S and 28S rRNA gene data sets were unbalanced, comprising ca. 25% of non-overlapping entries. Initially, the two data sets were aligned separately in MAFFT 7 [41] with the FFT-NS-i option. Poorly aligned regions were removed using GBlocks v. 0.91b [42], with the following parameters: minimum number of sequences for a conserved position = 50% of sequences, minimum number of sequences for a flank position = 75% of sequences, minimum number of contiguous non-conserved positions = 20, minimum length of a block = 2, and allowed gap positions = All. The final alignment length of 18S and 28S rRNA genes was 1701 and 879 positions, respectively. Because the phylogenetic positions of target taxa were similar relative to the core specimens, we concatenated the two alignments for a joint analysis in addition to separate analyses. Phylograms were inferred using maximum likelihood as implemented in RAxML 7.2.8 using the GTRCAT model [43]. For the combined data set, 1000 heuristic searches were performed using a skeleton constraint tree for taxa in James et al. [15] and support estimated from 1000 rapid bootstraps (also using the constraint trees). Individual 18S and 28S rRNA gene phylogenies were estimated using the –*x* option with 1000 rapid bootstraps and no constraint tree. During a series of analyses, we excluded the following taxa from the original AFTOL alignments because of extremely long branches or inconsistent phylogenetic placement: *Agonimia* sp., *Bacidia schweinitzii*, *Candida lusitaniae*, *Cryptomycocolax abnormis*, *Dermatocarpon miniatum*, *Encephalitozoon cuniculi*, *Echinoplaca strigulacea*, and *Yarrowia lipolytica*. These taxa did not represent sister groups for any of our undescribed OTUs according to the initial analyses.

### Statistical analyses

**Fig. 3:**
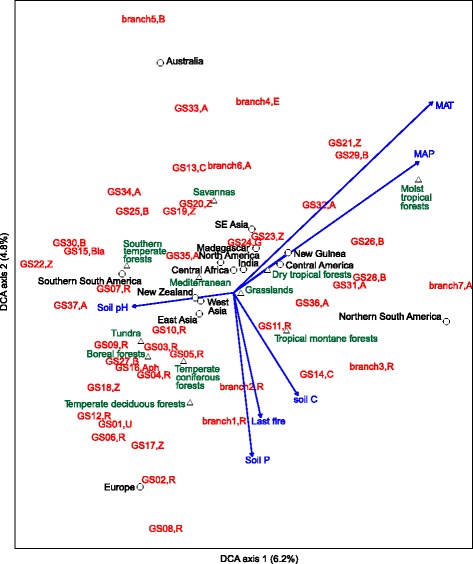
Detrended correspondence analysis biplot indicating the relative placement of novel clades and prominent branches in the combined climatic, edaphic, and biogeographic space. Clades are supplied with abbreviations of phylogenetic affinities at the phylum level: *A* Ascomycota, *Aph* Aphelida, *B* Basidiomycota, *Bla* Blastocladiomycota, *C* Chytridiomycota, *E* Entorrhizomycota, *G* Glomeromycota, *R* Rozellomycota, *Z* Zygomycota s.lat., *U* unassigned

Based on the topology of the concatenated tree, we focused on statistically supported branches (BS >70) featuring no described species. We refer to these as clades following the International Code of Phylogenetic Nomenclature [44]. We also addressed the unique branches comprising single sequences if these could not be placed to orders or classes. Each novel group (37 clades and seven branches altogether representing 819 OTUs and 9778 sequences) that comprised >1 OTU (93% of these groups) was subjected to niche analysis using a machine learning Random forest algorithm [45] by combining the randomForest [46] and VSURF [47] packages of R. This approach makes no assumptions on the distribution of residuals and type of response, which renders it suitable for analysis of very sparse data sets including large numbers of absences. For niche analysis, we compiled all information on the richness and distribution of OTUs within the above-defined clades as well as associated metadata [8]. From the initial pool of 17 edaphic, floristic, and climatic variables, we selected the six most important predictors across the whole data set, removing multicollinear and unimportant variables. In the final Random forest model selection, we thus included only mean annual temperature (MAT), mean annual precipitation (MAP), soil pH, soil P and C concentration, and time since last fire. In a separate analysis, we tested whether the distribution of clades was biased in relation to biomes and ecoregions, which were treated as categorical predictors. *P* values were calculated based on 999 data re-arrangement permutations using the rfPermute package of R [48]. To assess the efficiency of models, 10-fold cross-validation was used. The original data were randomly partitioned into 10 subsets to generate training sets and test sets. This process was repeated 100 times and revealed an *R* ^2^-cv accuracy index of models for training sets to explain test sets (Additional file 1: Figure S4). To illustrate the niches, we present the occurrence of specific OTUs within each clade compared with the null distribution of site conditions in histograms. The niche of clades was considered to be significantly narrower than expected if (i) the standard deviation of the null distribution exceeded that of OTU distribution >2-fold and (ii) the Levene test for homogeneity of variances was significant at *α* = 0.05. To visualize the relationships of clades with the climatic, edaphic, and biogeographic environment, we constructed a two-dimensional detrended correspondence analysis (DCA) ordination biplot using the occurrence of OTUs of clades and prominent branches and Bray-Curtis distance as implemented in the vegan package of R [49] (Fig. 3).

The 18S and 28S rRNA gene sequences were further compared with metadata and phylograms in the literature from which the other environmental sequences used in phylograms were obtained (Additional file 1: Table S2). These data and associated metadata were integrated for interpreting the ecological and geographic distribution of the soil-inhabiting groups. In addition, the ITS sequences of all focal taxa were compared with the 80% sequence similarity-based compound clusters in the UNITE database [50] to determine the relative identification capacity of the newly described groups against clusters of recently accumulated fungal ITS barcodes.

## Additional files

Additional file 1: Figure S1. Full concatenated 18S and 28S rRNA gene phylogram. **Figure S2.** Full 18S rRNA gene phylogram. **Figure S3.** Full 28S rRNA gene phylogram. **Figure S4.** Best models of Random forest machine learning-based niche analysis of fungal clades and prominent branches. **Figure S5.** Histograms indicating the distribution of fungal clades (summed occurrences of OTUs) in sites with specified mean annual temperature. **Figure S6.** Histograms indicating the distribution of fungal clades (summed occurrences of OTUs) in sites with specified mean annual precipitation. **Figure S7.** Histograms indicating the distribution of fungal clades (summed occurrences of OTUs) in sites with specified soil pH. **Figure S8.** Histograms indicating the distribution of fungal clades (summed occurrences of OTUs) in sites with specified time since last fire, soil carbon content, and soil phosphorus concentration. Text S1. Profiles of undescribed clades and prominent branches of fungi. (PDF 10058 kb)

Additional file 2: Table S1. Primers developed and used for this study. **Table S2.** List of environmental sources used in Additional file 1: Figures S1-S3 and accounted for in clade profiles (Text S1). Data S1. Metadata, clade assignment, and accessions of individual OTUs. (XLSX 1032 kb)
