# Supplementary material for: Novel soil-inhabiting clades fill gaps in the fungal tree of life
Source: Microbiome. 2017 Apr 8;5:42. doi: 10.1186/s40168-017-0259-5 (PMC5385062; doi:10.1186/s40168-017-0259-5)
Supplement: Supplementary file 1 — Full concatenated 18S and 28S rRNA gene phylogram. Figure S2. Full 18S rRNA gene phylogram. Figure S3. Full 28S rRNA gene phylogram. Figure S4. Best models of Random forest machine learning-based niche analysis of fungal clades and prominent branches. Figure S5. Histograms indicating the distribution of fungal clades (summed occurrences of OTUs) in sites with specified mean annual temperature. Figure S6. Histograms indicating the distribution of fungal clades (summed occurrences of OTUs) in sites with specified mean annual precipitation. Figure S7. Histograms indicating the distribution of fungal clades (summed occurrences of OTUs) in sites with specified soil pH. Figure S8. Histograms indicating the distribution of fungal clades (summed occurrences of OTUs) in sites with specified time since last fire, soil carbon content, and soil phosphorus concentration. Text S1. Profiles of undescribed clades and prominent branches of fungi. (PDF 10058 kb) [file 40168_2017_259_MOESM1_ESM.pdf]

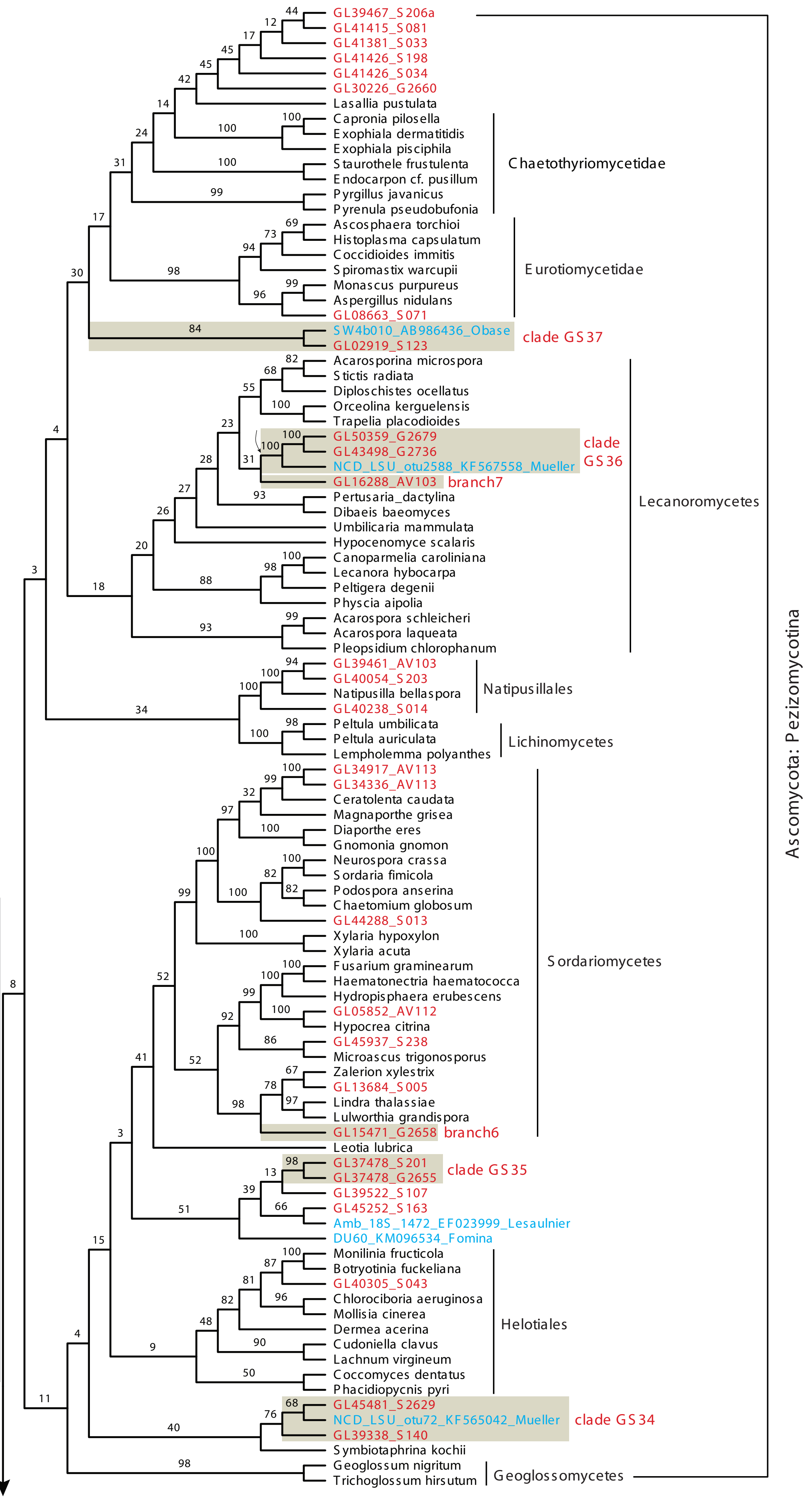

Figure S1. Phylogenetic placement of soil-inhabiting fungi among identified taxa and other sequences from the environment based on a Maximum Likelihood phylogram of full concatenated 18S and 28S rRNA genes. Red, blue, and black fonts depict groups recovered here, in other environmental sequencing studies, and based on vouchered collections, respectively. Shaded clusters indicate prominent clades and branches discussed.

Figure S1.1

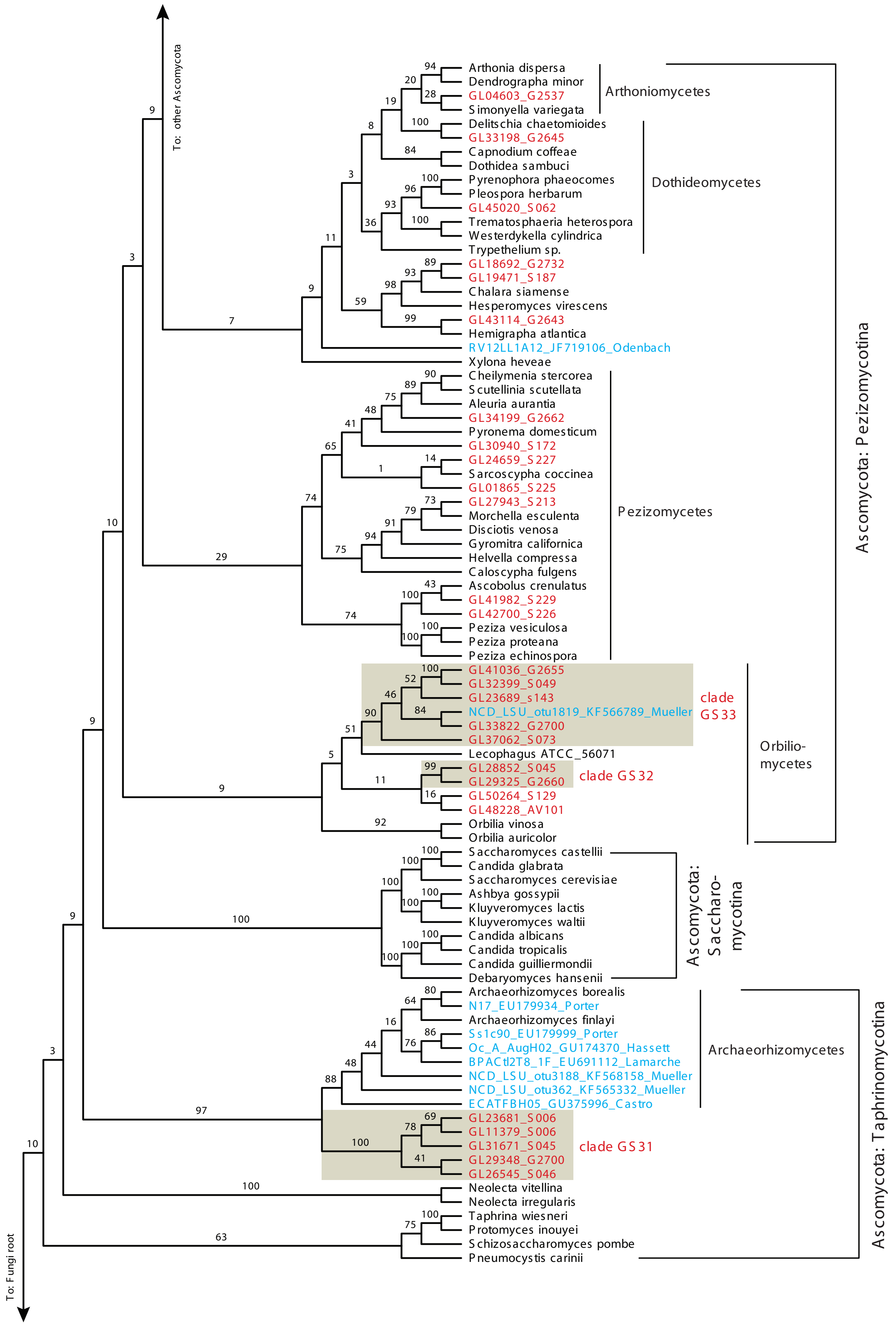
To: other Ascomycota
Arthonia dispersa
Dendrographa minor
GL04603_G2537
Simonyella variegata
Arthoniomycetes
Delitschia chaetomioides
GL33198_G2645
Capnodium coffeae
Dothidea sambuci
Pyrenophora phaeocomes
Pleospora herbarum
GL45020_S062
Trematosphaeria heterospora
Westerdykella cylindrica
Trypethelium sp.
Dothideomycetes
GL18692_G2732
GL19471_S187
Chalara siamense
Hesperomyces virescens
GL43114_G2643
Hemigrapha atlantica
RV12LL1A12_JF719106_Odenbach
Xylona heveae
Cheilymenia stercorea
Scutellinia scutellata
Aleuria aurantia
GL34199_G2662
Pyronema domesticum
GL30940_S172
GL24659_S227
Sarcoscypha coccinea
GL01865_S225
GL27943_S213
Morchella esculenta
Disciotis venosa
Gyromitra californica
Helvella compressa
Caloscypha fulgens
Ascobolus crenulatus
GL41982_S229
GL42700_S226
Peziza vesiculosa
Peziza proteana
Peziza echinospora
Pezizomycetes
GL41036_G2655
GL32399_S049
GL23689_s143
NCD_LSU_otu1819_KF566789_Mueller
GL33822_G2700
GL37062_S073
clade GS33
Lecophagus ATCC_56071
GL28852_S045
GL29325_G2660
clade GS32
GL50264_S129
GL48228_AV101
Orbilia vinosa
Orbilia auricolor
Orbilio-mycetes
Ascomycota: Pezizomycotina
Saccharomyces castellii
Candida glabrata
Saccharomyces cerevisiae
Ashbya gossypii
Kluyveromyces lactis
Kluyveromyces waltii
Candida albicans
Candida tropicalis
Candida guilliermondii
Debaryomyces hansenii
Ascomycota: Saccharo-mycotina
Archaeorhizomyces borealis
N17_EU179934_Porter
Archaeorhizomyces finlayi
Ss1c90_EU179999_Porter
Oc_A_AugH02_GU174370_Hassett
BPACtl2T8_1F_EU691112_Lamarche
NCD_LSU_otu3188_KF568158_Mueller
NCD_LSU_otu362_KF565332_Mueller
ECATFBH05_GU375996_Castro
Archaeorhizomycetes
GL23681_S006
GL11379_S006
GL31671_S045
GL29348_G2700
GL26545_S046
clade GS31
Neolecta vitellina
Neolecta irregularis
Taphrina wiesneri
Protomyces inouyei
Schizosaccharomyces pombe
Pneumocystis carinii
Ascomycota: Taphrinomycotina
To: Fungi root

Figure S1.2

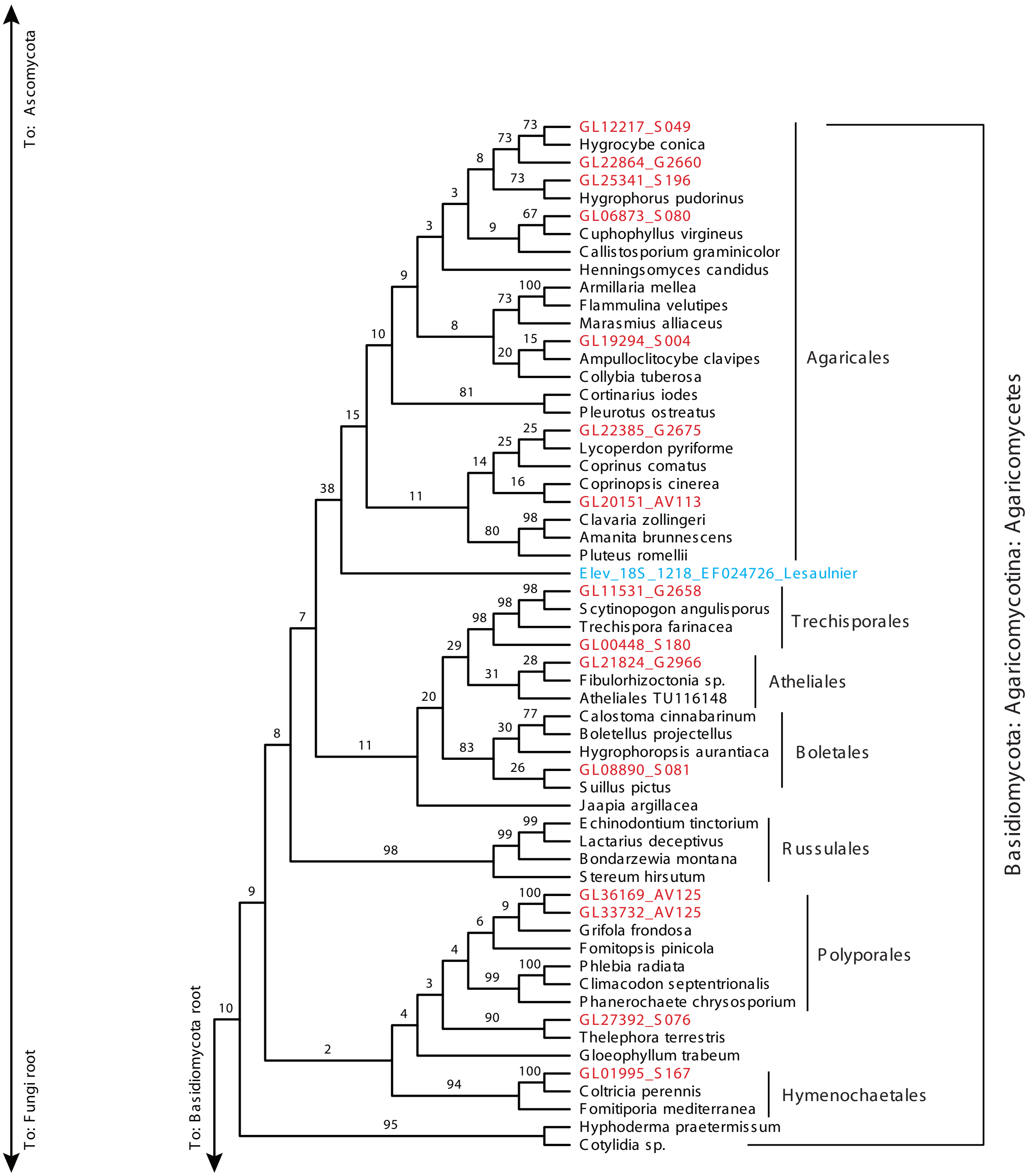
To: Ascomycota
To: Fungi root
To: Basidiomycota root
GL12217_S049
Hygrocybe conica
GL22864_G2660
GL25341_S196
Hygrophorus pudorinus
GL06873_S080
Cuphophyllus virgineus
Callistosporium graminicolor
Henningsomyces candidus
Armillaria mellea
Flammulina velutipes
Marasmius alliaceus
GL19294_S004
Ampulloclitocybe clavipes
Collybia tuberosa
Cortinarius iodes
Pleurotus ostreatus
GL22385_G2675
Lycoperdon pyriforme
Coprinus comatus
Coprinopsis cinerea
GL20151_AV113
Clavaria zollingeri
Amanita brunnescens
Pluteus romellii
Elev_18S_1218_EF024726_Lesaulnier
GL11531_G2658
Scytinopogon angulisporus
Trechispora farinacea
GL00448_S180
GL21824_G2966
Fibulorhizoctonia sp.
Atheliales TU116148
Calostoma cinnabarinum
Boletellus projectellus
Hygrophoropsis aurantiaca
GL08890_S081
Suillus pictus
Jaapia argillacea
Echinodontium tinctorium
Lactarius deceptivus
Bondarzewia montana
Stereum hirsutum
GL36169_AV125
GL33732_AV125
Grifola frondosa
Fomitopsis pinicola
Phlebia radiata
Climacodon septentrionalis
Phanerochaete chrysosporium
GL27392_S076
Thelephora terrestris
Gloeophyllum trabeum
GL01995_S167
Coltricia perennis
Fomitiporia mediterranea
Hyphoderma praetermissum
Cotylidia sp.
Agaricales
Trechisporales
Atheliales
Boletales
Russulales
Polyporales
Hymenochaetales
Basidiomycota: Agaricomycotina: Agaricomycetes

Figure S1.3

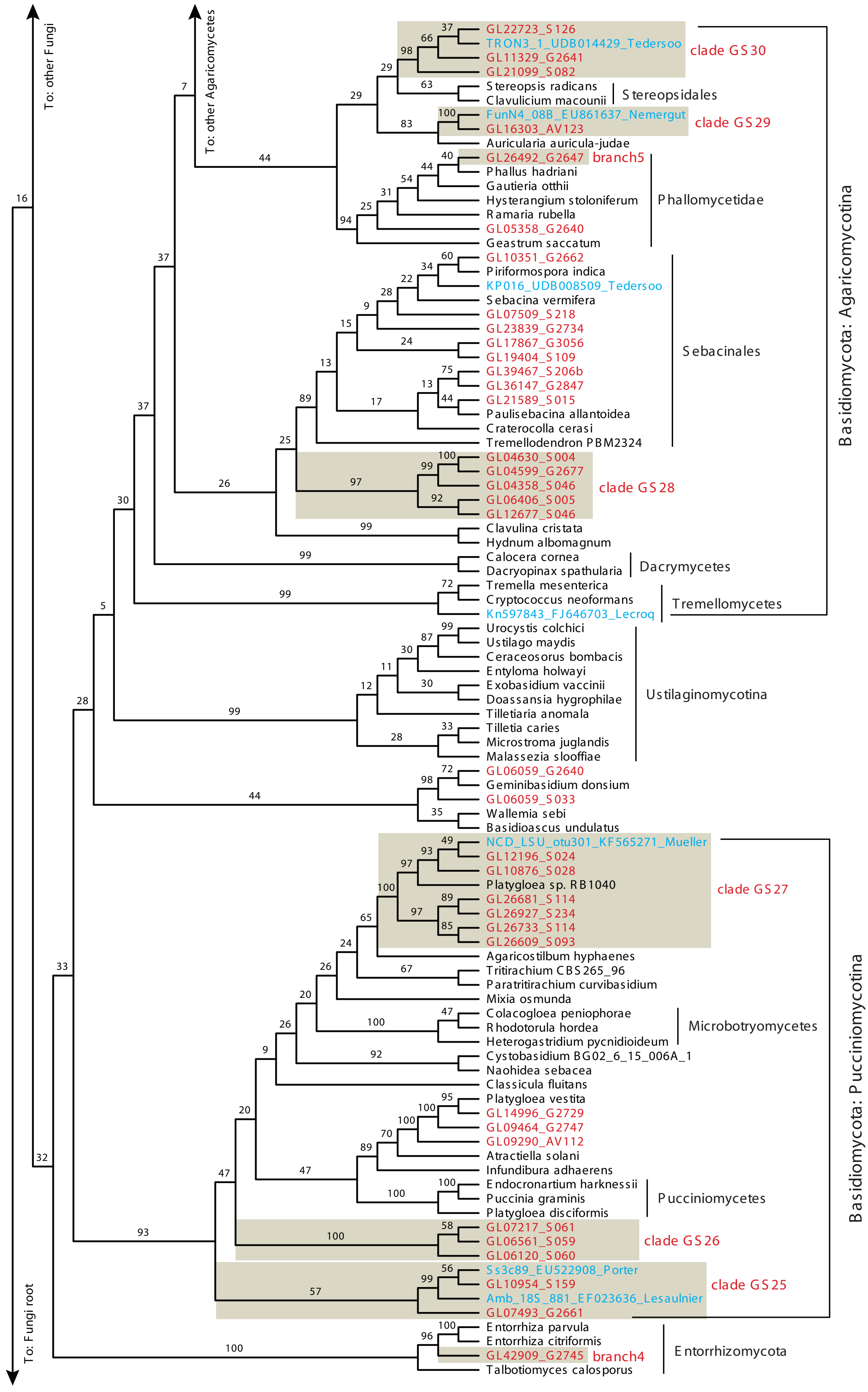

To: other Fungi
To: other Agaricomycetes
GL22723_S126
TRON3_1_UDB014429_Tedersoo
GL11329_G2641
GL21099_S082
clade GS30
Stereopsis radicans
Clavulicium macounii
Stereopsidales
FunN4_08B_EU861637_Nemergut
GL16303_AV123
clade GS29
Auricularia auricula-judae
GL26492_G2647
branch5
Phallus hadriani
Gautieria otthii
Hysterangium stoloniferum
Ramaria rubella
GL05358_G2640
Geastrum saccatum
Phallomycetidae
GL10351_G2662
Piriformospora indica
KP016_UDB008509_Tedersoo
Sebacina vermifera
GL07509_S218
GL23839_G2734
GL17867_G3056
GL19404_S109
GL39467_S206b
GL36147_G2847
GL21589_S015
Paulisebacina allantoidea
Craterocolla cerasi
Tremellodendron PBM2324
Sebacinales
GL04630_S004
GL04599_G2677
GL04358_S046
GL06406_S005
GL12677_S046
clade GS28
Clavulina cristata
Hydnum albomagnum
Calocera cornea
Dacryopinax spathularia
Dacrymycetes
Tremella mesenterica
Cryptococcus neoformans
Kn597843_FJ646703_Lecroq
Tremellomycetes
Basidiomycota: Agaricomycotina
Urocystis colchici
Ustilago maydis
Ceraceosorus bombacis
Entyloma holwayi
Exobasidium vaccinii
Doassansia hygrophilae
Tilletiaria anomala
Tilletia caries
Microstroma juglandis
Malassezia slooffiae
Ustilaginomycotina
GL06059_G2640
Geminibasidium donsium
GL06059_S033
Wallemia sebi
Basidioascus undulatus
NCD_LSU_otu301_KF565271_Mueller
GL12196_S024
GL10876_S028
Platygloea sp. RB1040
GL26681_S114
GL26927_S234
GL26733_S114
GL26609_S093
clade GS27
Agaricostilbum hyphaenes
Tritirachium CBS265_96
Paratritirachium curvibasidium
Mixia osmunda
Colacogloea peniophorae
Rhodotorula hordea
Heterogastridium pycnidioideum
Microbotryomycetes
Cystobasidium BG02_6_15_006A_1
Naohidea sebacea
Classicula fluitans
Platygloea vestita
GL14996_G2729
GL09464_G2747
GL09290_AV112
Atractiella solani
Infundibura adhaerens
Endocronartium harknessii
Puccinia graminis
Platygloea disciformis
Pucciniomycetes
GL07217_S061
GL06561_S059
GL06120_S060
clade GS26
Ss3c89_EU522908_Porter
GL10954_S159
Amb_18S_881_EF023636_Lesaulnier
GL07493_G2661
clade GS25
Basidiomycota: Pucciniomycotina
Entorrhiza parvula
Entorrhiza citriformis
GL42909_G2745
branch4
Talbotiomyces calosporus
Entorrhizomycota
To: Fungi root

Figure S1.4

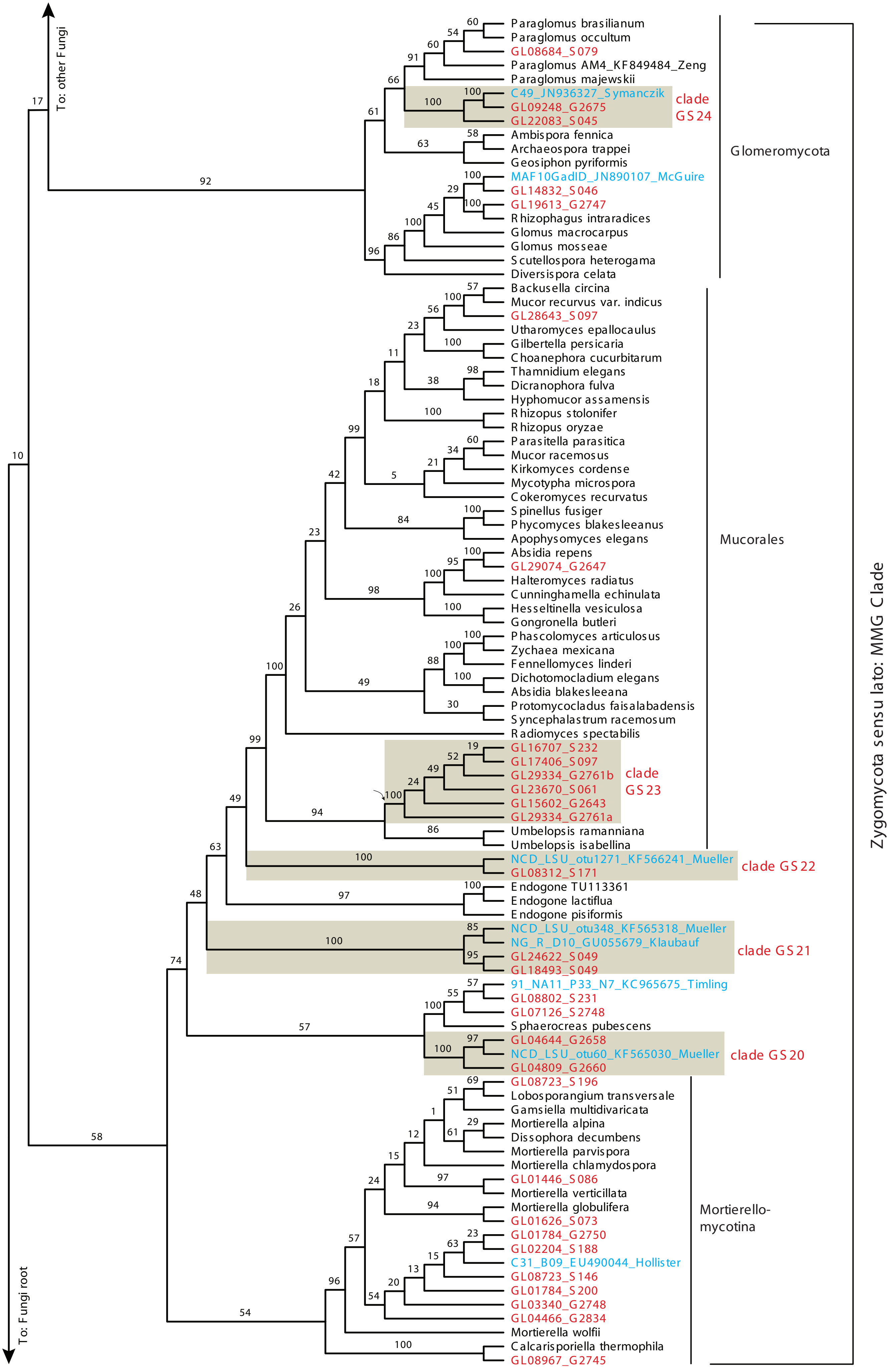
To: other Fungi
To: Fungi root
Paraglomus brasilianum
Paraglomus occultum
GL08684_S079
Paraglomus AM4_KF849484_Zeng
Paraglomus majewskii
C49_JN936327_Symanczik
GL09248_G2675
GL22083_S045
clade GS24
Ambispora fennica
Archaeospora trappei
Geosiphon pyriformis
MAF10GadID_JN890107_McGuire
GL14832_S046
GL19613_G2747
Rhizophagus intraradices
Glomus macrocarpus
Glomus mosseae
Scutellospora heterogama
Diversispora celata
Glomeromycota
Backusella circina
Mucor recurvus var. indicus
GL28643_S097
Utharomyces epallocaulus
Gilbertella persicaria
Choanephora cucurbitarum
Thamnidium elegans
Dicranophora fulva
Hyphomucor assamensis
Rhizopus stolonifer
Rhizopus oryzae
Parasitella parasitica
Mucor racemosus
Kirkomyces cordense
Mycotypha microspora
Cokeromyces recurvatus
Spinellus fusiger
Phycomyces blakesleeanus
Apophysomyces elegans
Absidia repens
GL29074_G2647
Halteromyces radiatus
Cunninghamella echinulata
Hesseltinella vesiculosa
Gongronella butleri
Phascolomyces articulosus
Zychaea mexicana
Fennellomyces linderi
Dichotomocladium elegans
Absidia blakesleeana
Protomycocladus faisalabadensis
Syncephalastrum racemosum
Radiomyces spectabilis
Mucorales
GL16707_S232
GL17406_S097
GL29334_G2761b
GL23670_S061
GL15602_G2643
GL29334_G2761a
clade GS23
Umbelopsis ramanniana
Umbelopsis isabellina
NCD_LSU_otu1271_KF566241_Mueller
GL08312_S171
clade GS22
Endogone TU113361
Endogone lactiflua
Endogone pisiformis
NCD_LSU_otu348_KF565318_Mueller
NG_R_D10_GU055679_Klaubauf
GL24622_S049
GL18493_S049
clade GS21
91_NA11_P33_N7_KC965675_Timling
GL08802_S231
GL07126_S2748
Sphaerocreas pubescens
GL04644_G2658
NCD_LSU_otu60_KF565030_Mueller
GL04809_G2660
clade GS20
GL08723_S196
Lobosporangium transversale
Gamsiella multidivaricata
Mortierella alpina
Dissophora decumbens
Mortierella parvispora
Mortierella chlamydospora
GL01446_S086
Mortierella verticillata
Mortierella globulifera
GL01626_S073
GL01784_G2750
GL02204_S188
C31_B09_EU490044_Hollister
GL08723_S146
GL01784_S200
GL03340_G2748
GL04466_G2834
Mortierella wolfii
Calcarisporiella thermophila
GL08967_G2745
Mortierello-
mycotina
Zygomycota sensu lato: MMG Clade

Figure S1.5

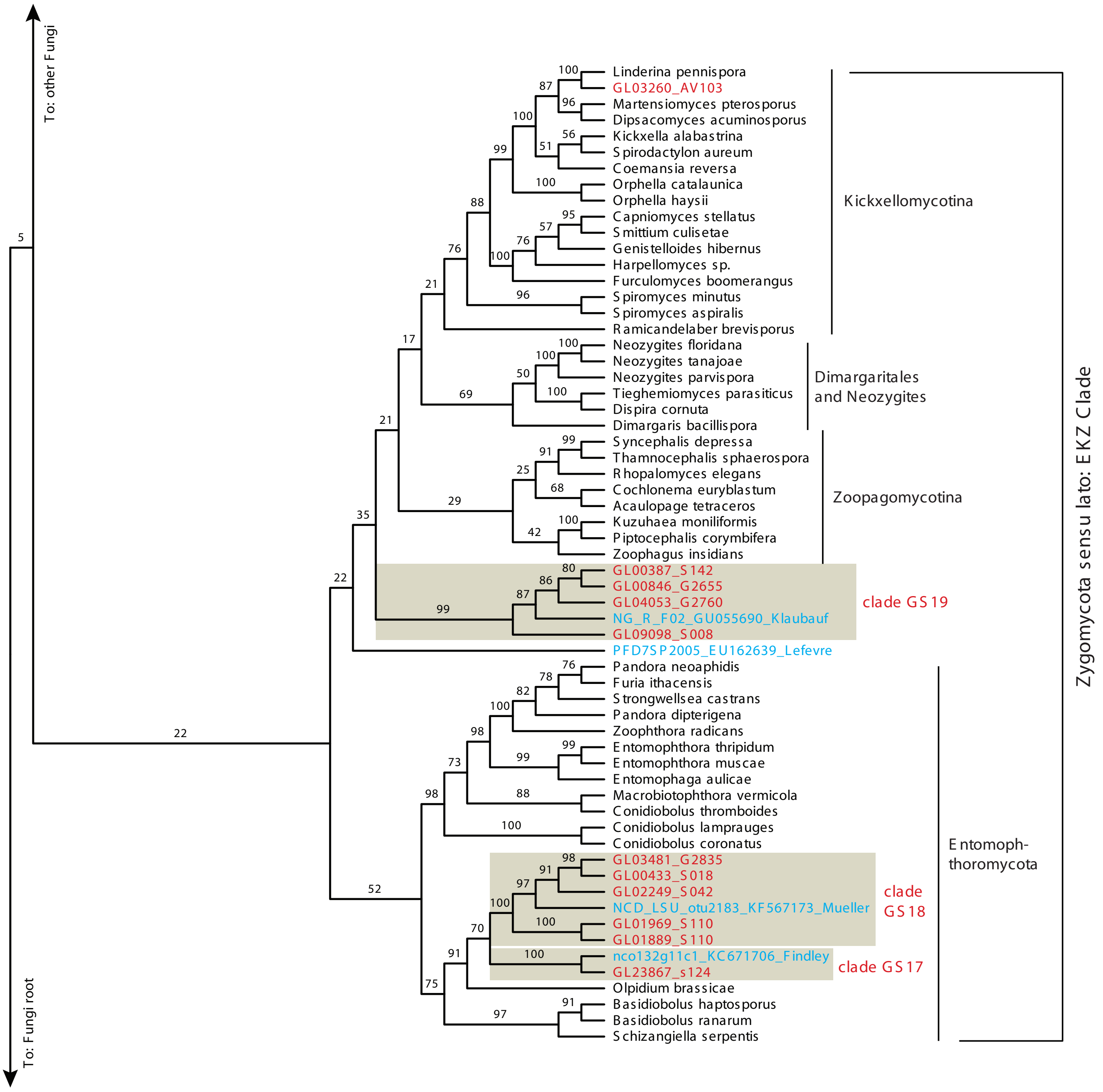
To: other Fungi
To: Fungi root
Linderina pennispora
GL03260_AV103
Martensiomyces pterosporus
Dipsacomyces acuminosporus
Kickxella alabastrina
Spirodactylon aureum
Coemansia reversa
Orphella catalaunica
Orphella haysii
Capniomyces stellatus
Smittium culisetae
Genistelloides hibernus
Harpellomyces sp.
Furculomyces boomerangus
Spiromyces minutus
Spiromyces aspiralis
Ramicandelaber brevisporus
Neozygites floridana
Neozygites tanajoae
Neozygites parvispora
Tieghemiomyces parasiticus
Dispira cornuta
Dimargaris bacillispora
Syncephalis depressa
Thamnocephalis sphaerospora
Rhopalomyces elegans
Cochlonema euryblastum
Acaulopage tetraceros
Kuzuhaea moniliformis
Piptocephalis corymbifera
Zoophagus insidians
GL00387_S142
GL00846_G2655
GL04053_G2760
NG_R_F02_GU055690_Klaubauf
GL09098_S008
PFD7SP2005_EU162639_Lefevre
Pandora neoaphidis
Furia ithacensis
Strongwellsea castrans
Pandora dipterigena
Zoophthora radicans
Entomophthora thripidum
Entomophthora muscae
Entomophaga aulicae
Macrobiotophthora vermicola
Conidiobolus thromboides
Conidiobolus lamprauges
Conidiobolus coronatus
GL03481_G2835
GL00433_S018
GL02249_S042
NCD_LSU_otu2183_KF567173_Mueller
GL01969_S110
GL01889_S110
nco132g11c1_KC671706_Findley
GL23867_s124
Olpidium brassicae
Basidiobolus haptosporus
Basidiobolus ranarum
Schizangiella serpentis
Kickxellomycotina
Dimargaritales and Neozygites
Zoopagomycotina
clade GS19
clade GS18
clade GS17
Entomoph-thoromycota
Zygomycota sensu lato: EKZ Clade

Figure S1.6

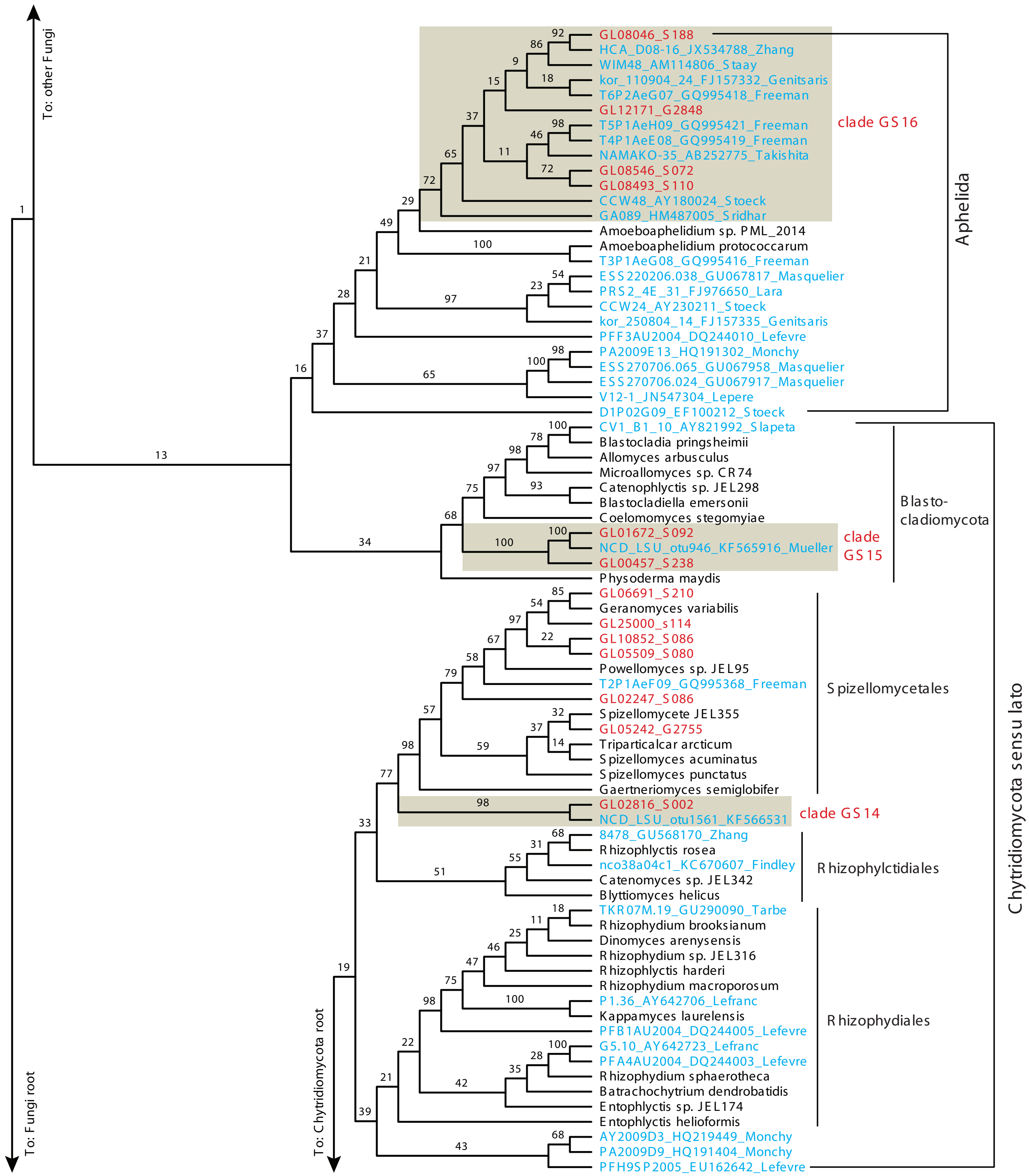
To: other Fungi
To: Fungi root
To: Chytridiomycota root
GL08046_S188
HCA_D08-16_JX534788_Zhang
WIM48_AM114806_Staay
kor_110904_24_FJ157332_Genitsaris
T6P2AeG07_GQ995418_Freeman
GL12171_G2848
T5P1AeH09_GQ995421_Freeman
T4P1AeE08_GQ995419_Freeman
NAMAKO-35_AB252775_Takishita
GL08546_S072
GL08493_S110
CCW48_AY180024_Stoeck
GA089_HM487005_Sridhar
clade GS16
Amoebaphelidium sp. PML_2014
Amoebaphelidium protococcarum
T3P1AeG08_GQ995416_Freeman
ESS220206.038_GU067817_Masquelier
PRS2_4E_31_FJ976650_Lara
CCW24_AY230211_Stoeck
kor_250804_14_FJ157335_Genitsaris
PFF3AU2004_DQ244010_Lefevre
PA2009E13_HQ191302_Monchy
ESS270706.065_GU067958_Masquelier
ESS270706.024_GU067917_Masquelier
V12-1_JN547304_Lepere
D1P02G09_EF100212_Stoeck
Aphelida
CV1_B1_10_AY821992_Slapeta
Blastocladia pringsheimii
Allomyces arbusculus
Microallomyces sp. CR74
Catenophlyctis sp. JEL298
Blastocladiella emersonii
Coelomomyces stegomyiae
GL01672_S092
NCD_LSU_otu946_KF565916_Mueller
GL00457_S238
clade GS15
Physoderma maydis
Blasto-cladiomycota
GL06691_S210
Geranomyces variabilis
GL25000_s114
GL10852_S086
GL05509_S080
Powellomyces sp. JEL95
T2P1AeF09_GQ995368_Freeman
GL02247_S086
Spizellomycete JEL355
GL05242_G2755
Triparticalcar arcticum
Spizellomyces acuminatus
Spizellomyces punctatus
Gaertneriomyces semiglobifer
Spizellomycetales
GL02816_S002
NCD_LSU_otu1561_KF566531
clade GS14
8478_GU568170_Zhang
Rhizophlyctis rosea
nco38a04c1_KC670607_Findley
Catenomyces sp. JEL342
Blyttiomyces helicus
Rhizophylctidiales
TKR07M.19_GU290090_Tarbe
Rhizophydium brooksianum
Dinomyces arenysensis
Rhizophydium sp. JEL316
Rhizophlyctis harderi
Rhizophydium macroporosum
P1.36_AY642706_Lefranc
Kappamyces laurelensis
PFB1AU2004_DQ244005_Lefevre
G5.10_AY642723_Lefranc
PFA4AU2004_DQ244003_Lefevre
Rhizophydium sphaerotheca
Batrachochytrium dendrobatidis
Entophlyctis sp. JEL174
Entophlyctis helioformis
Rhizophydiales
AY2009D3_HQ219449_Monchy
PA2009D9_HQ191404_Monchy
PFH9SP2005_EU162642_Lefevre
Chytridiomycota sensu lato

Figure S1.7

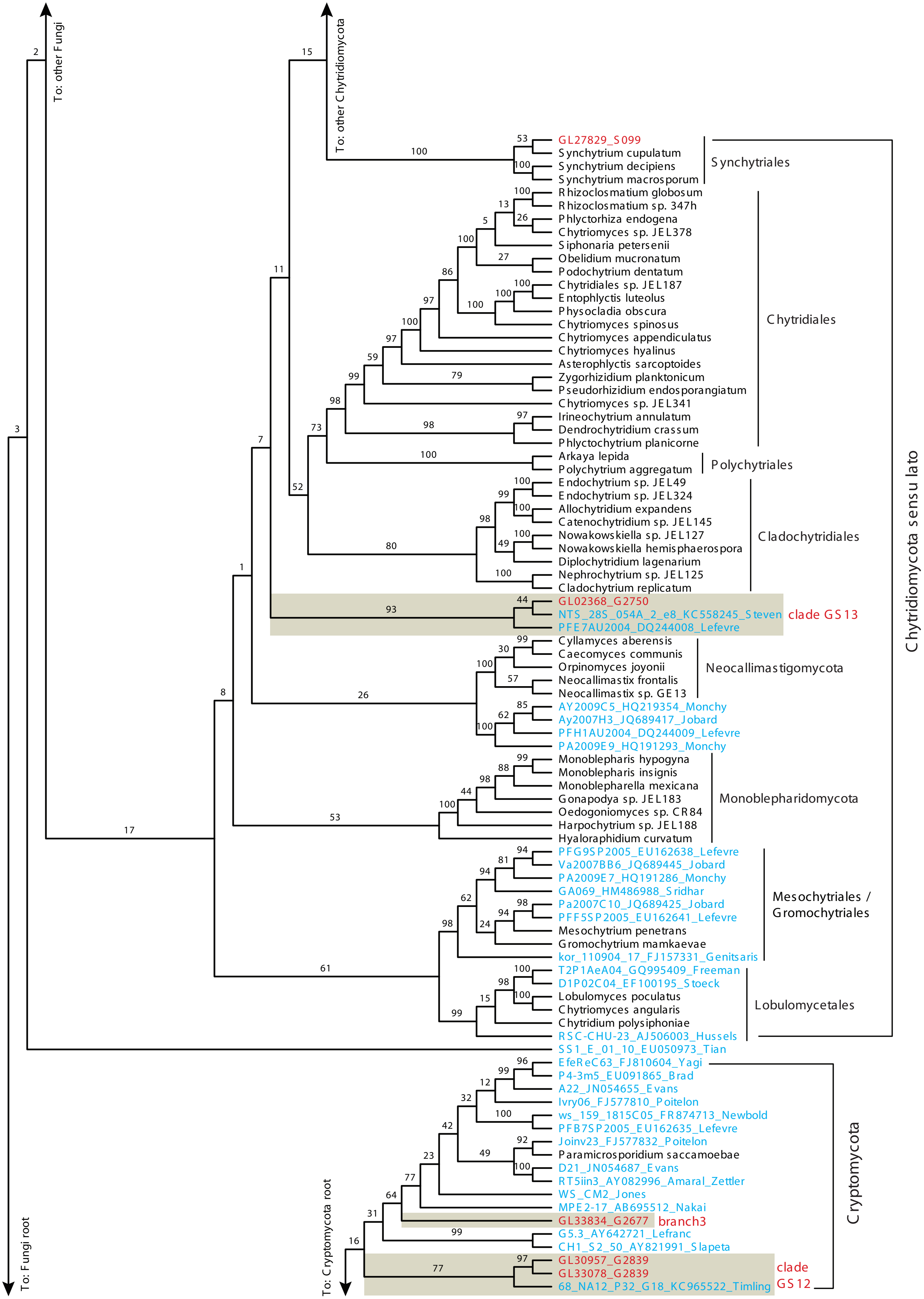
To: other Fungi
To: other Chytridiomycota
GL27829_S099
Synchytrium cupulatum
Synchytrium decipiens
Synchytrium macrosporum
Synchytriales
Rhizoclosmatium globosum
Rhizoclosmatium sp. 347h
Phlyctorhiza endogena
Chytriomyces sp. JEL378
Siphonaria petersenii
Obelidium mucronatum
Podochytrium dentatum
Chytridiales sp. JEL187
Entophlyctis luteolus
Physocladia obscura
Chytriomyces spinosus
Chytriomyces appendiculatus
Chytriomyces hyalinus
Asterophlyctis sarcoptoides
Zygorhizidium planktonicum
Pseudorhizidium endosporangiatum
Chytriomyces sp. JEL341
Irineochytrium annulatum
Dendrochytridium crassum
Phlyctochytrium planicorne
Chytridiales
Arkaya lepida
Polychytrium aggregatum
Polychytriales
Endochytrium sp. JEL49
Endochytrium sp. JEL324
Allochytridium expandens
Catenochytridium sp. JEL145
Nowakowskiella sp. JEL127
Nowakowskiella hemisphaerospora
Diplochytridium lagenarium
Nephrochytrium sp. JEL125
Cladochytrium replicatum
Cladochytridiales
GL02368_G2750
NTS_28S_054A_2_e8_KC558245_Steven
PFE7AU2004_DQ244008_Lefevre
clade GS13
Cyllamyces aberensis
Caecomyces communis
Orpinomyces joyonii
Neocallimastix frontalis
Neocallimastix sp. GE13
Neocallimastigomycota
AY2009C5_HQ219354_Monchy
Ay2007H3_JQ689417_Jobard
PFH1AU2004_DQ244009_Lefevre
PA2009E9_HQ191293_Monchy
Monoblepharis hypogyna
Monoblepharis insignis
Monoblepharella mexicana
Gonapodya sp. JEL183
Oedogoniomyces sp. CR84
Harpochytrium sp. JEL188
Hyaloraphidium curvatum
Monoblepharidomycota
PFG9SP2005_EU162638_Lefevre
Va2007BB6_JQ689445_Jobard
PA2009E7_HQ191286_Monchy
GA069_HM486988_Sridhar
Pa2007C10_JQ689425_Jobard
PFF5SP2005_EU162641_Lefevre
Mesochytrium penetrans
Gromochytrium mamkaevae
kor_110904_17_FJ157331_Genitsaris
Mesochytriales / Gromochytriales
T2P1AeA04_GQ995409_Freeman
D1P02C04_EF100195_Stoeck
Lobulomyces poculatus
Chytriomyces angularis
Chytridium polysiphoniae
RSC-CHU-23_AJ506003_Hussels
Lobulomycetales
Chytridiomycota sensu lato
SS1_E_01_10_EU050973_Tian
EfeReC63_FJ810604_Yagi
P4-3m5_EU091865_Brad
A22_JN054655_Evans
Ivry06_FJ577810_Poitelon
ws_159_1815C05_FR874713_Newbold
PFB7SP2005_EU162635_Lefevre
Joinv23_FJ577832_Poitelon
Paramicrosporidium saccamoebae
D21_JN054687_Evans
RT5iin3_AY082996_Amaral_Zettler
WS_CM2_Jones
MPE2-17_AB695512_Nakai
GL33834_G2677
branch3
G5.3_AY642721_Lefranc
CH1_S2_50_AY821991_Slapeta
GL30957_G2839
GL33078_G2839
68_NA12_P32_G18_KC965522_Timling
clade GS12
Cryptomycota
To: Fungi root
To: Cryptomycota root

Figure S1.8

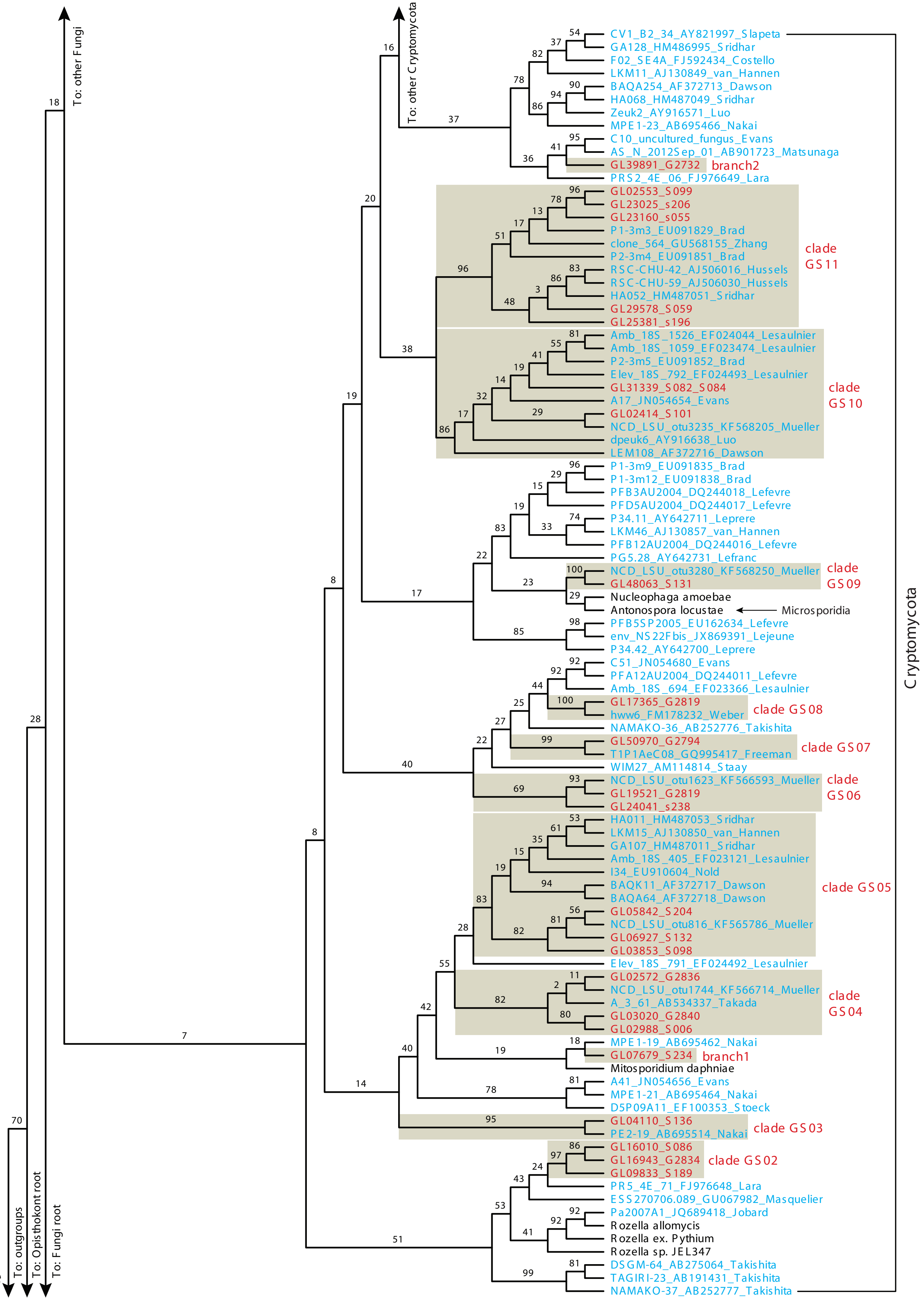

CV1_B2_34_AY821997_Slapeta
GA128_HM486995_Sridhar
F02_SE4A_FJ592434_Costello
LKM11_AJ130849_van_Hannen
BAQA254_AF372713_Dawson
HA068_HM487049_Sridhar
Zeuk2_AY916571_Luo
MPE1-23_AB695466_Nakai
C10_uncultured_fungus_Evans
AS_N_2012Sep_01_AB901723_Matsunaga
GL39891_G2732
branch2
PRS2_4E_06_FJ976649_Lara
GL02553_S099
GL23025_s206
GL23160_s055
P1-3m3_EU091829_Brad
clone_564_GU568155_Zhang
P2-3m4_EU091851_Brad
RSC-CHU-42_AJ506016_Hussels
RSC-CHU-59_AJ506030_Hussels
HA052_HM487051_Sridhar
GL29578_S059
GL25381_s196
clade GS11
Amb_18S_1526_EF024044_Lesaulnier
Amb_18S_1059_EF023474_Lesaulnier
P2-3m5_EU091852_Brad
Elev_18S_792_EF024493_Lesaulnier
GL31339_S082_S084
A17_JN054654_Evans
GL02414_S101
NCD_LSU_otu3235_KF568205_Mueller
dpeuk6_AY916638_Luo
LEM108_AF372716_Dawson
clade GS10
P1-3m9_EU091835_Brad
P1-3m12_EU091838_Brad
PFB3AU2004_DQ244018_Lefevre
PFD5AU2004_DQ244017_Lefevre
P34.11_AY642711_Leprere
LKM46_AJ130857_van_Hannen
PFB12AU2004_DQ244016_Lefevre
PG5.28_AY642731_Lefranc
NCD_LSU_otu3280_KF568250_Mueller
GL48063_S131
clade GS09
Nucleophaga amoebae
Antonospora locustae
Microsporidia
PFB5SP2005_EU162634_Lefevre
env_NS22Fbis_JX869391_Lejeune
P34.42_AY642700_Leprere
C51_JN054680_Evans
PFA12AU2004_DQ244011_Lefevre
Amb_18S_694_EF023366_Lesaulnier
GL17365_G2819
hww6_FM178232_Weber
clade GS08
NAMAKO-36_AB252776_Takishita
GL50970_G2794
T1P1AeC08_GQ995417_Freeman
clade GS07
WIM27_AM114814_Staay
NCD_LSU_otu1623_KF566593_Mueller
GL19521_G2819
GL24041_s238
clade GS06
HA011_HM487053_Sridhar
LKM15_AJ130850_van_Hannen
GA107_HM487011_Sridhar
Amb_18S_405_EF023121_Lesaulnier
I34_EU910604_Nold
BAQK11_AF372717_Dawson
BAQA64_AF372718_Dawson
GL05842_S204
NCD_LSU_otu816_KF565786_Mueller
GL06927_S132
GL03853_S098
clade GS05
Elev_18S_791_EF024492_Lesaulnier
GL02572_G2836
NCD_LSU_otu1744_KF566714_Mueller
A_3_61_AB534337_Takada
GL03020_G2840
GL02988_S006
clade GS04
MPE1-19_AB695462_Nakai
GL07679_S234
branch1
Mitosporidium daphniae
A41_JN054656_Evans
MPE1-21_AB695464_Nakai
D5P09A11_EF100353_Stoeck
GL04110_S136
PE2-19_AB695514_Nakai
clade GS03
GL16010_S086
GL16943_G2834
GL09833_S189
clade GS02
PR5_4E_71_FJ976648_Lara
ESS270706.089_GU067982_Masquelier
Pa2007A1_JQ689418_Jobard
Rozella allomycis
Rozella ex. Pythium
Rozella sp. JEL347
DSGM-64_AB275064_Takishita
TAGIRI-23_AB191431_Takishita
NAMAKO-37_AB252777_Takishita
Cryptomycota
To: other Fungi
To: other Cryptomycota
To: outgroups
To: Opisthokont root
To: Fungi root

Figure S1.9

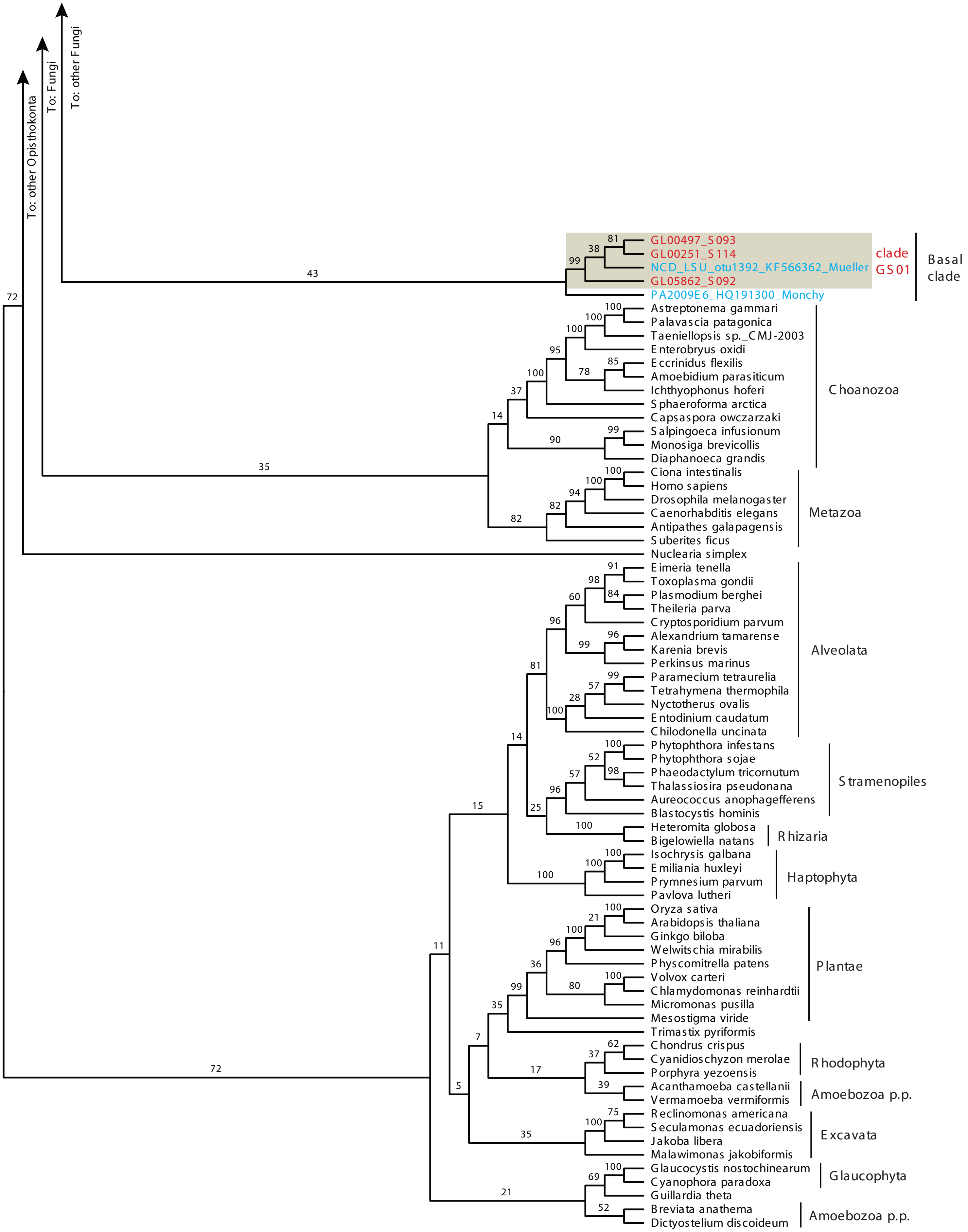
To: other Opisthokonta
To: Fungi
To: other Fungi
GL00497_S093
GL00251_S114
NCD_LSU_otu1392_KF566362_Mueller
GL05862_S092
PA2009E6_HQ191300_Monchy
clade GS01
Basal clade
Astreptonema gammari
Palavascia patagonica
Taeniellopsis sp._CMJ-2003
Enterobryus oxidi
Eccrinidus flexilis
Amoebidium parasiticum
Ichthyophonus hoferi
Sphaeroforma arctica
Capsaspora owczarzaki
Salpingoeca infusionum
Monosiga brevicollis
Diaphanoeca grandis
Choanozoa
Ciona intestinalis
Homo sapiens
Drosophila melanogaster
Caenorhabditis elegans
Antipathes galapagensis
Suberites ficus
Metazoa
Nuclearia simplex
Eimeria tenella
Toxoplasma gondii
Plasmodium berghei
Theileria parva
Cryptosporidium parvum
Alexandrium tamarense
Karenia brevis
Perkinsus marinus
Paramecium tetraurelia
Tetrahymena thermophila
Nyctotherus ovalis
Entodinium caudatum
Chilodonella uncinata
Alveolata
Phytophthora infestans
Phytophthora sojae
Phaeodactylum tricornutum
Thalassiosira pseudonana
Aureococcus anophagefferens
Blastocystis hominis
Stramenopiles
Heteromita globosa
Bigelowiella natans
Rhizaria
Isochrysis galbana
Emiliania huxleyi
Prymnesium parvum
Pavlova lutheri
Haptophyta
Oryza sativa
Arabidopsis thaliana
Ginkgo biloba
Welwitschia mirabilis
Physcomitrella patens
Volvox carteri
Chlamydomonas reinhardtii
Micromonas pusilla
Mesostigma viride
Plantae
Trimastix pyriformis
Chondrus crispus
Cyanidioschyzon merolae
Porphyra yezoensis
Rhodophyta
Acanthamoeba castellanii
Vermamoeba vermiformis
Amoebozoa p.p.
Reclinomonas americana
Seculamonas ecuadoriensis
Jakoba libera
Malawimonas jakobiformis
Excavata
Glaucocystis nostochinearum
Cyanophora paradoxa
Glaucophyta
Guillardia theta
Breviata anathema
Dictyostelium discoideum
Amoebozoa p.p.

Figure S1.10

Figure S2. Phylogenetic placement of soil-inhabiting fungi among identified taxa and other sequences from the environment based on a full 18S rRNA gene Maximum Likelihood phylogram. Red, blue, and black fonts depict groups recovered here, in other environmental sequencing studies and based on vouchered collections, respectively. Shaded clusters indicate prominent clades and branches discussed.

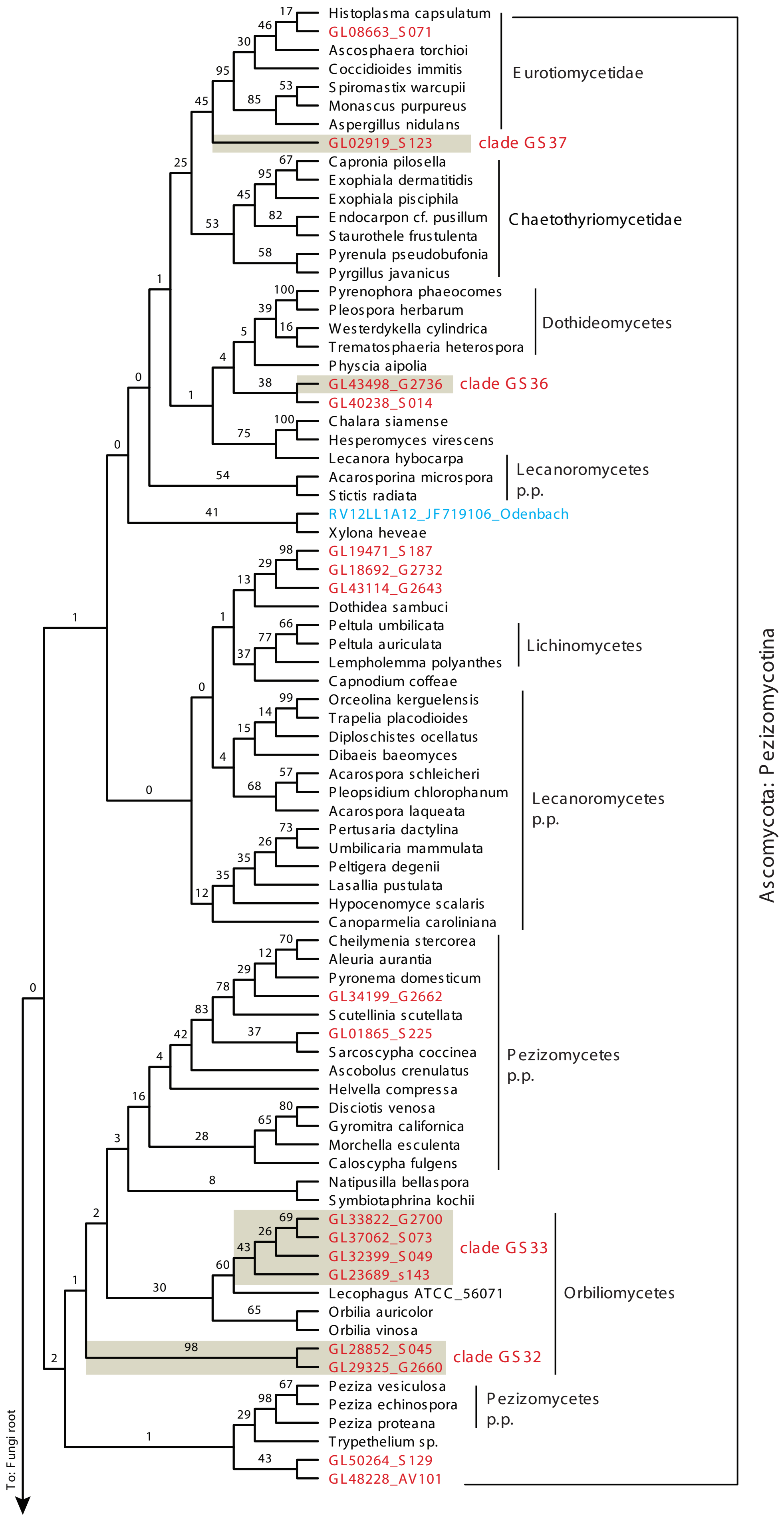

Figure S2.1

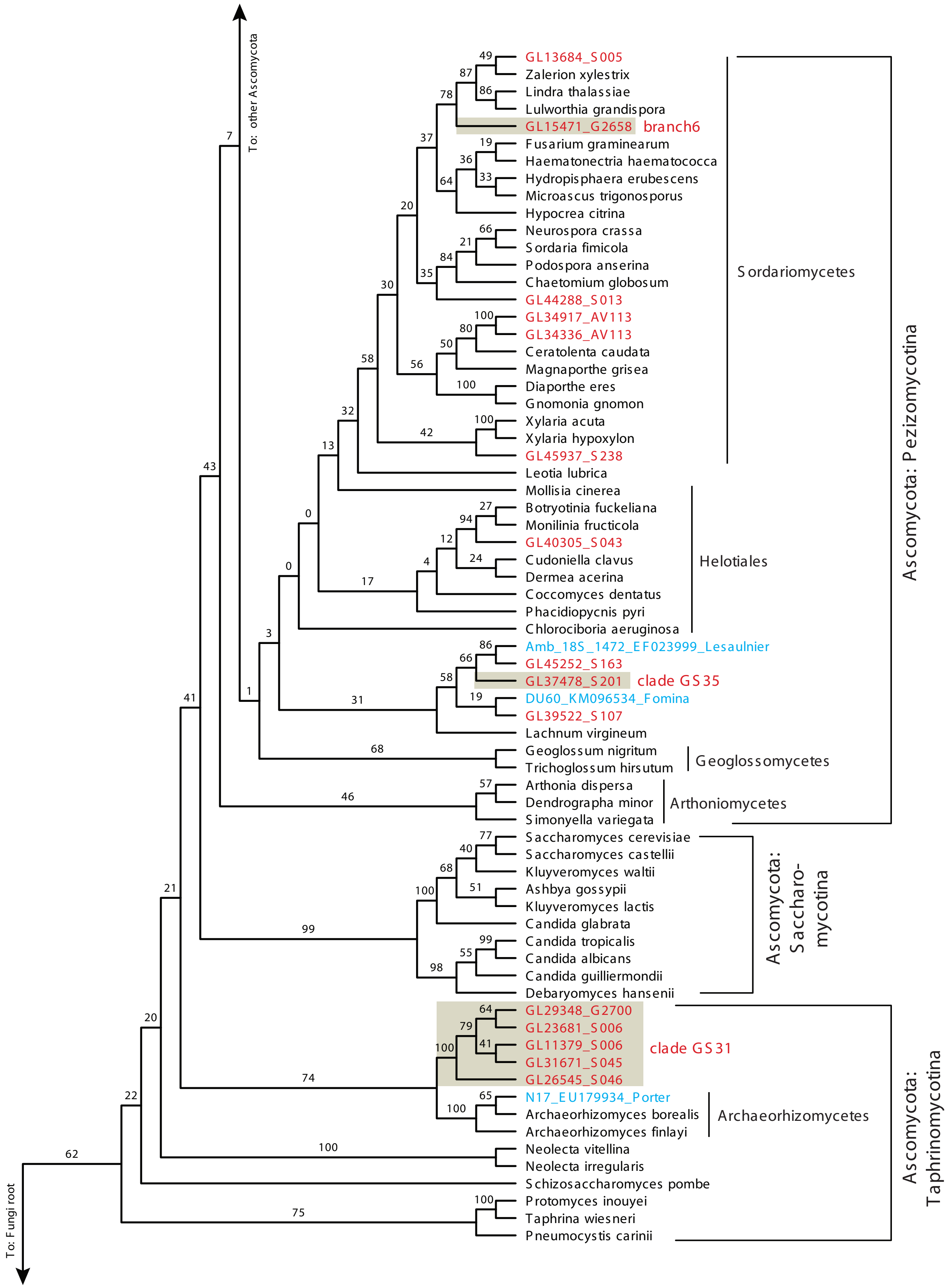
To: other Ascomycota
GL13684_S005
Zalerion xylestrix
Lindra thalassiae
Lulworthia grandispora
GL15471_G2658
branch6
Fusarium graminearum
Haematonectria haematococca
Hydropisphaera erubescens
Microascus trigonosporus
Hypocrea citrina
Neurospora crassa
Sordaria fimicola
Podospora anserina
Chaetomium globosum
GL44288_S013
GL34917_AV113
GL34336_AV113
Ceratolenta caudata
Magnaporthe grisea
Diaporthe eres
Gnomonia gnomon
Xylaria acuta
Xylaria hypoxylon
GL45937_S238
Leotia lubrica
Mollisia cinerea
Botryotinia fuckeliana
Monilinia fructicola
GL40305_S043
Cudoniella clavus
Dermea acerina
Coccomyces dentatus
Phacidiopycnis pyri
Chlorociboria aeruginosa
Amb_18S_1472_EF023999_Lesaulnier
GL45252_S163
GL37478_S201
clade GS35
DU60_KM096534_Fomina
GL39522_S107
Lachnum virgineum
Geoglossum nigritum
Trichoglossum hirsutum
Arthonia dispersa
Dendrographa minor
Simonyella variegata
Saccharomyces cerevisiae
Saccharomyces castellii
Kluyveromyces waltii
Ashbya gossypii
Kluyveromyces lactis
Candida glabrata
Candida tropicalis
Candida albicans
Candida guilliermondii
Debaryomyces hansenii
GL29348_G2700
GL23681_S006
GL11379_S006
GL31671_S045
GL26545_S046
clade GS31
N17_EU179934_Porter
Archaeorhizomyces borealis
Archaeorhizomyces finlayi
Neolecta vitellina
Neolecta irregularis
Schizosaccharomyces pombe
Protomyces inouyei
Taphrina wiesneri
Pneumocystis carinii
Sordariomycetes
Helotiales
Geoglossomycetes
Arthoniomycetes
Ascomycota: Pezizomycotina
Ascomycota: Saccharo-mycotina
Archaeorhizomycetes
Ascomycota: Taphrinomycotina
To: Fungi root

Figure S2.2

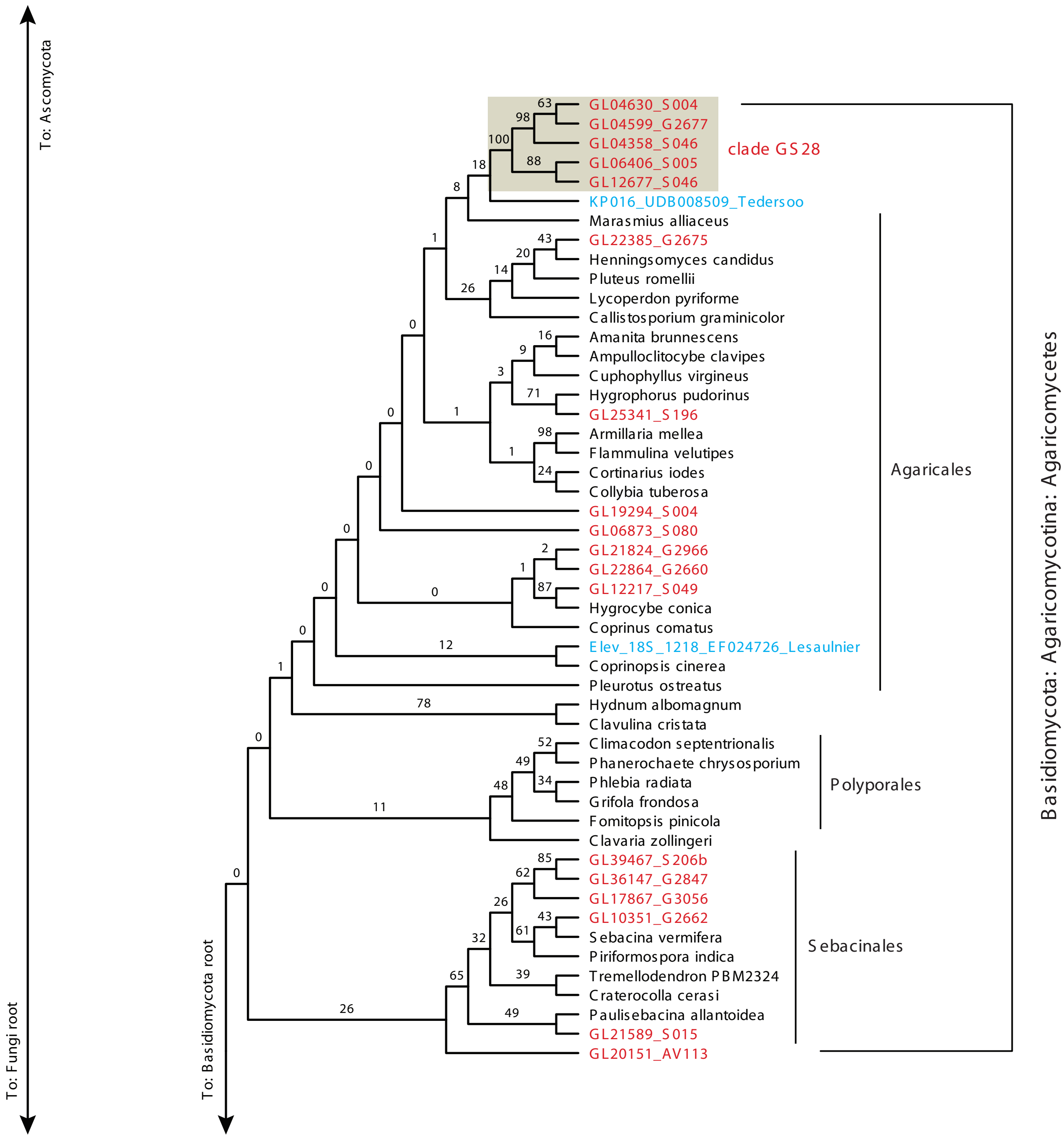

To: Ascomycota
To: Fungi root
To: Basidiomycota root
GL04630_S004
GL04599_G2677
GL04358_S046
GL06406_S005
GL12677_S046
clade GS28
KP016_UDB008509_Tedersoo
Marasmius alliaceus
GL22385_G2675
Henningsomyces candidus
Pluteus romellii
Lycoperdon pyriforme
Callistosporium graminicolor
Amanita brunnescens
Ampulloclitocybe clavipes
Cuphophyllus virgineus
Hygrophorus pudorinus
GL25341_S196
Armillaria mellea
Flammulina velutipes
Cortinarius iodes
Collybia tuberosa
GL19294_S004
GL06873_S080
GL21824_G2966
GL22864_G2660
GL12217_S049
Hygrocybe conica
Coprinus comatus
Elev_18S_1218_EF024726_Lesaulnier
Coprinopsis cinerea
Pleurotus ostreatus
Hydnum albomagnum
Clavulina cristata
Climacodon septentrionalis
Phanerochaete chrysosporium
Phlebia radiata
Grifola frondosa
Fomitopsis pinicola
Clavaria zollingeri
GL39467_S206b
GL36147_G2847
GL17867_G3056
GL10351_G2662
Sebacina vermifera
Piriformospora indica
Tremellodendron PBM2324
Craterocolla cerasi
Paulisebacina allantoidea
GL21589_S015
GL20151_AV113
Agaricales
Polyporales
Sebacinales
Basidiomycota: Agaricomycotina: Agaricomycetes

Figure S2.3

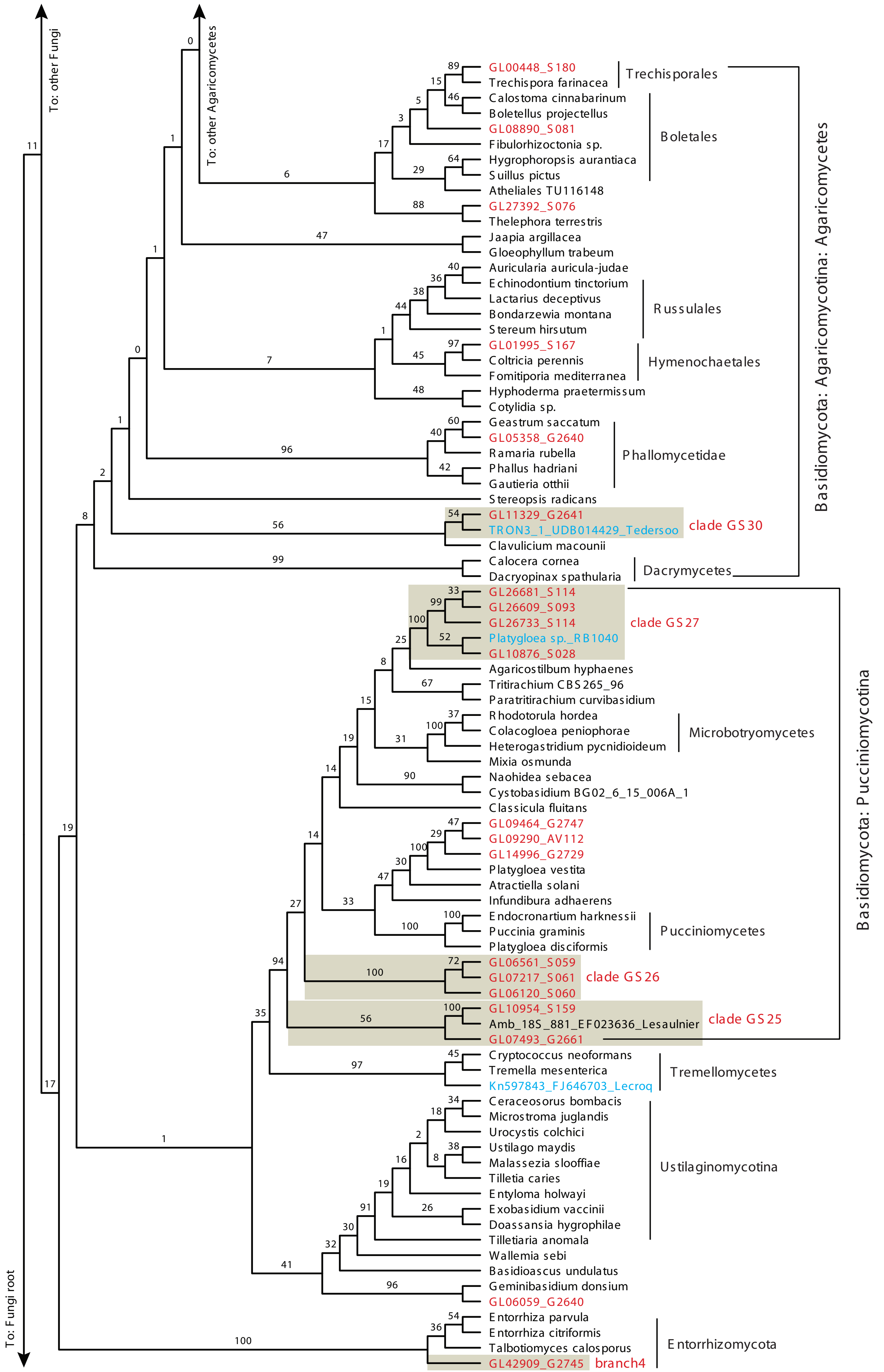
To: other Fungi
To: other Agaricomycetes
To: Fungi root
GL00448_S180
Trechispora farinacea
Trechisporales
Calostoma cinnabarinum
Boletellus projectellus
GL08890_S081
Fibulorhizoctonia sp.
Hygrophoropsis aurantiaca
Suillus pictus
Boletales
Atheliales TU116148
GL27392_S076
Thelephora terrestris
Jaapia argillacea
Gloeophyllum trabeum
Auricularia auricula-judae
Echinodontium tinctorium
Lactarius deceptivus
Bondarzewia montana
Stereum hirsutum
Russulales
GL01995_S167
Coltricia perennis
Fomitiporia mediterranea
Hymenochaetales
Hyphoderma praetermissum
Cotylidia sp.
Geastrum saccatum
GL05358_G2640
Ramaria rubella
Phallus hadriani
Gautieria otthii
Phallomycetidae
Stereopsis radicans
GL11329_G2641
TRON3_1_UDB014429_Tedersoo
clade GS30
Clavulicium macounii
Calocera cornea
Dacryopinax spathularia
Dacrymycetes
Basidiomycota: Agaricomycotina: Agaricomycetes
GL26681_S114
GL26609_S093
GL26733_S114
Platygloea sp._RB1040
GL10876_S028
clade GS27
Agaricostilbum hyphaenes
Tritirachium CBS265_96
Paratritirachium curvibasidium
Rhodotorula hordea
Colacogloea peniophorae
Heterogastridium pycnidioideum
Microbotryomycetes
Mixia osmunda
Naohidea sebacea
Cystobasidium BG02_6_15_006A_1
Classicula fluitans
GL09464_G2747
GL09290_AV112
GL14996_G2729
Platygloea vestita
Atractiella solani
Infundibura adhaerens
Endocronartium harknessii
Puccinia graminis
Platygloea disciformis
Pucciniomycetes
GL06561_S059
GL07217_S061
GL06120_S060
clade GS26
GL10954_S159
Amb_18S_881_EF023636_Lesaulnier
GL07493_G2661
clade GS25
Basidiomycota: Pucciniomycotina
Cryptococcus neoformans
Tremella mesenterica
Kn597843_FJ646703_Lecroq
Tremellomycetes
Ceraceosorus bombacis
Microstroma juglandis
Urocystis colchici
Ustilago maydis
Malassezia slooffiae
Tilletia caries
Entyloma holwayi
Exobasidium vaccinii
Doassansia hygrophilae
Ustilaginomycotina
Tilletiaria anomala
Wallemia sebi
Basidioascus undulatus
Geminibasidium donsium
GL06059_G2640
Entorrhiza parvula
Entorrhiza citriformis
Talbotiomyces calosporus
GL42909_G2745
branch4
Entorrhizomycota

Figure S2.4

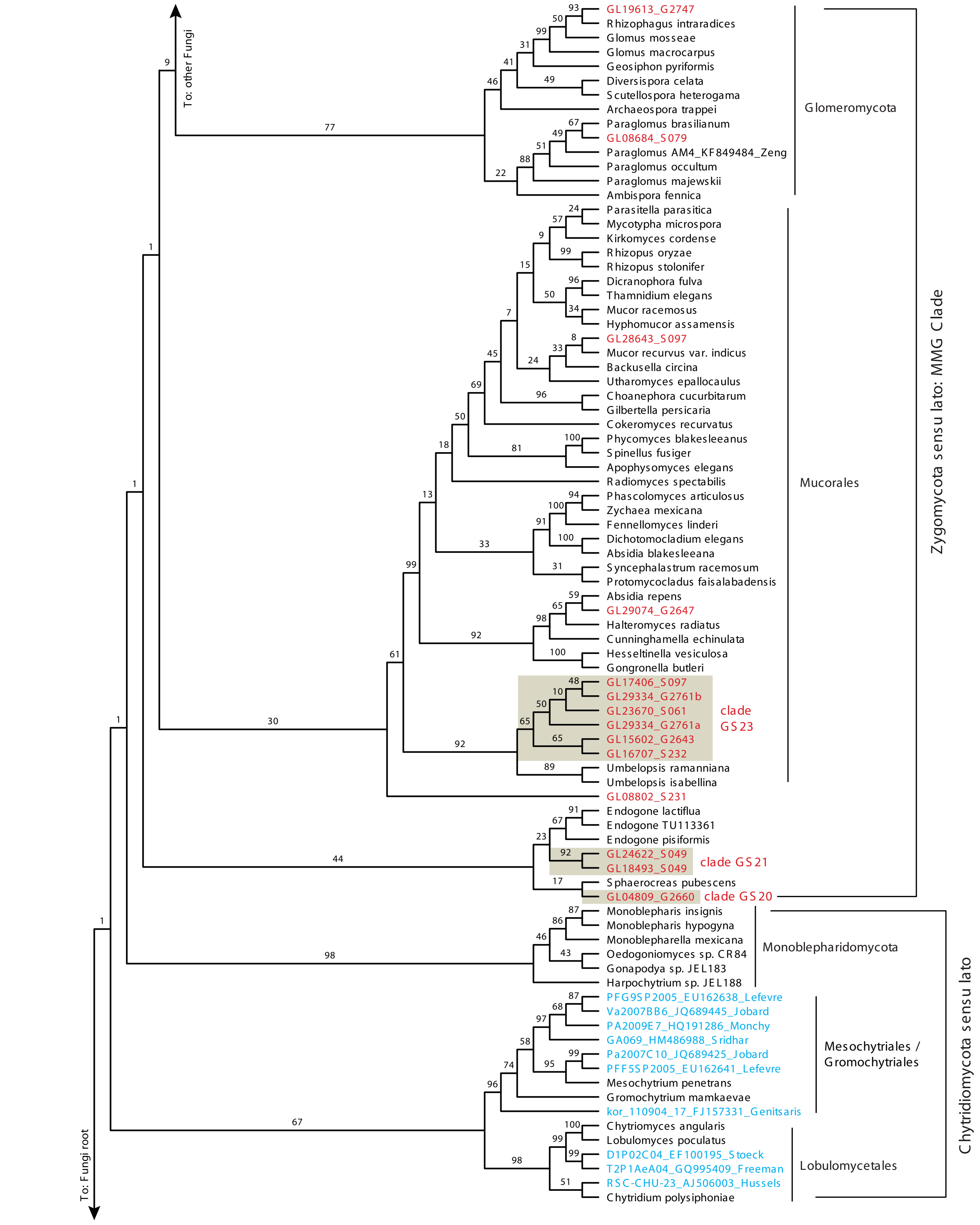

To: other Fungi
GL19613_G2747
Rhizophagus intraradices
Glomus mosseae
Glomus macrocarpus
Geosiphon pyriformis
Diversispora celata
Scutellospora heterogama
Archaeospora trappei
Paraglomus brasilianum
GL08684_S079
Paraglomus AM4_KF849484_Zeng
Paraglomus occultum
Paraglomus majewskii
Ambispora fennica
Glomeromycota
Parasitella parasitica
Mycotypha microspora
Kirkomyces cordense
Rhizopus oryzae
Rhizopus stolonifer
Dicranophora fulva
Thamnidium elegans
Mucor racemosus
Hyphomucor assamensis
GL28643_S097
Mucor recurvus var. indicus
Backusella circina
Utharomyces epallocaulus
Choanephora cucurbitarum
Gilbertella persicaria
Cokeromyces recurvatus
Phycomyces blakesleeanus
Spinellus fusiger
Apophysomyces elegans
Radiomyces spectabilis
Phascolomyces articulosus
Zychaea mexicana
Fennellomyces linderi
Dichotomocladium elegans
Absidia blakesleeana
Syncephalastrum racemosum
Protomycocladus faisalabadensis
Absidia repens
GL29074_G2647
Halteromyces radiatus
Cunninghamella echinulata
Hesseltinella vesiculosa
Gongronella butleri
GL17406_S097
GL29334_G2761b
GL23670_S061
GL29334_G2761a
GL15602_G2643
GL16707_S232
clade GS23
Umbelopsis ramanniana
Umbelopsis isabellina
Mucorales
GL08802_S231
Endogone lactiflua
Endogone TU113361
Endogone pisiformis
GL24622_S049
GL18493_S049
clade GS21
Sphaerocreas pubescens
GL04809_G2660
clade GS20
Zygomycota sensu lato: MMG Clade
Monoblepharis insignis
Monoblepharis hypogyna
Monoblepharella mexicana
Oedogoniomyces sp. CR84
Gonapodya sp. JEL183
Harpochytrium sp. JEL188
Monoblepharidomycota
PFG9SP2005_EU162638_Lefevre
Va2007BB6_JQ689445_Jobard
PA2009E7_HQ191286_Monchy
GA069_HM486988_Sridhar
Pa2007C10_JQ689425_Jobard
PFF5SP2005_EU162641_Lefevre
Mesochytrium penetrans
Gromochytrium mamkaevae
kor_110904_17_FJ157331_Genitsaris
Mesochytriales / Gromochytriales
Chytriomyces angularis
Lobulomyces poculatus
D1P02C04_EF100195_Stoeck
T2P1AeA04_GQ995409_Freeman
RSC-CHU-23_AJ506003_Hussels
Chytridium polysiphoniae
Lobulomycetales
Chytridiomycota sensu lato
To: Fungi root

Figure S2.5

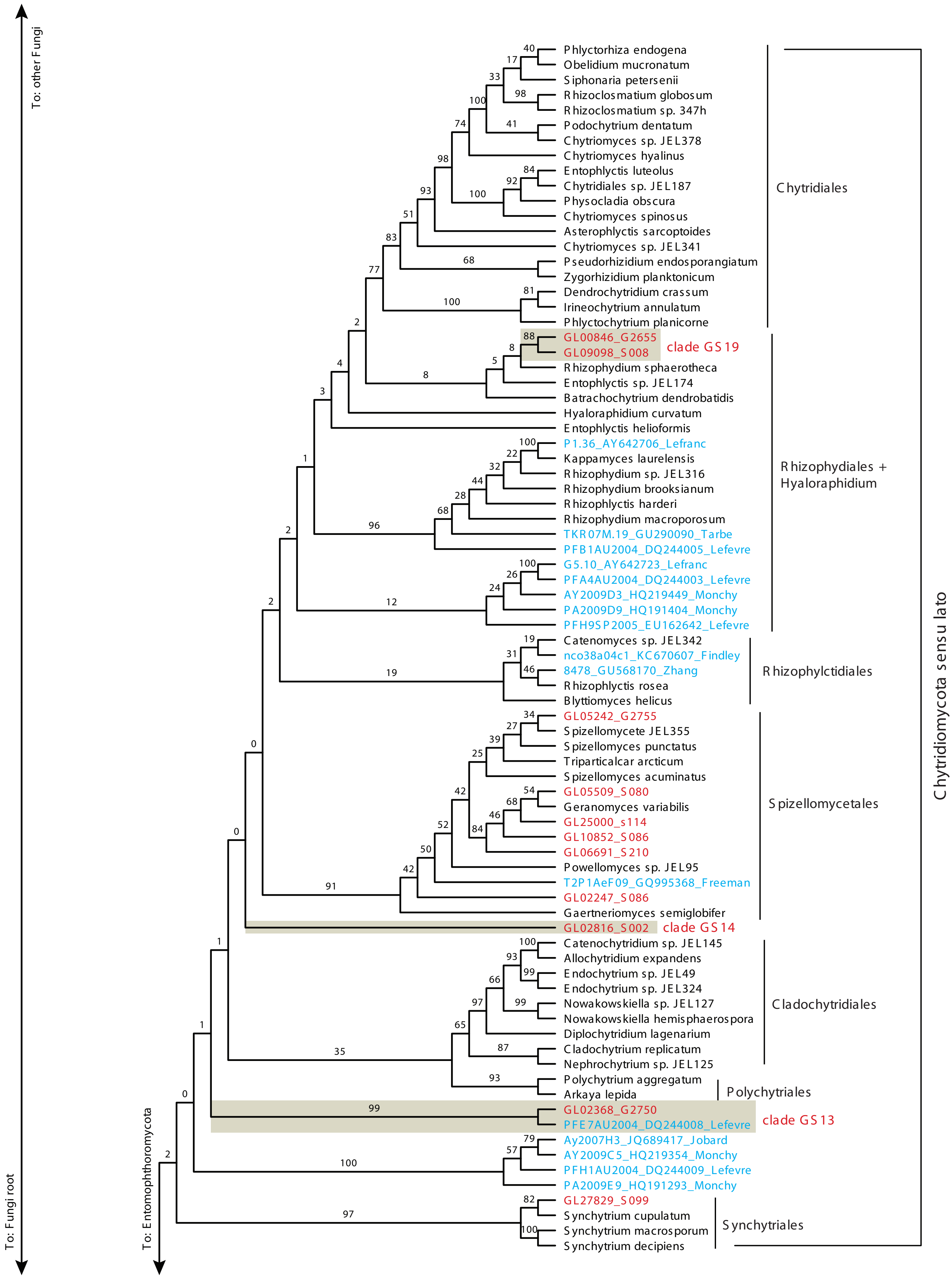
To: other Fungi
To: Fungi root
To: Entomophthoromycota
Phlyctorhiza endogena
Obelidium mucronatum
Siphonaria petersenii
Rhizoclosmatium globosum
Rhizoclosmatium sp. 347h
Podochytrium dentatum
Chytriomyces sp. JEL378
Chytriomyces hyalinus
Entophlyctis luteolus
Chytridiales sp. JEL187
Physocladia obscura
Chytriomyces spinosus
Asterophlyctis sarcoptoides
Chytriomyces sp. JEL341
Pseudorhizidium endosporangiatum
Zygorhizidium planktonicum
Dendrochytridium crassum
Irineochytrium annulatum
Phlyctochytrium planicorne
GL00846_G2655
GL09098_S008
Rhizophydium sphaerotheca
Entophlyctis sp. JEL174
Batrachochytrium dendrobatidis
Hyaloraphidium curvatum
Entophlyctis helioformis
P1.36_AY642706_Lefranc
Kappamyces laurelensis
Rhizophydium sp. JEL316
Rhizophydium brooksianum
Rhizophlyctis harderi
Rhizophydium macroporosum
TKR07M.19_GU290090_Tarbe
PFB1AU2004_DQ244005_Lefevre
G5.10_AY642723_Lefranc
PFA4AU2004_DQ244003_Lefevre
AY2009D3_HQ219449_Monchy
PA2009D9_HQ191404_Monchy
PFH9SP2005_EU162642_Lefevre
Catenomyces sp. JEL342
nco38a04c1_KC670607_Findley
8478_GU568170_Zhang
Rhizophlyctis rosea
Blyttiomyces helicus
GL05242_G2755
Spizellomycete JEL355
Spizellomyces punctatus
Triparticalcar arcticum
Spizellomyces acuminatus
GL05509_S080
Geranomyces variabilis
GL25000_s114
GL10852_S086
GL06691_S210
Powellomyces sp. JEL95
T2P1AeF09_GQ995368_Freeman
GL02247_S086
Gaertneriomyces semiglobifer
GL02816_S002
Catenochytridium sp. JEL145
Allochytridium expandens
Endochytrium sp. JEL49
Endochytrium sp. JEL324
Nowakowskiella sp. JEL127
Nowakowskiella hemisphaerospora
Diplochytridium lagenarium
Cladochytrium replicatum
Nephrochytrium sp. JEL125
Polychytrium aggregatum
Arkaya lepida
GL02368_G2750
PFE7AU2004_DQ244008_Lefevre
Ay2007H3_JQ689417_Jobard
AY2009C5_HQ219354_Monchy
PFH1AU2004_DQ244009_Lefevre
PA2009E9_HQ191293_Monchy
GL27829_S099
Synchytrium cupulatum
Synchytrium macrosporum
Synchytrium decipiens
Chytridiales
clade GS19
Rhizophydiales +
Hyaloraphidium
Rhizophylctidiales
Spizellomycetales
clade GS14
Cladochytridiales
Polychytriales
clade GS13
Synchytriales
Chytridiomycota sensu lato

Figure S2.6

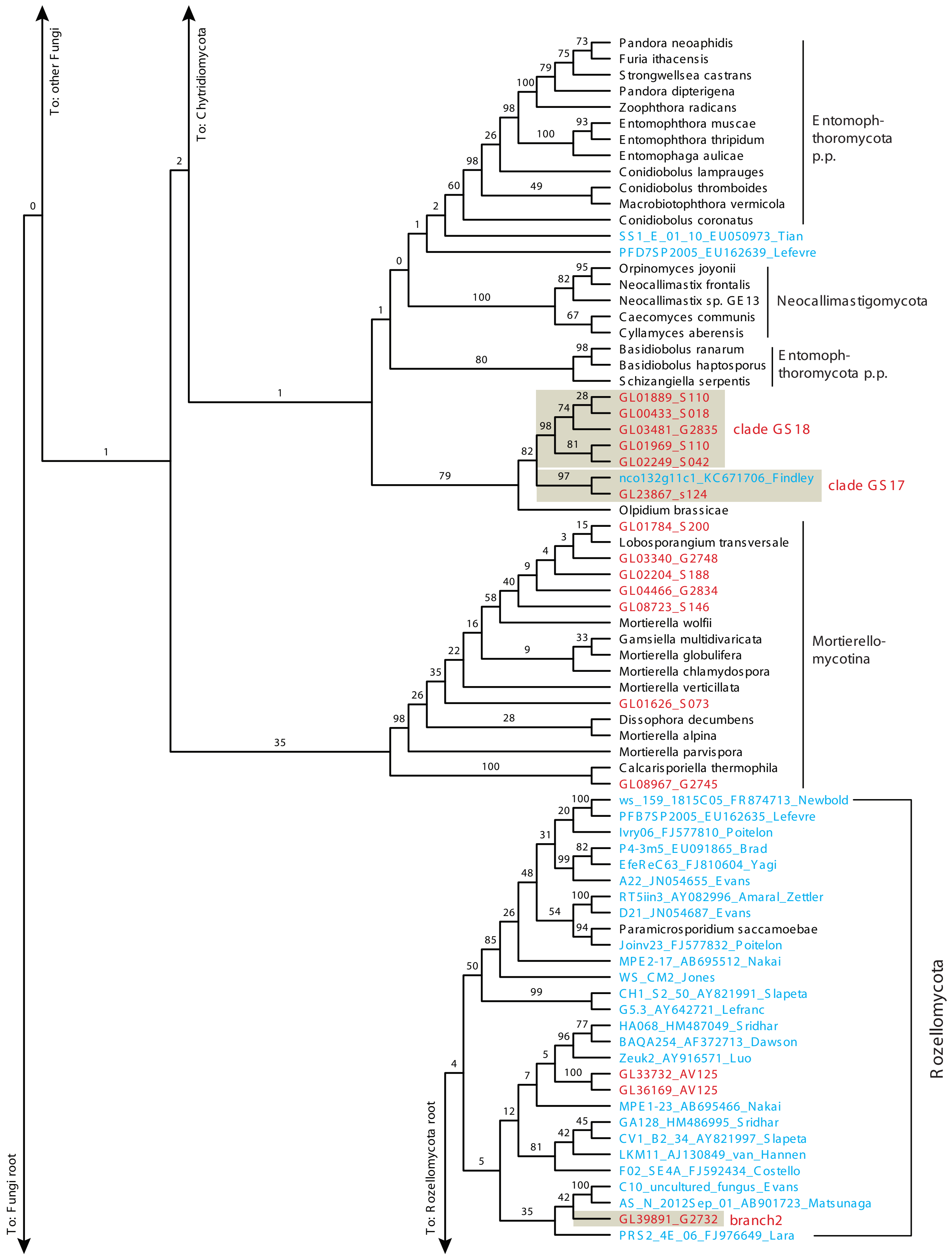

To: other Fungi
To: Chytridiomycota
To: Fungi root
To: Rozellomycota root
Pandora neoaphidis
Furia ithacensis
Strongwellsea castrans
Pandora dipterigena
Zoophthora radicans
Entomophthora muscae
Entomophthora thripidum
Entomophaga aulicae
Conidiobolus lamprauges
Conidiobolus thromboides
Macrobiotophthora vermicola
Conidiobolus coronatus
Entomoph-thoromycota p.p.
SS1_E_01_10_EU050973_Tian
PFD7SP2005_EU162639_Lefevre
Orpinomyces joyonii
Neocallimastix frontalis
Neocallimastix sp. GE13
Caecomyces communis
Cyllamyces aberensis
Neocallimastigomycota
Basidiobolus ranarum
Basidiobolus haptosporus
Schizangiella serpentis
Entomoph-thoromycota p.p.
GL01889_S110
GL00433_S018
GL03481_G2835
GL01969_S110
GL02249_S042
clade GS18
nco132g11c1_KC671706_Findley
GL23867_s124
clade GS17
Olpidium brassicae
GL01784_S200
Lobosporangium transversale
GL03340_G2748
GL02204_S188
GL04466_G2834
GL08723_S146
Mortierella wolfii
Gamsiella multidivaricata
Mortierella globulifera
Mortierella chlamydospora
Mortierella verticillata
GL01626_S073
Dissophora decumbens
Mortierella alpina
Mortierella parvispora
Calcarisporiella thermophila
GL08967_G2745
Mortierello-mycotina
ws_159_1815C05_FR874713_Newbold
PFB7SP2005_EU162635_Lefevre
Ivry06_FJ577810_Poitelon
P4-3m5_EU091865_Brad
EfeReC63_FJ810604_Yagi
A22_JN054655_Evans
RT5iin3_AY082996_Amaral_Zettler
D21_JN054687_Evans
Paramicrosporidium saccamoebae
Joinv23_FJ577832_Poitelon
MPE2-17_AB695512_Nakai
WS_CM2_Jones
CH1_S2_50_AY821991_Slapeta
G5.3_AY642721_Lefranc
HA068_HM487049_Sridhar
BAQA254_AF372713_Dawson
Zeuk2_AY916571_Luo
GL33732_AV125
GL36169_AV125
MPE1-23_AB695466_Nakai
GA128_HM486995_Sridhar
CV1_B2_34_AY821997_Slapeta
LKM11_AJ130849_van_Hannen
F02_SE4A_FJ592434_Costello
C10_uncultured_fungus_Evans
AS_N_2012Sep_01_AB901723_Matsunaga
GL39891_G2732
branch2
PRS2_4E_06_FJ976649_Lara
Rozellomycota

Figure S2.7

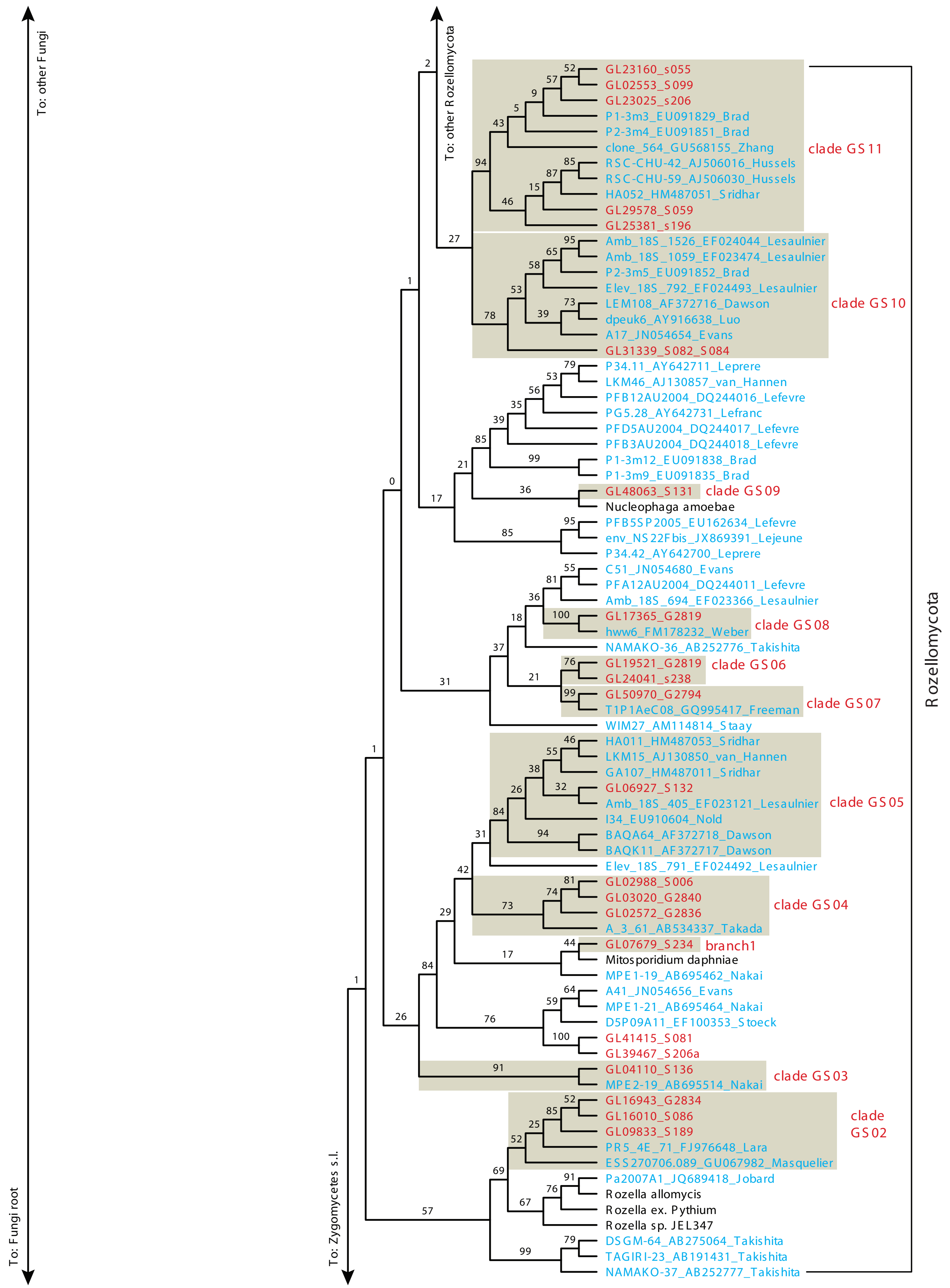

To: other Fungi
To: Fungi root
To: other Rozellomycota
To: Zygomycetes s.l.
GL23160_s055
GL02553_S099
GL23025_s206
P1-3m3_EU091829_Brad
P2-3m4_EU091851_Brad
clone_564_GU568155_Zhang
RSC-CHU-42_AJ506016_Hussels
RSC-CHU-59_AJ506030_Hussels
HA052_HM487051_Sridhar
GL29578_S059
GL25381_s196
clade GS11
Amb_18S_1526_EF024044_Lesaulnier
Amb_18S_1059_EF023474_Lesaulnier
P2-3m5_EU091852_Brad
Elev_18S_792_EF024493_Lesaulnier
LEM108_AF372716_Dawson
dpeuk6_AY916638_Luo
A17_JN054654_Evans
GL31339_S082_S084
clade GS10
P34.11_AY642711_Leprere
LKM46_AJ130857_van_Hannen
PFB12AU2004_DQ244016_Lefevre
PG5.28_AY642731_Lefranc
PFD5AU2004_DQ244017_Lefevre
PFB3AU2004_DQ244018_Lefevre
P1-3m12_EU091838_Brad
P1-3m9_EU091835_Brad
GL48063_S131
clade GS09
Nucleophaga amoebae
PFB5SP2005_EU162634_Lefevre
env_NS22Fbis_JX869391_Lejeune
P34.42_AY642700_Leprere
C51_JN054680_Evans
PFA12AU2004_DQ244011_Lefevre
Amb_18S_694_EF023366_Lesaulnier
GL17365_G2819
hww6_FM178232_Weber
clade GS08
NAMAKO-36_AB252776_Takishita
GL19521_G2819
GL24041_s238
clade GS06
GL50970_G2794
T1P1AeC08_GQ995417_Freeman
clade GS07
WIM27_AM114814_Staay
HA011_HM487053_Sridhar
LKM15_AJ130850_van_Hannen
GA107_HM487011_Sridhar
GL06927_S132
Amb_18S_405_EF023121_Lesaulnier
I34_EU910604_Nold
BAQA64_AF372718_Dawson
BAQK11_AF372717_Dawson
clade GS05
Elev_18S_791_EF024492_Lesaulnier
GL02988_S006
GL03020_G2840
GL02572_G2836
A_3_61_AB534337_Takada
clade GS04
GL07679_S234
branch1
Mitosporidium daphniae
MPE1-19_AB695462_Nakai
A41_JN054656_Evans
MPE1-21_AB695464_Nakai
D5P09A11_EF100353_Stoeck
GL41415_S081
GL39467_S206a
GL04110_S136
MPE2-19_AB695514_Nakai
clade GS03
GL16943_G2834
GL16010_S086
GL09833_S189
PR5_4E_71_FJ976648_Lara
ESS270706.089_GU067982_Masquelier
clade GS02
Pa2007A1_JQ689418_Jobard
Rozella allomycis
Rozella ex. Pythium
Rozella sp. JEL347
DSGM-64_AB275064_Takishita
TAGIRI-23_AB191431_Takishita
NAMAKO-37_AB252777_Takishita
Rozellomycota

Figure S2.8

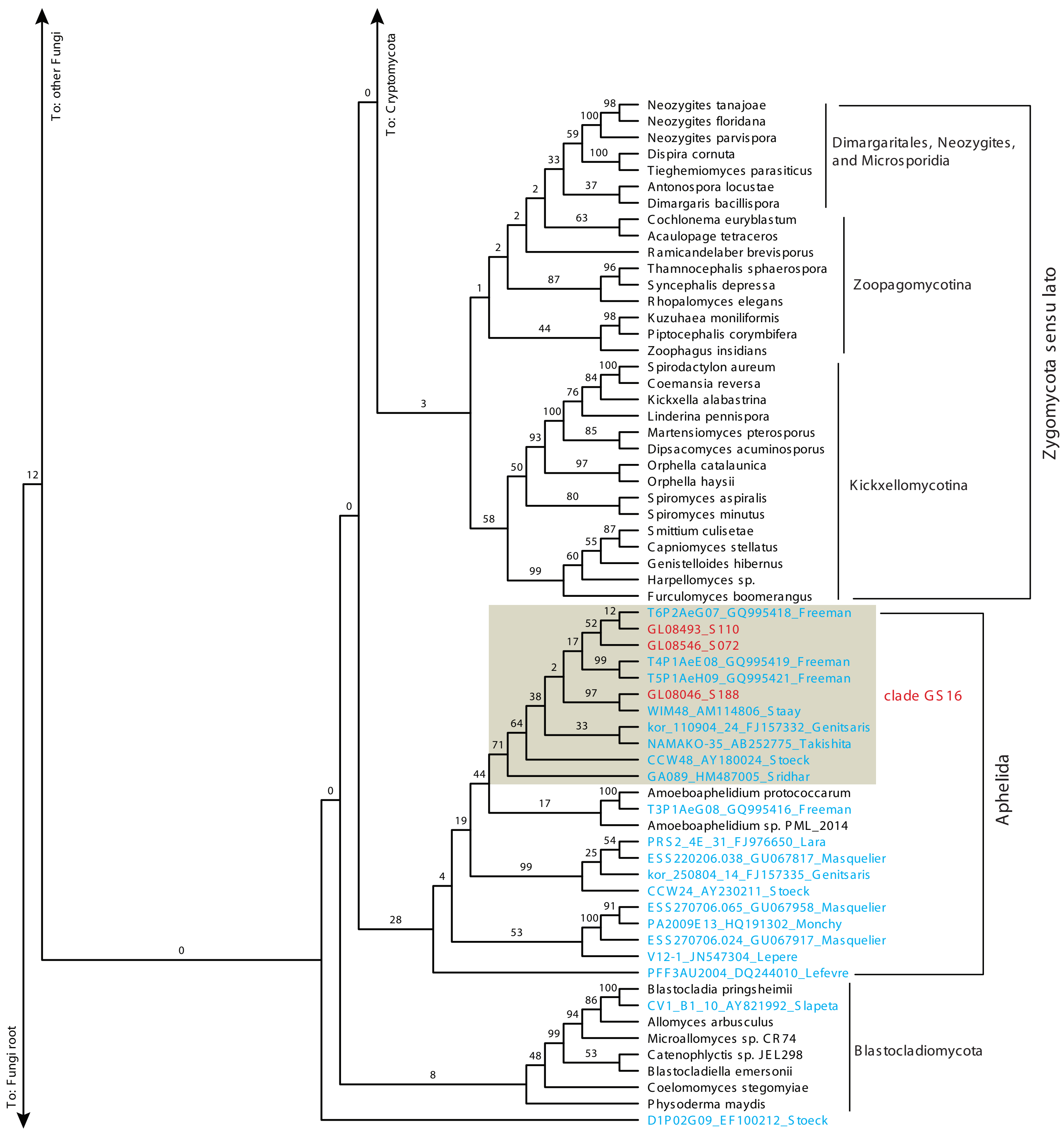
To: other Fungi
To: Cryptomycota
To: Fungi root
Neozygites tanajoae
Neozygites floridana
Neozygites parvispora
Dispira cornuta
Tieghemiomyces parasiticus
Antonospora locustae
Dimargaris bacillispora
Cochlonema euryblastum
Acaulopage tetraceros
Ramicandelaber brevisporus
Thamnocephalis sphaerospora
Syncephalis depressa
Rhopalomyces elegans
Kuzuhaea moniliformis
Piptocephalis corymbifera
Zoophagus insidians
Spirodactylon aureum
Coemansia reversa
Kickxella alabastrina
Linderina pennispora
Martensiomyces pterosporus
Dipsacomyces acuminosporus
Orphella catalaunica
Orphella haysii
Spiromyces aspiralis
Spiromyces minutus
Smittium culisetae
Capniomyces stellatus
Genistelloides hibernus
Harpellomyces sp.
Furculomyces boomerangus
T6P2AeG07_GQ995418_Freeman
GL08493_S110
GL08546_S072
T4P1AeE08_GQ995419_Freeman
T5P1AeH09_GQ995421_Freeman
GL08046_S188
WIM48_AM114806_Staay
kor_110904_24_FJ157332_Genitsaris
NAMAKO-35_AB252775_Takishita
CCW48_AY180024_Stoeck
GA089_HM487005_Sridhar
Amoeboaphelidium protococcarum
T3P1AeG08_GQ995416_Freeman
Amoeboaphelidium sp. PML_2014
PRS2_4E_31_FJ976650_Lara
ESS220206.038_GU067817_Masquelier
kor_250804_14_FJ157335_Genitsaris
CCW24_AY230211_Stoeck
ESS270706.065_GU067958_Masquelier
PA2009E13_HQ191302_Monchy
ESS270706.024_GU067917_Masquelier
V12-1_JN547304_Lepere
PFF3AU2004_DQ244010_Lefevre
Blastocladia pringsheimii
CV1_B1_10_AY821992_Slapeta
Allomyces arbusculus
Microallomyces sp. CR74
Catenophlyctis sp. JEL298
Blastocladiella emersonii
Coelomomyces stegomyiae
Physoderma maydis
D1P02G09_EF100212_Stoeck
Dimargaritales, Neozygites, and Microsporidia
Zoopagomycotina
Kickxellomycotina
Zygomycota sensu lato
clade GS16
Aphelida
Blastocladiomycota

Figure S2.9

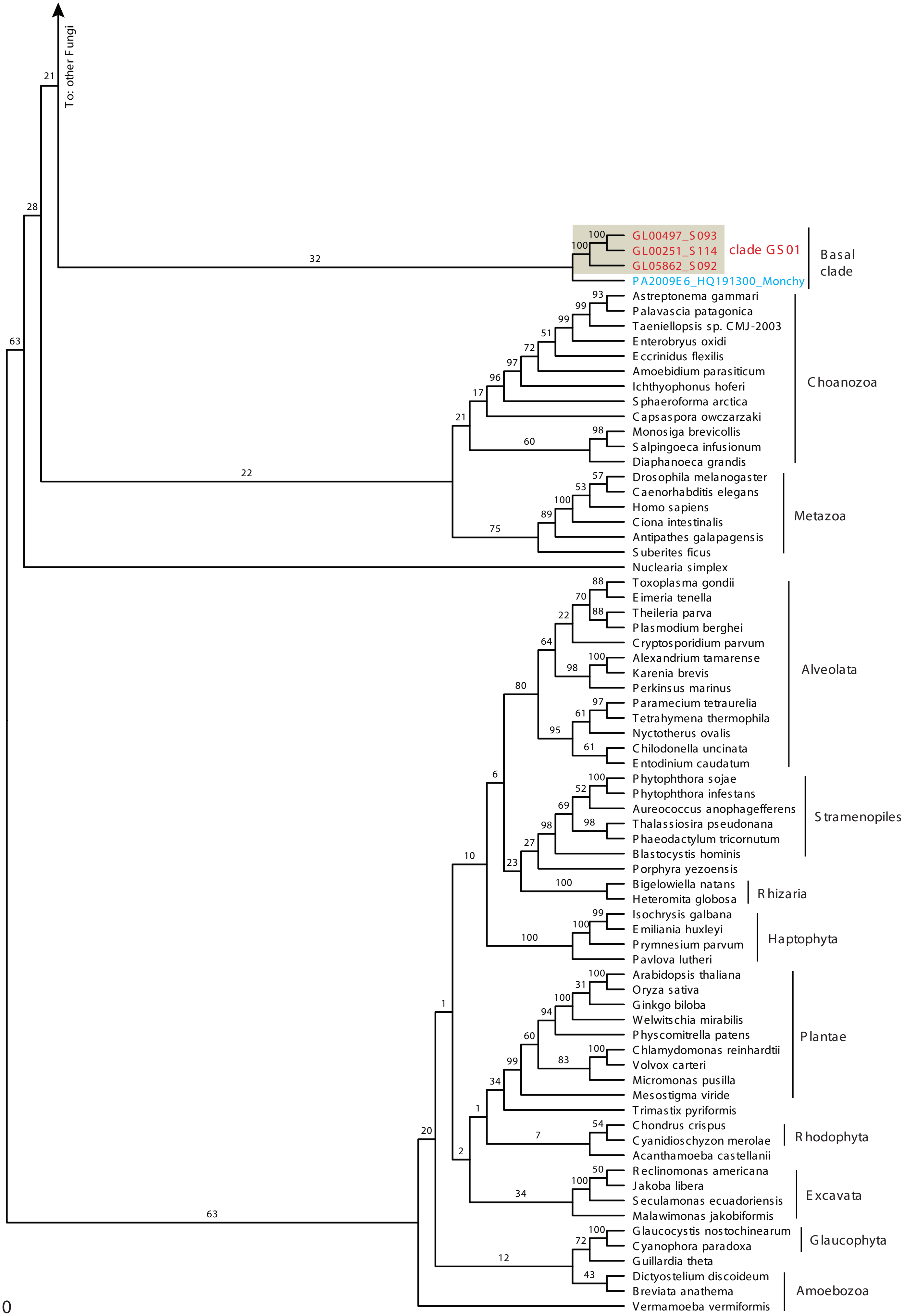
To: other Fungi
GL00497_S093
GL00251_S114
GL05862_S092
clade GS01
PA2009E6_HQ191300_Monchy
Basal clade
Astreptonema gammari
Palavascia patagonica
Taeniellopsis sp. CMJ-2003
Enterobryus oxidi
Eccrinidus flexilis
Amoebidium parasiticum
Ichthyophonus hoferi
Sphaeroforma arctica
Capsaspora owczarzaki
Monosiga brevicollis
Salpingoeca infusionum
Diaphanoeca grandis
Choanozoa
Drosophila melanogaster
Caenorhabditis elegans
Homo sapiens
Ciona intestinalis
Antipathes galapagensis
Suberites ficus
Metazoa
Nuclearia simplex
Toxoplasma gondii
Eimeria tenella
Theileria parva
Plasmodium berghei
Cryptosporidium parvum
Alexandrium tamarense
Karenia brevis
Perkinsus marinus
Paramecium tetraurelia
Tetrahymena thermophila
Nyctotherus ovalis
Chilodonella uncinata
Entodinium caudatum
Alveolata
Phytophthora sojae
Phytophthora infestans
Aureococcus anophagefferens
Thalassiosira pseudonana
Phaeodactylum tricornutum
Blastocystis hominis
Stramenopiles
Porphyra yezoensis
Bigelowiella natans
Heteromita globosa
Rhizaria
Isochrysis galbana
Emiliania huxleyi
Prymnesium parvum
Pavlova lutheri
Haptophyta
Arabidopsis thaliana
Oryza sativa
Ginkgo biloba
Welwitschia mirabilis
Physcomitrella patens
Chlamydomonas reinhardtii
Volvox carteri
Micromonas pusilla
Mesostigma viride
Plantae
Trimastix pyriformis
Chondrus crispus
Cyanidioschyzon merolae
Rhodophyta
Acanthamoeba castellanii
Reclinomonas americana
Jakoba libera
Seculamonas ecuadoriensis
Malawimonas jakobiformis
Excavata
Glaucocystis nostochinearum
Cyanophora paradoxa
Glaucophyta
Guillardia theta
Dictyostelium discoideum
Breviata anathema
Vermamoeba vermiformis
Amoebozoa

Figure S2.10

Figure S3. Phylogenetic placement of soil-inhabiting fungi among identified taxa and other sequences from the environment based on a full 28S rRNA gene Maximum Likelihood phylogram. Red, blue, and black fonts depict groups recovered here, in other environmental sequencing studies, and based on vouchered collections, respectively. Shaded clusters indicate prominent clades and branches discussed.

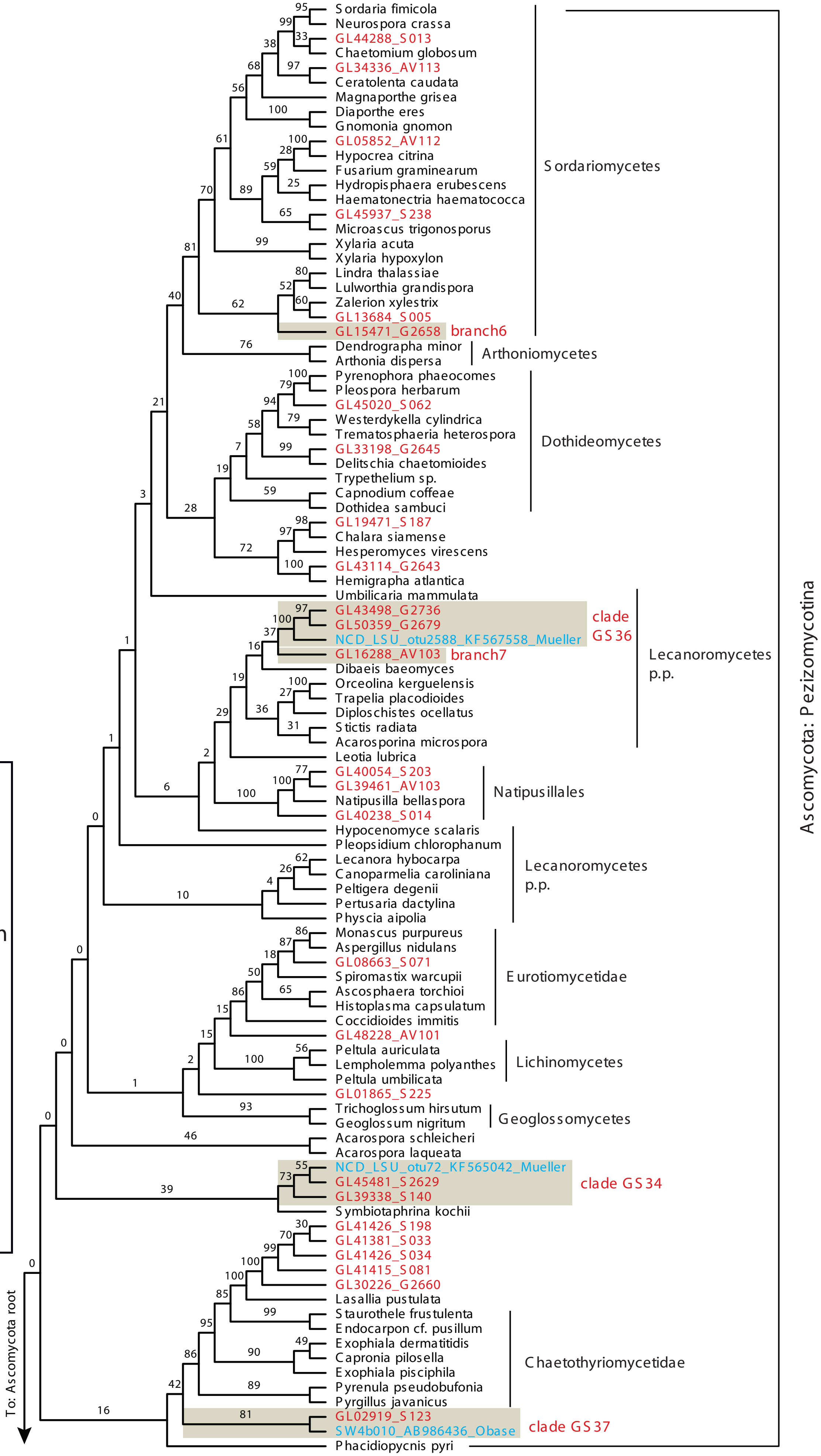

Figure S3.1

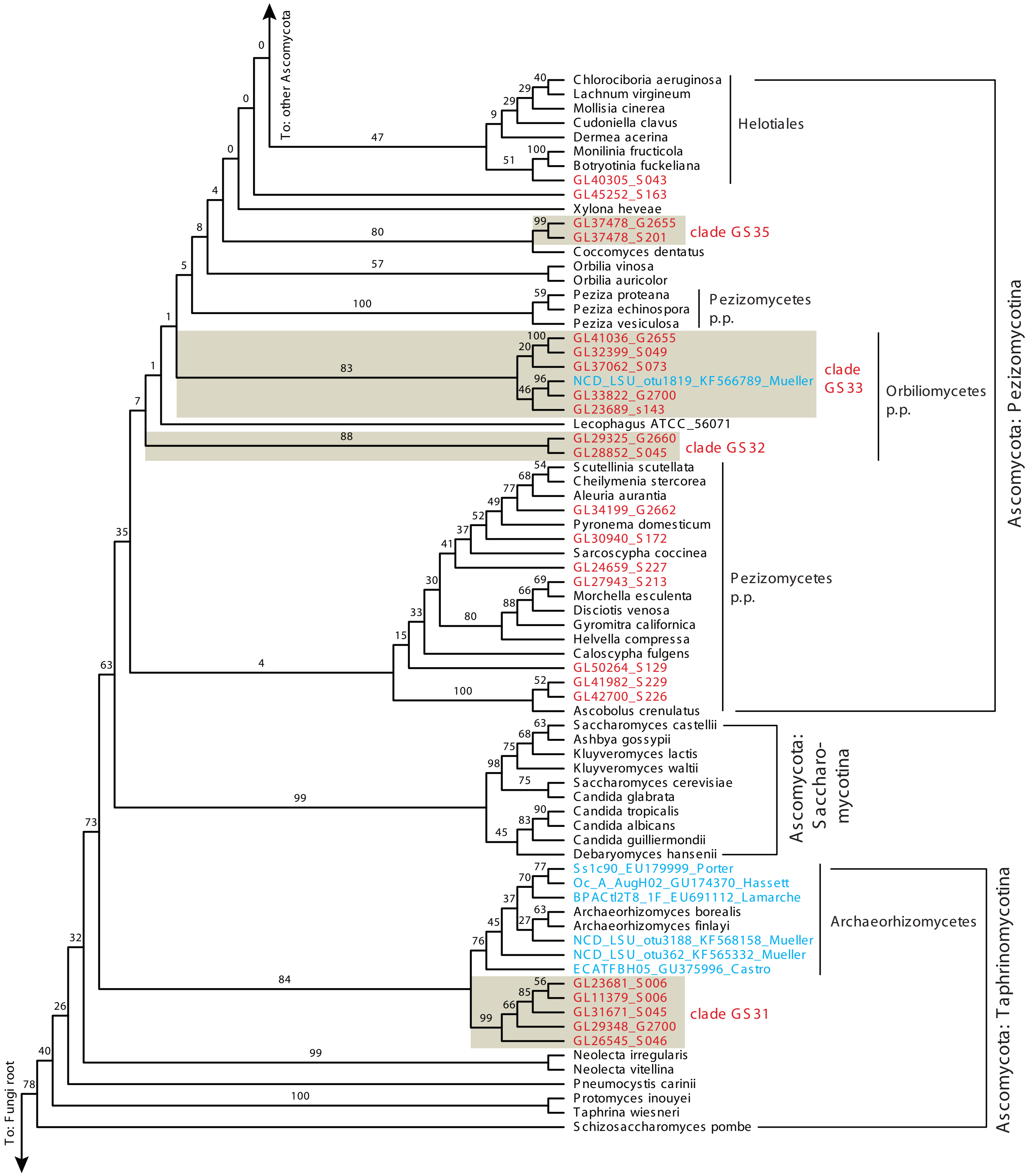
To: other Ascomycota
Chlorociboria aeruginosa
Lachnum virgineum
Mollisia cinerea
Cudoniella clavus
Dermea acerina
Monilinia fructicola
Botryotinia fuckeliana
GL40305_S043
GL45252_S163
Xylona heveae
GL37478_G2655
GL37478_S201
Coccomyces dentatus
Orbilia vinosa
Orbilia auricolor
Peziza proteana
Peziza echinospora
Peziza vesiculosa
GL41036_G2655
GL32399_S049
GL37062_S073
NCD_LSU_otu1819_KF566789_Mueller
GL33822_G2700
GL23689_s143
Lecophagus ATCC_56071
GL29325_G2660
GL28852_S045
Scutellinia scutellata
Cheilymenia stercorea
Aleuria aurantia
GL34199_G2662
Pyronema domesticum
GL30940_S172
Sarcoscypha coccinea
GL24659_S227
GL27943_S213
Morchella esculenta
Disciotis venosa
Gyromitra californica
Helvella compressa
Caloscypha fulgens
GL50264_S129
GL41982_S229
GL42700_S226
Ascobolus crenulatus
Saccharomyces castellii
Ashbya gossypii
Kluyveromyces lactis
Kluyveromyces waltii
Saccharomyces cerevisiae
Candida glabrata
Candida tropicalis
Candida albicans
Candida guilliermondii
Debaryomyces hansenii
Ss1c90_EU179999_Porter
Oc_A_AugH02_GU174370_Hassett
BPACtl2T8_1F_EU691112_Lamarche
Archaeorhizomyces borealis
Archaeorhizomyces finlayi
NCD_LSU_otu3188_KF568158_Mueller
NCD_LSU_otu362_KF565332_Mueller
ECATFBH05_GU375996_Castro
GL23681_S006
GL11379_S006
GL31671_S045
GL29348_G2700
GL26545_S046
Neolecta irregularis
Neolecta vitellina
Pneumocystis carinii
Protomyces inouyei
Taphrina wiesneri
Schizosaccharomyces pombe
To: Fungi root
Helotiales
clade GS35
Pezizomycetes p.p.
clade GS33
Orbiliomycetes p.p.
clade GS32
Pezizomycetes p.p.
Ascomycota: Pezizomycotina
Ascomycota: Saccharo-mycotina
Archaeorhizomycetes
clade GS31
Ascomycota: Taphrinomycotina

Figure S3.2

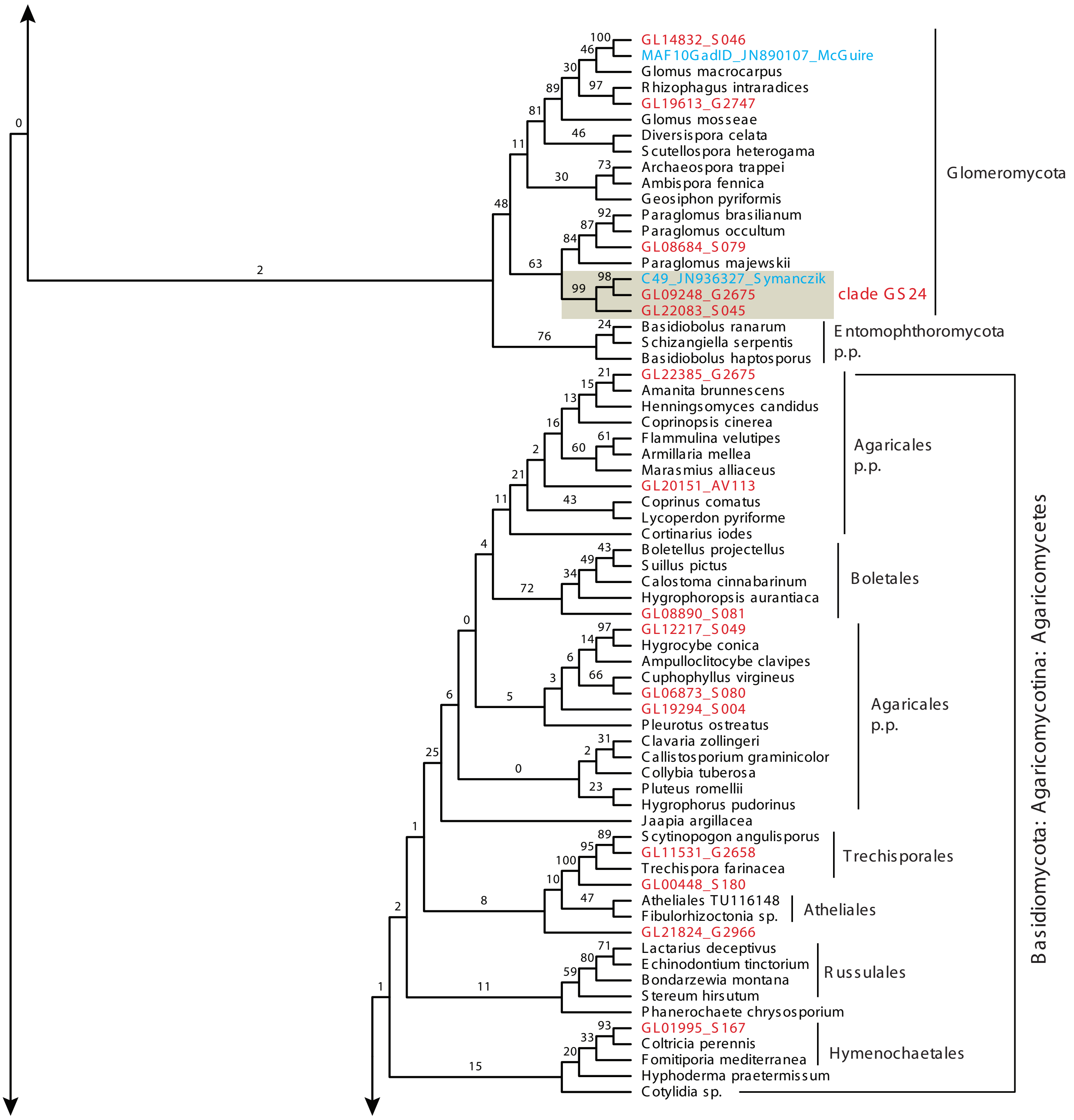

GL14832_S046
MAF10GadID_JN890107_McGuire
Glomus macrocarpus
Rhizophagus intraradices
GL19613_G2747
Glomus mosseae
Diversispora celata
Scutellospora heterogama
Archaeospora trappei
Ambispora fennica
Geosiphon pyriformis
Paraglomus brasilianum
Paraglomus occultum
GL08684_S079
Paraglomus majewskii
C49_JN936327_Symanczik
GL09248_G2675
GL22083_S045
clade GS24
Glomeromycota
Basidiobolus ranarum
Schizangiella serpentis
Basidiobolus haptosporus
Entomophthoromycota p.p.
GL22385_G2675
Amanita brunnescens
Henningsomyces candidus
Coprinopsis cinerea
Flammulina velutipes
Armillaria mellea
Marasmius alliaceus
GL20151_AV113
Coprinus comatus
Lycoperdon pyriforme
Cortinarius iodes
Agaricales p.p.
Boletellus projectellus
Suillus pictus
Calostoma cinnabarinum
Hygrophoropsis aurantiaca
GL08890_S081
Boletales
GL12217_S049
Hygrocybe conica
Ampulloclitocybe clavipes
Cuphophyllus virgineus
GL06873_S080
GL19294_S004
Pleurotus ostreatus
Clavaria zollingeri
Callistosporium graminicolor
Collybia tuberosa
Pluteus romellii
Hygrophorus pudorinus
Agaricales p.p.
Jaapia argillacea
Scytinopogon angulisporus
GL11531_G2658
Trechispora farinacea
GL00448_S180
Trechisporales
Atheliales TU116148
Fibulorhizoctonia sp.
Atheliales
GL21824_G2966
Lactarius deceptivus
Echinodontium tinctorium
Bondarzewia montana
Stereum hirsutum
Russulales
Phanerochaete chrysosporium
GL01995_S167
Coltricia perennis
Fomitiporia mediterranea
Hymenochaetales
Hyphoderma praetermissum
Cotylidia sp.
Basidiomycota: Agaricomycotina: Agaricomycetes

Figure S3.3

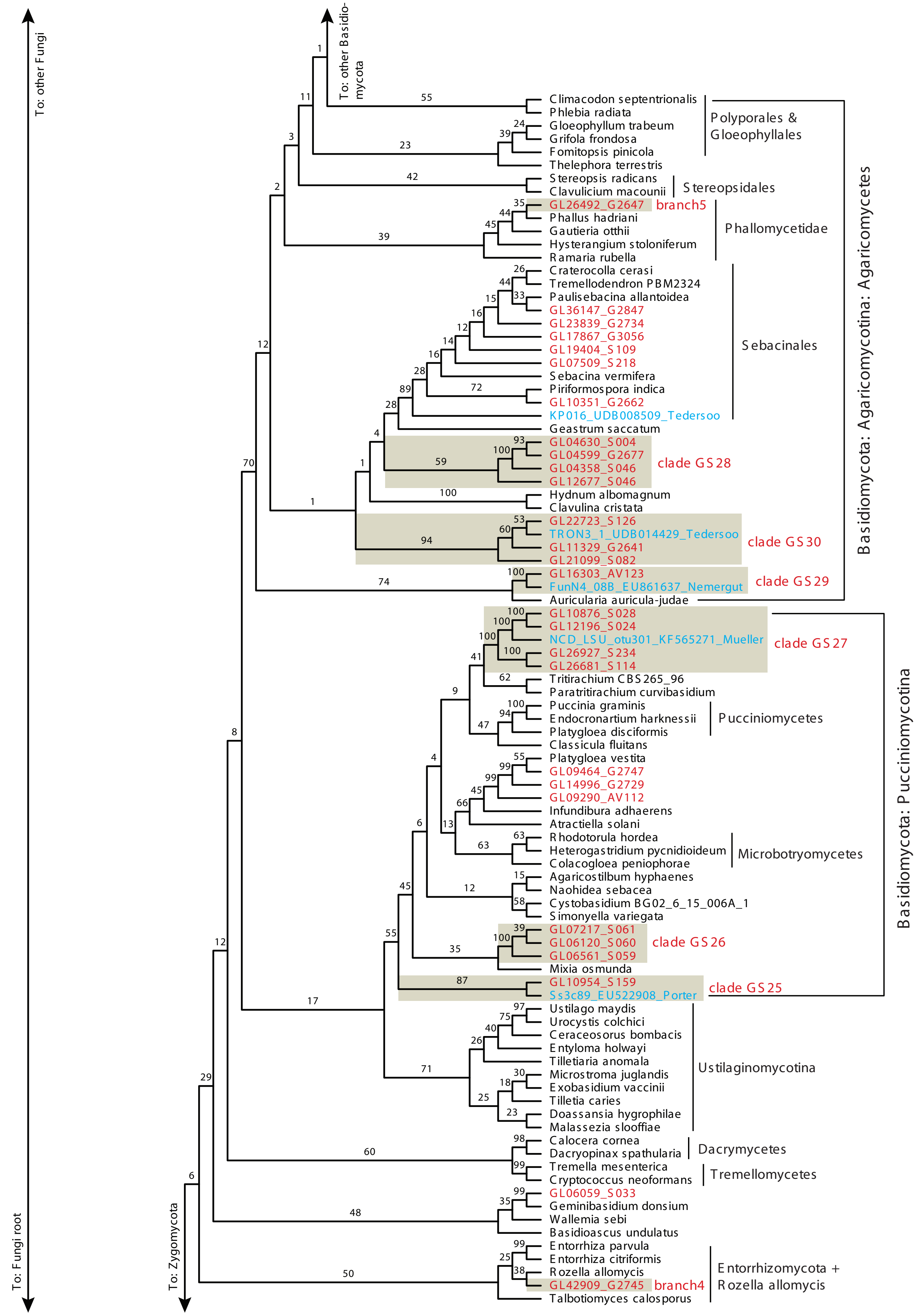
To: other Fungi
To: other Basidio-mycota
To: Fungi root
To: Zygomycota
Climacodon septentrionalis
Phlebia radiata
Gloeophyllum trabeum
Grifola frondosa
Fomitopsis pinicola
Thelephora terrestris
Polyporales & Gloeophyllales
Stereopsis radicans
Clavulicium macounii
Stereopsidales
GL26492_G2647
branch5
Phallus hadriani
Gautieria otthii
Hysterangium stoloniferum
Ramaria rubella
Phallomycetidae
Craterocolla cerasi
Tremellodendron PBM2324
Paulisebacina allantoidea
GL36147_G2847
GL23839_G2734
GL17867_G3056
GL19404_S109
GL07509_S218
Sebacina vermifera
Piriformospora indica
GL10351_G2662
KP016_UDB008509_Tedersoo
Sebacinales
Geastrum saccatum
GL04630_S004
GL04599_G2677
GL04358_S046
GL12677_S046
clade GS28
Hydnum albomagnum
Clavulina cristata
GL22723_S126
TRON3_1_UDB014429_Tedersoo
GL11329_G2641
GL21099_S082
clade GS30
GL16303_AV123
FunN4_08B_EU861637_Nemergut
clade GS29
Auricularia auricula-judae
Basidiomycota: Agaricomycotina: Agaricomycetes
GL10876_S028
GL12196_S024
NCD_LSU_otu301_KF565271_Mueller
GL26927_S234
GL26681_S114
clade GS27
Tritirachium CBS265_96
Paratritirachium curvibasidium
Puccinia graminis
Endocronartium harknessii
Platygloea disciformis
Pucciniomycetes
Classicula fluitans
Platygloea vestita
GL09464_G2747
GL14996_G2729
GL09290_AV112
Infundibura adhaerens
Atractiella solani
Rhodotorula hordea
Heterogastridium pycnidioideum
Colacogloea peniophorae
Microbotryomycetes
Agaricostilbum hyphaenes
Naohidea sebacea
Cystobasidium BG02_6_15_006A_1
Simonyella variegata
GL07217_S061
GL06120_S060
GL06561_S059
clade GS26
Mixia osmunda
GL10954_S159
Ss3c89_EU522908_Porter
clade GS25
Basidiomycota: Pucciniomycotina
Ustilago maydis
Urocystis colchici
Ceraceosorus bombacis
Entyloma holwayi
Tilletiaria anomala
Microstroma juglandis
Exobasidium vaccinii
Tilletia caries
Doassansia hygrophilae
Malassezia slooffiae
Ustilaginomycotina
Calocera cornea
Dacryopinax spathularia
Dacrymycetes
Tremella mesenterica
Cryptococcus neoformans
Tremellomycetes
GL06059_S033
Geminibasidium donsium
Wallemia sebi
Basidioascus undulatus
Entorrhiza parvula
Entorrhiza citriformis
Rozella allomycis
GL42909_G2745
branch4
Talbotiomyces calosporus
Entorrhizomycota + Rozella allomycis

Figure S3.4

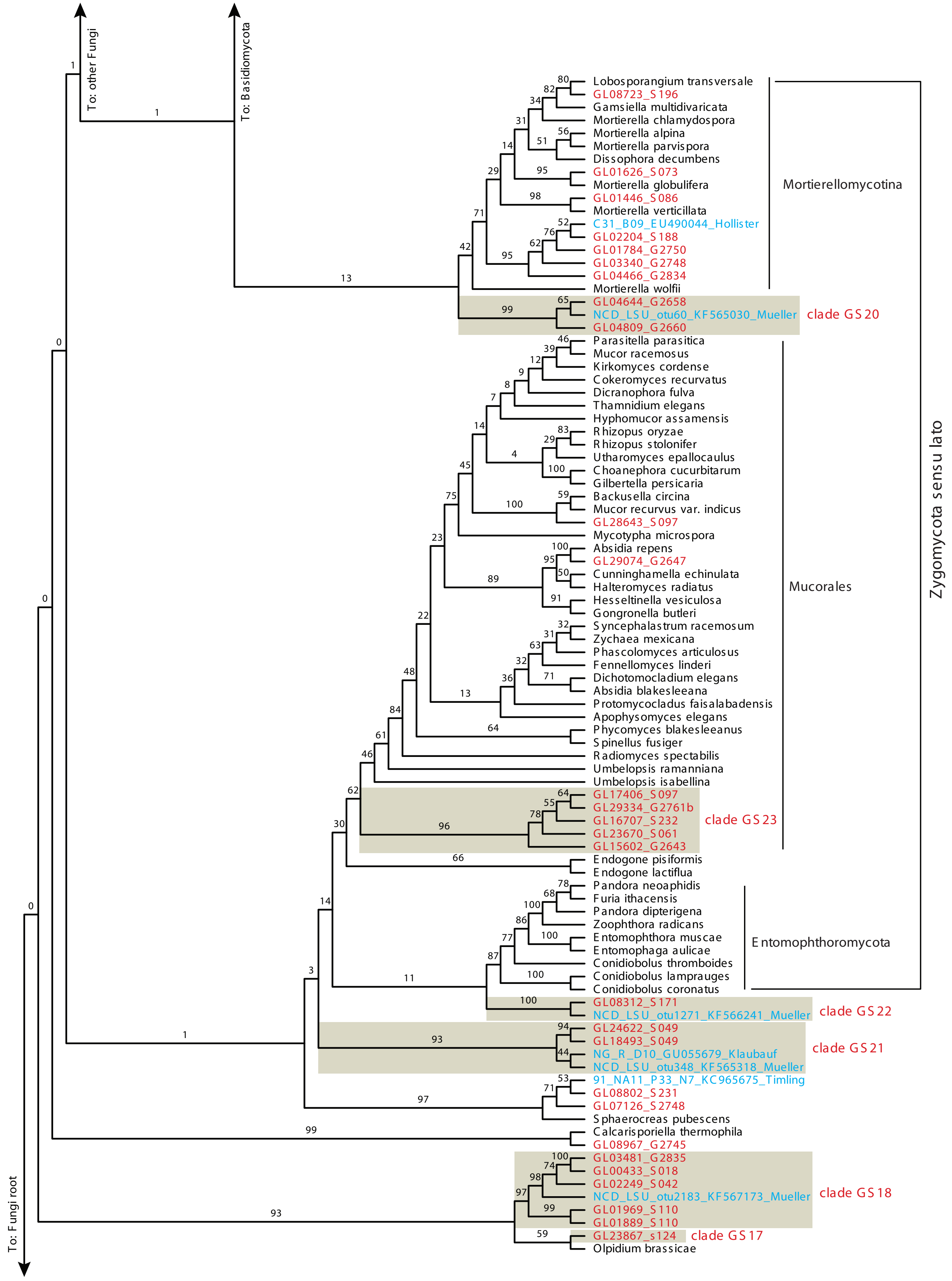
To: other Fungi
To: Basidiomycota
To: Fungi root
Lobosporangium transversale
GL08723_S196
Gamsiella multidivaricata
Mortierella chlamydospora
Mortierella alpina
Mortierella parvispora
Dissophora decumbens
GL01626_S073
Mortierella globulifera
GL01446_S086
Mortierella verticillata
C31_B09_EU490044_Hollister
GL02204_S188
GL01784_G2750
GL03340_G2748
GL04466_G2834
Mortierella wolfii
GL04644_G2658
NCD_LSU_otu60_KF565030_Mueller
GL04809_G2660
clade GS20
Mortierellomycotina
Parasitella parasitica
Mucor racemosus
Kirkomyces cordense
Cokeromyces recurvatus
Dicranophora fulva
Thamnidium elegans
Hyphomucor assamensis
Rhizopus oryzae
Rhizopus stolonifer
Utharomyces epallocaulus
Choanephora cucurbitarum
Gilbertella persicaria
Backusella circina
Mucor recurvus var. indicus
GL28643_S097
Mycotypha microspora
Absidia repens
GL29074_G2647
Cunninghamella echinulata
Halteromyces radiatus
Hesseltinella vesiculosa
Gongronella butleri
Syncephalastrum racemosum
Zychaea mexicana
Phascolomyces articulosus
Fennellomyces linderi
Dichotomocladium elegans
Absidia blakesleeana
Protomycocladus faisalabadensis
Apophysomyces elegans
Phycomyces blakesleeanus
Spinellus fusiger
Radiomyces spectabilis
Umbelopsis ramanniana
Umbelopsis isabellina
GL17406_S097
GL29334_G2761b
GL16707_S232
GL23670_S061
GL15602_G2643
clade GS23
Mucorales
Endogone pisiformis
Endogone lactiflua
Pandora neoaphidis
Furia ithacensis
Pandora dipterigena
Zoophthora radicans
Entomophthora muscae
Entomophaga aulicae
Conidiobolus thromboides
Conidiobolus lamprauges
Conidiobolus coronatus
Entomophthoromycota
Zygomycota sensu lato
GL08312_S171
NCD_LSU_otu1271_KF566241_Mueller
clade GS22
GL24622_S049
GL18493_S049
NG_R_D10_GU055679_Klaubauf
NCD_LSU_otu348_KF565318_Mueller
clade GS21
91_NA11_P33_N7_KC965675_Timling
GL08802_S231
GL07126_S2748
Sphaerocreas pubescens
Calcarisporiella thermophila
GL08967_G2745
GL03481_G2835
GL00433_S018
GL02249_S042
NCD_LSU_otu2183_KF567173_Mueller
GL01969_S110
GL01889_S110
clade GS18
GL23867_s124
clade GS17
Olpidium brassicae

Figure S3.5

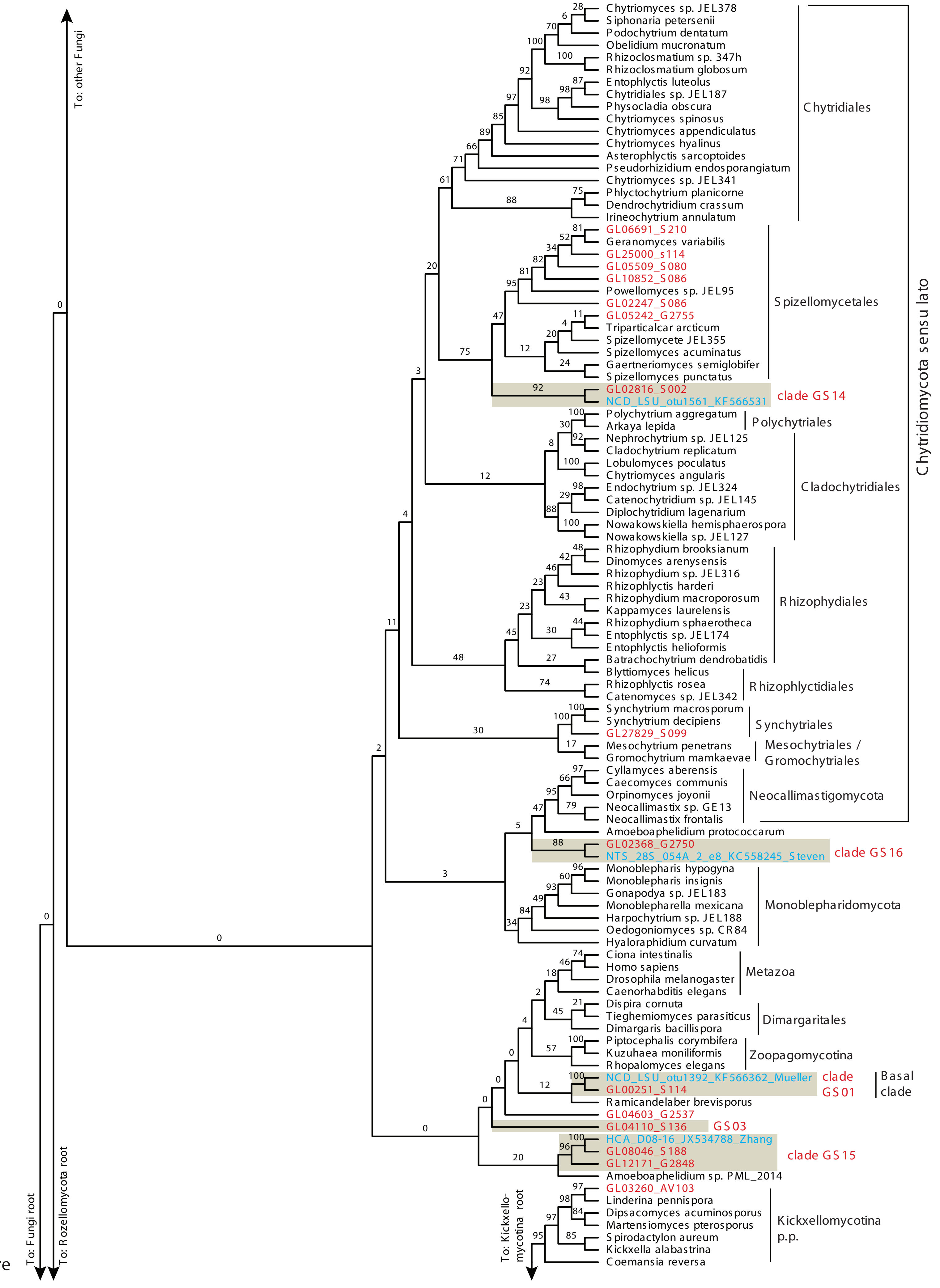

To: other Fungi
Chytriomyces sp. JEL378
Siphonaria petersenii
Podochytrium dentatum
Obelidium mucronatum
Rhizoclosmatium sp. 347h
Rhizoclosmatium globosum
Entophlyctis luteolus
Chytridiales sp. JEL187
Physocladia obscura
Chytriomyces spinosus
Chytriomyces appendiculatus
Chytriomyces hyalinus
Asterophlyctis sarcoptoides
Pseudorhizidium endosporangiatum
Chytriomyces sp. JEL341
Phlyctochytrium planicorne
Dendrochytridium crassum
Irineochytrium annulatum
Chytridiales
GL06691_S210
Geranomyces variabilis
GL25000_s114
GL05509_S080
GL10852_S086
Powellomyces sp. JEL95
GL02247_S086
GL05242_G2755
Triparticalcar arcticum
Spizellomycete JEL355
Spizellomyces acuminatus
Gaertneriomyces semiglobifer
Spizellomyces punctatus
Spizellomycetales
GL02816_S002
NCD_LSU_otu1561_KF566531
clade GS14
Polychytrium aggregatum
Arkaya lepida
Polychytriales
Nephrochytrium sp. JEL125
Cladochytrium replicatum
Lobulomyces poculatus
Chytriomyces angularis
Endochytrium sp. JEL324
Catenochytridium sp. JEL145
Diplochytridium lagenarium
Nowakowskiella hemisphaerospora
Nowakowskiella sp. JEL127
Cladochytridiales
Rhizophydium brooksianum
Dinomyces arenysensis
Rhizophydium sp. JEL316
Rhizophlyctis harderi
Rhizophydium macroporosum
Kappamyces laurelensis
Rhizophydium sphaerotheca
Entophlyctis sp. JEL174
Entophlyctis helioformis
Batrachochytrium dendrobatidis
Rhizophydiales
Blyttiomyces helicus
Rhizophlyctis rosea
Catenomyces sp. JEL342
Rhizophlyctidiales
Synchytrium macrosporum
Synchytrium decipiens
GL27829_S099
Synchytriales
Mesochytrium penetrans
Gromochytrium mamkaevae
Mesochytriales / Gromochytriales
Cyllamyces aberensis
Caecomyces communis
Orpinomyces joyonii
Neocallimastix sp. GE13
Neocallimastix frontalis
Neocallimastigomycota
Chytridiomycota sensu lato
Amoeboaphelidium protococcarum
GL02368_G2750
NTS_28S_054A_2_e8_KC558245_Steven
clade GS16
Monoblepharis hypogyna
Monoblepharis insignis
Gonapodya sp. JEL183
Monoblepharella mexicana
Harpochytrium sp. JEL188
Oedogoniomyces sp. CR84
Hyaloraphidium curvatum
Monoblepharidomycota
Ciona intestinalis
Homo sapiens
Drosophila melanogaster
Caenorhabditis elegans
Metazoa
Dispira cornuta
Tieghemiomyces parasiticus
Dimargaris bacillispora
Dimargaritales
Piptocephalis corymbifera
Kuzuhaea moniliformis
Rhopalomyces elegans
Zoopagomycotina
NCD_LSU_otu1392_KF566362_Mueller
GL00251_S114
clade GS01
Basal clade
Ramicandelaber brevisporus
GL04603_G2537
GL04110_S136
GS03
HCA_D08-16_JX534788_Zhang
GL08046_S188
GL12171_G2848
clade GS15
Amoeboaphelidium sp. PML_2014
GL03260_AV103
Linderina pennispora
Dipsacomyces acuminosporus
Martensiomyces pterosporus
Spirodactylon aureum
Kickxella alabastrina
Coemansia reversa
Kickxellomycotina p.p.
To: Kickxello-mycotina root
To: Fungi root
To: Rozellomycota root

Figure S3.6

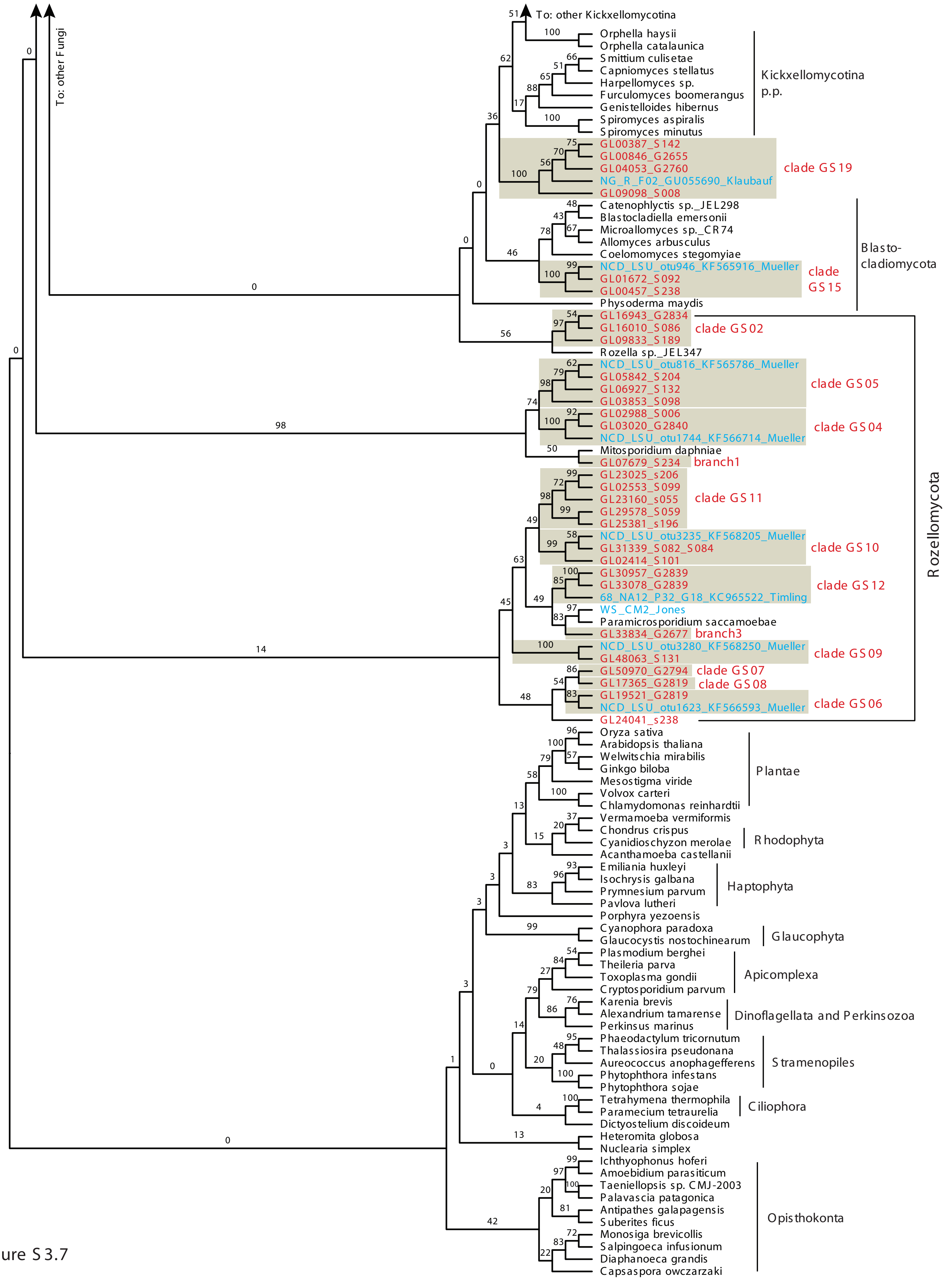

To: other Fungi
To: other Kickxellomycotina
Orphella haysii
Orphella catalaunica
Smittium culisetae
Capniomyces stellatus
Harpellomyces sp.
Furculomyces boomerangus
Genistelloides hibernus
Spiromyces aspiralis
Spiromyces minutus
Kickxellomycotina p.p.
GL00387_S142
GL00846_G2655
GL04053_G2760
NG_R_F02_GU055690_Klaubauf
GL09098_S008
clade GS19
Catenophlyctis sp._JEL298
Blastocladiella emersonii
Microallomyces sp._CR74
Allomyces arbusculus
Coelomomyces stegomyiae
NCD_LSU_otu946_KF565916_Mueller
GL01672_S092
GL00457_S238
clade GS15
Blasto-cladiomycota
Physoderma maydis
GL16943_G2834
GL16010_S086
GL09833_S189
clade GS02
Rozella sp._JEL347
NCD_LSU_otu816_KF565786_Mueller
GL05842_S204
GL06927_S132
GL03853_S098
clade GS05
GL02988_S006
GL03020_G2840
NCD_LSU_otu1744_KF566714_Mueller
clade GS04
Mitosporidium daphniae
GL07679_S234
branch1
GL23025_s206
GL02553_S099
GL23160_s055
GL29578_S059
GL25381_s196
clade GS11
NCD_LSU_otu3235_KF568205_Mueller
GL31339_S082_S084
GL02414_S101
clade GS10
GL30957_G2839
GL33078_G2839
68_NA12_P32_G18_KC965522_Timling
clade GS12
WS_CM2_Jones
Paramicrosporidium saccamoebae
GL33834_G2677
branch3
NCD_LSU_otu3280_KF568250_Mueller
GL48063_S131
clade GS09
GL50970_G2794
clade GS07
GL17365_G2819
clade GS08
GL19521_G2819
NCD_LSU_otu1623_KF566593_Mueller
clade GS06
GL24041_s238
Rozellomycota
Oryza sativa
Arabidopsis thaliana
Welwitschia mirabilis
Ginkgo biloba
Mesostigma viride
Volvox carteri
Chlamydomonas reinhardtii
Plantae
Vermamoeba vermiformis
Chondrus crispus
Cyanidioschyzon merolae
Rhodophyta
Acanthamoeba castellanii
Emiliania huxleyi
Isochrysis galbana
Prymnesium parvum
Pavlova lutheri
Haptophyta
Porphyra yezoensis
Cyanophora paradoxa
Glaucocystis nostochinearum
Glaucophyta
Plasmodium berghei
Theileria parva
Toxoplasma gondii
Cryptosporidium parvum
Apicomplexa
Karenia brevis
Alexandrium tamarense
Perkinsus marinus
Dinoflagellata and Perkinsozoa
Phaeodactylum tricornutum
Thalassiosira pseudonana
Aureococcus anophagefferens
Phytophthora infestans
Phytophthora sojae
Stramenopiles
Tetrahymena thermophila
Paramecium tetraurelia
Ciliophora
Dictyostelium discoideum
Heteromita globosa
Nuclearia simplex
Ichthyophonus hoferi
Amoebidium parasiticum
Taeniellopsis sp. CMJ-2003
Palavascia patagonica
Antipathes galapagensis
Suberites ficus
Monosiga brevicollis
Salpingoeca infusionum
Diaphanoeca grandis
Capsaspora owczarzaki
Opisthokonta

Figure S3.7

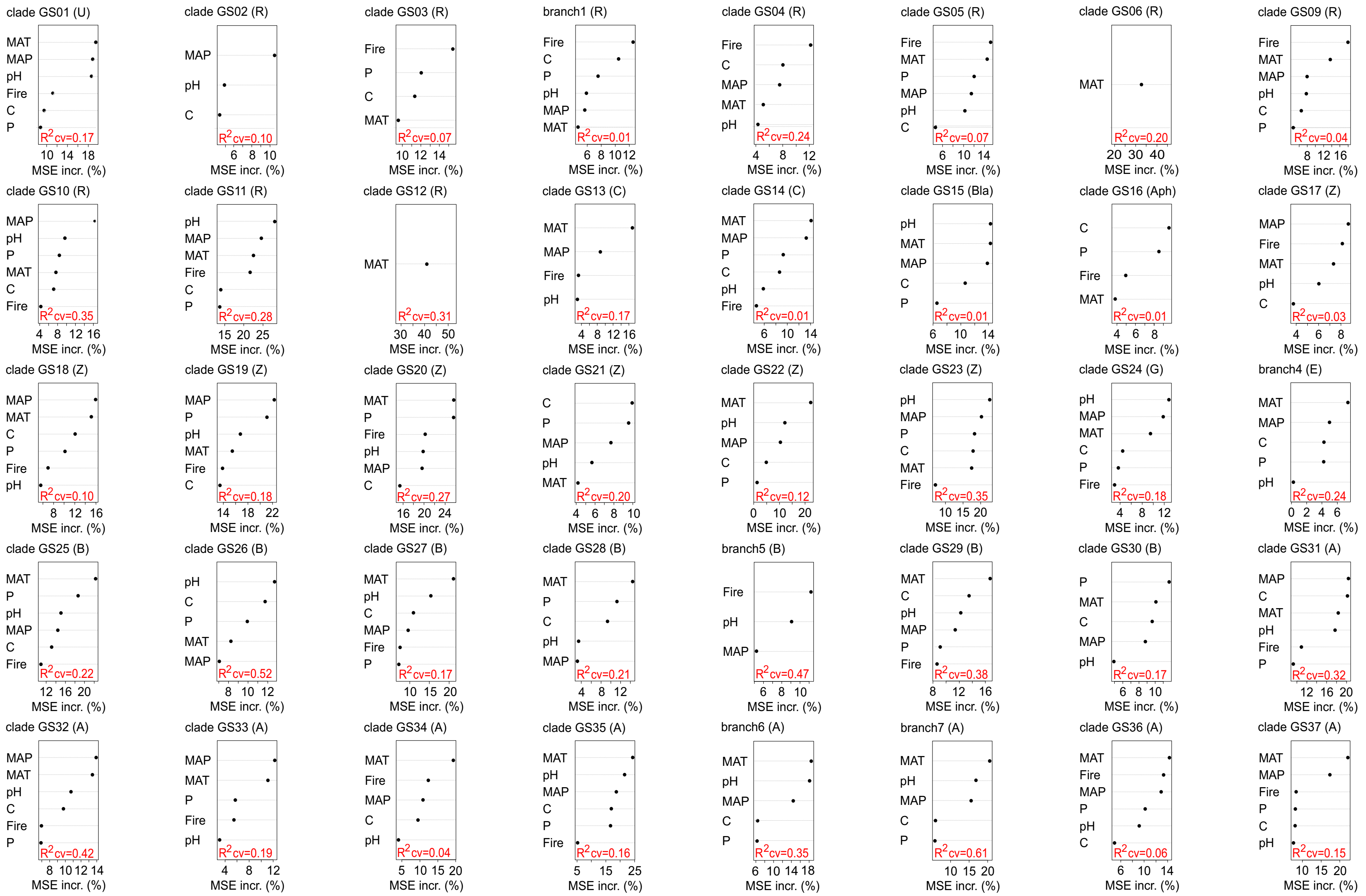

Figure S4. Best models of Random Forest machine learning-based niche analysis of fungal clades and prominent branches. The values on x-axis indicate increase in the mean square error (MSE, %). R2cv represents the cross-validation accuracy index. Clade GS08 is not shown, because none of the variables were included in the model. Clade GS07 and branches 2 and 3 were not tested, because these were represented by a single OTU. Names of phyla are abbreviated in parentheses: A, Ascomycota; Aph, Aphelida; B, Basidiomycota; Bla, Blastocladiomycota; C, Chytridiomycota; E, Entorrhizomycota; G, Glomeromycota; R, Rozellomycota; Z, Zygomycota s.lat.; U, unassigned.

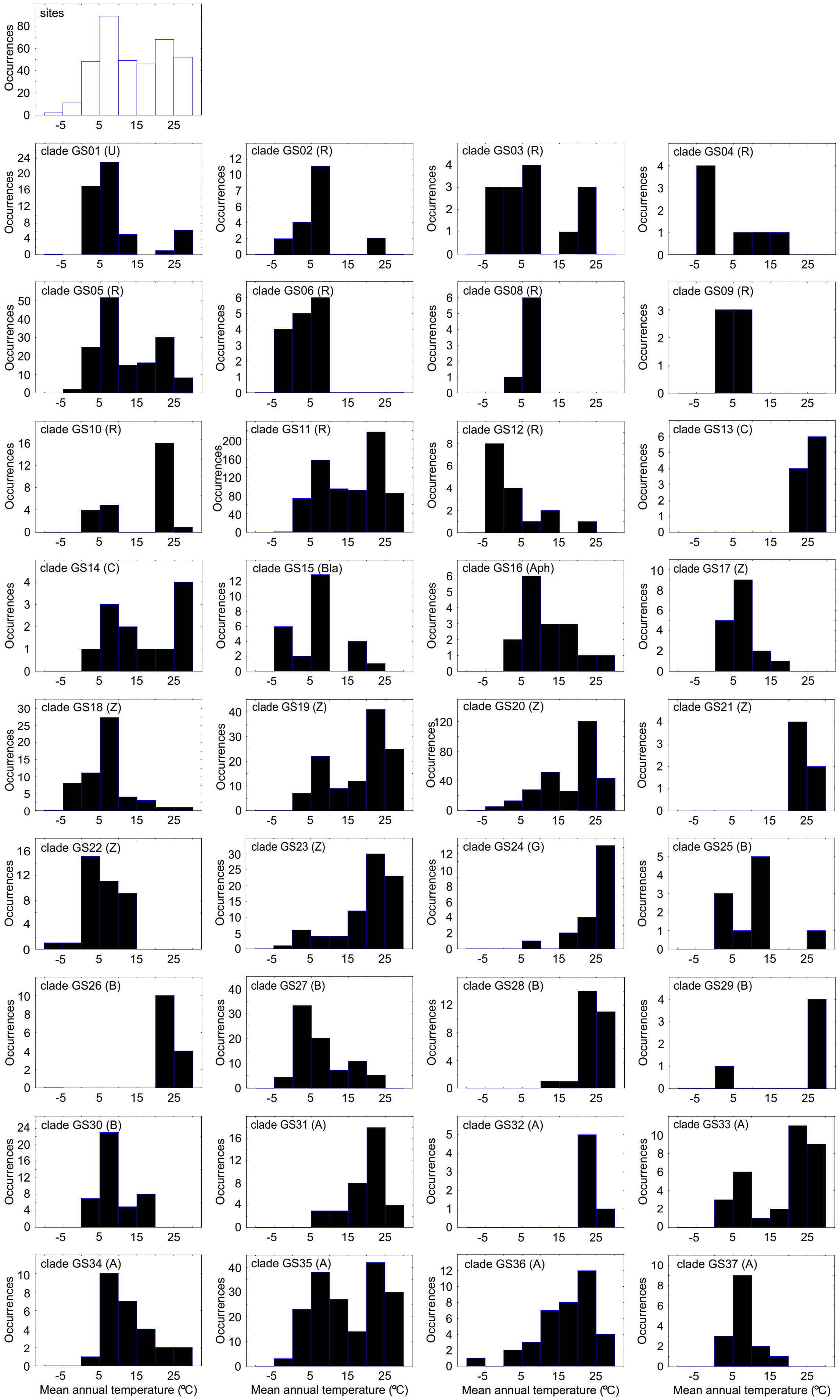

Figure S5. Histograms indicating the distribution of fungal clades (summed occurrences of OTUs) in sites with specified mean annual temperature. The upleft graph indicates the climatic coverage of study sites (null distribution). Names of phyla are abbreviated in parentheses: A, Ascomycota; Aph, Aphelida; B, Basidiomycota; Bla, Blastocladiomycota; C, Chytridiomycota; E, Entorrhizomycota; G, Glomeromycota; R, Rozellomycota; Z, Zygomycota s.lat.; U, unassigned.

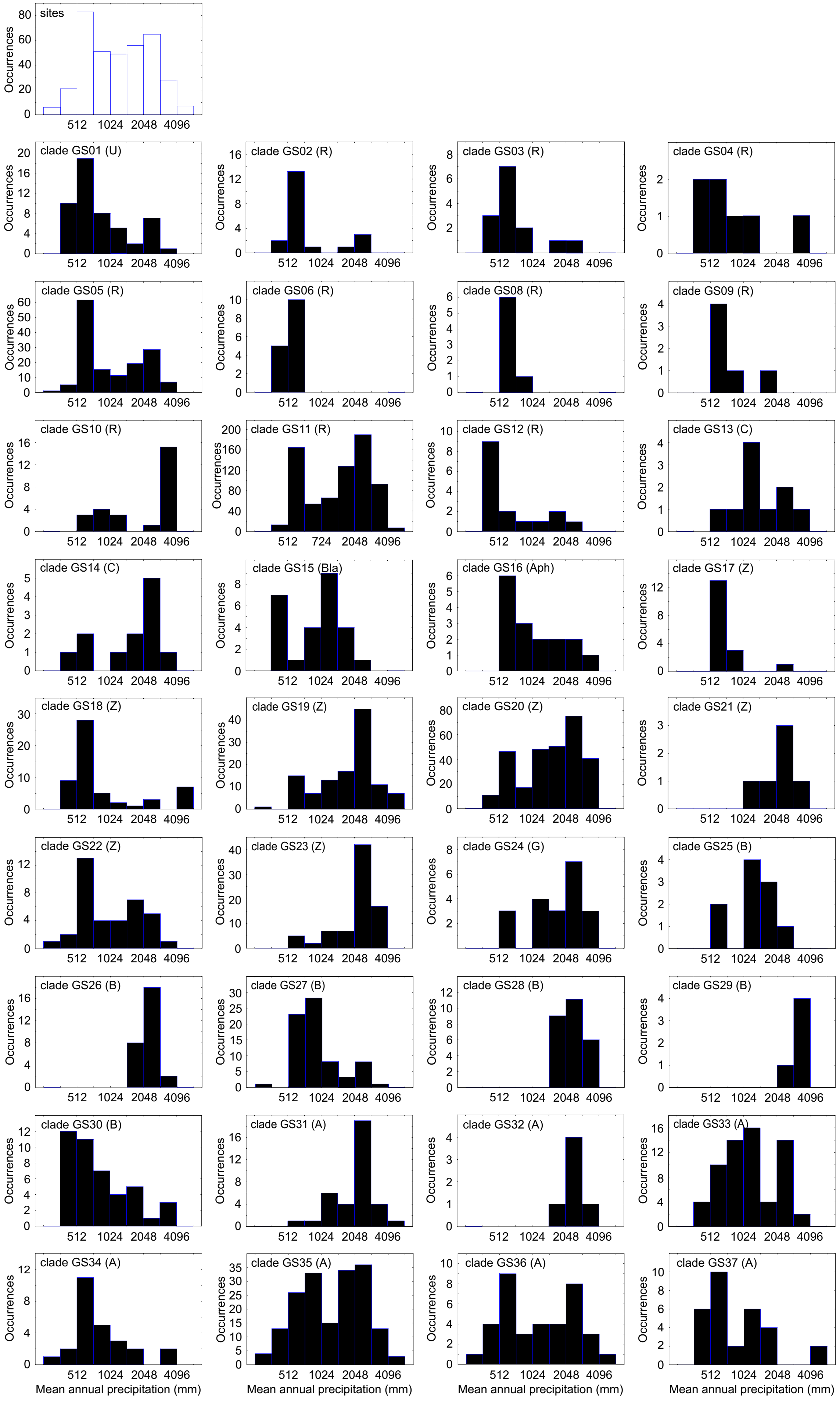

Figure S6. Histograms indicating the distribution of fungal clades (summed occurrences of OTUs) in sites with specified mean annual precipitation. The upleft graph indicates the climatic coverage of study sites (null distribution). Note the 2-base logaritmic scale. Names of phyla are abbreviated in parentheses: A, Ascomycota; Aph, Aphelida; B, Basidiomycota; Bla, Blastocladiomycota; C, Chytridiomycota; E, Entorrhizomycota; G, Glomeromycota; R, Rozellomycota; Z, Zygomycota s.lat.; U, unassigned.

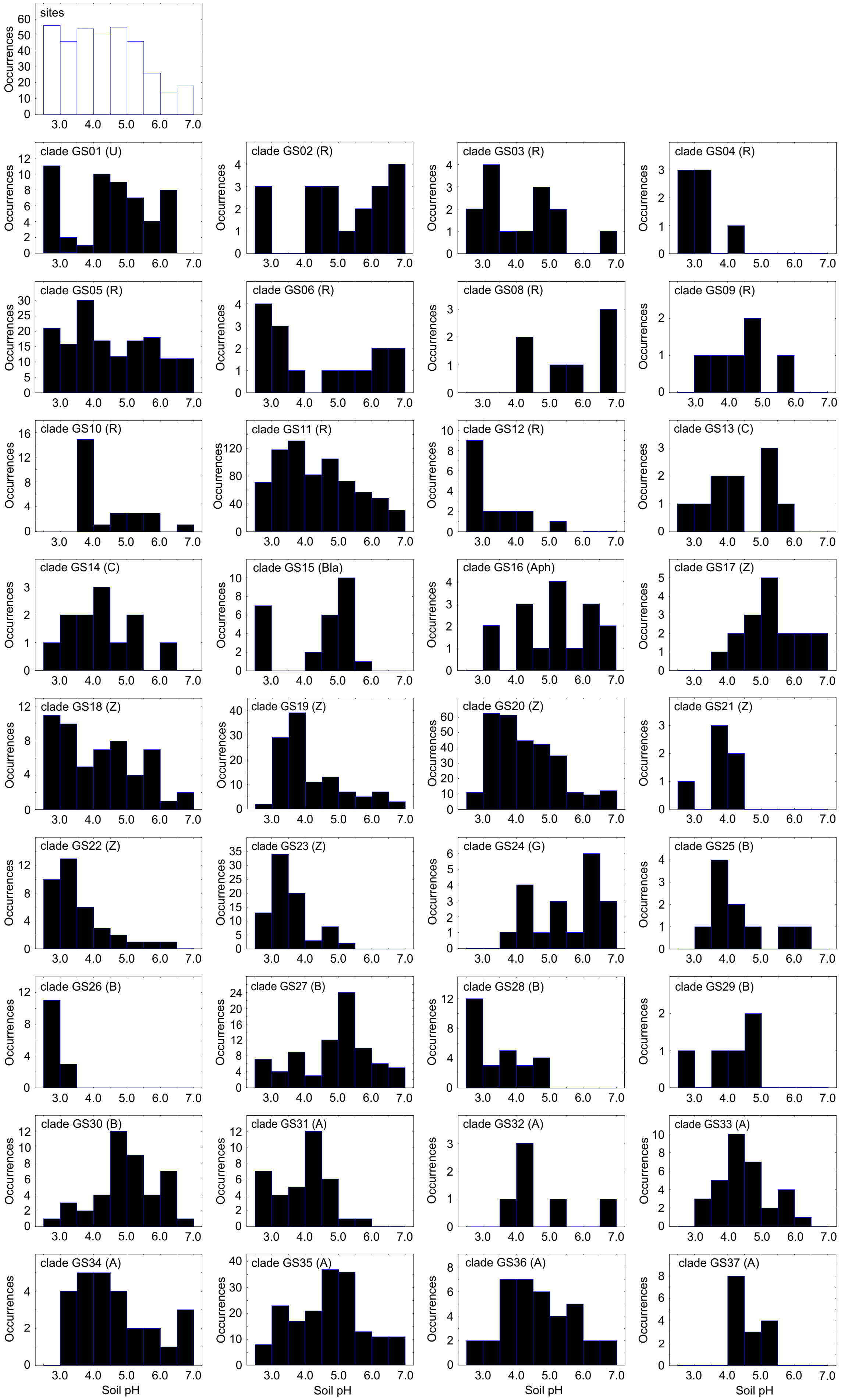

Figure S7. Histograms indicating the distribution of fungal clades (summed occurrences of OTUs) in sites with specified soil pH. The upleft graph indicates the pH coverage of study sites (null distribution). Names of phyla are abbreviated in parentheses: A, Ascomycota; Aph, Aphelida; B, Basidiomycota; Bla, Blastocladiomycota; C, Chytridiomycota; E, Entorrhizomycota; G, Glomeromycota; R, Rozellomycota; Z, Zygomycota s.lat.; U, unassigned.

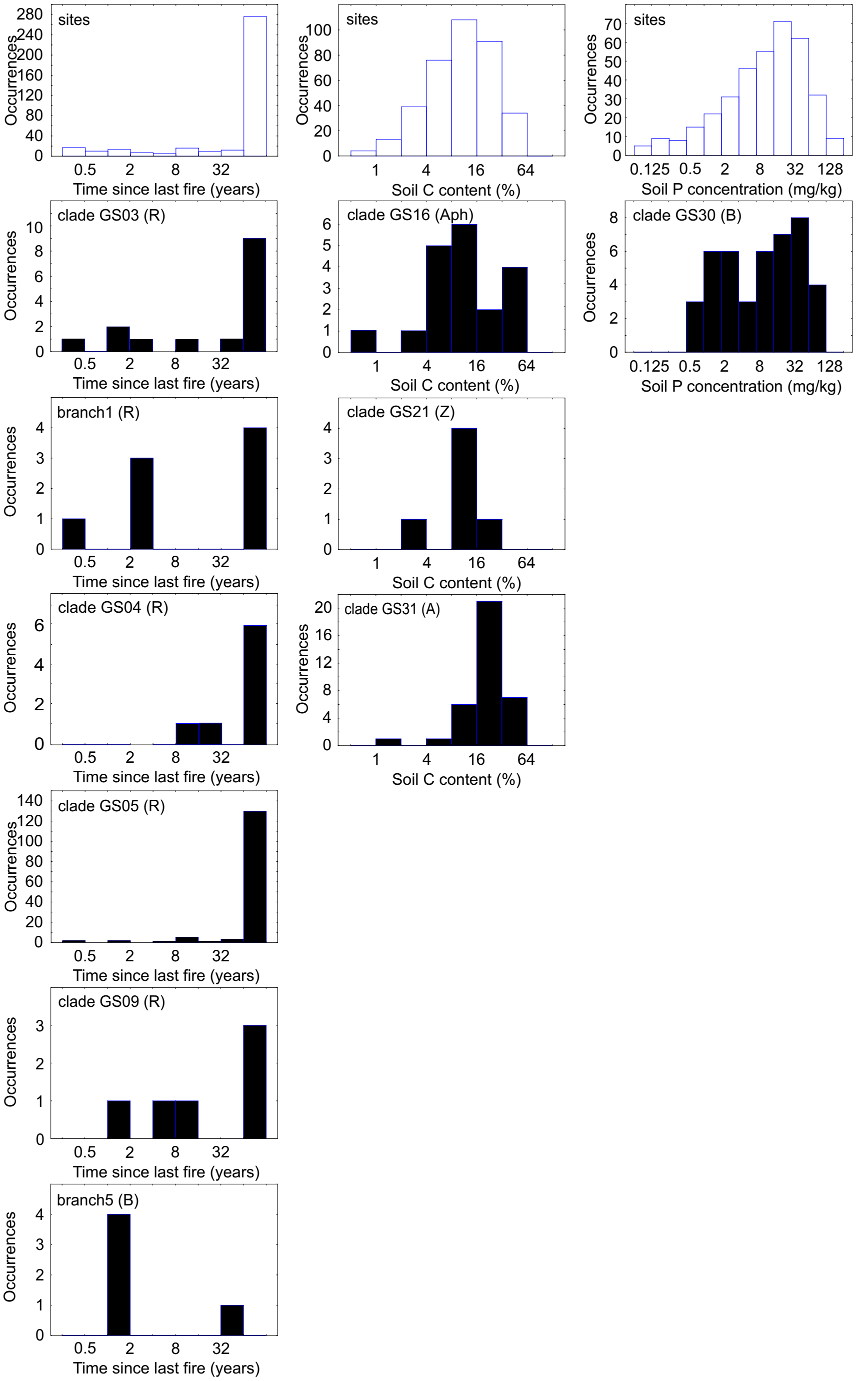

Figure S8. Histograms indicating the distribution of fungal clades (summed occurrences of OTUs) in sites with specified time since last fire (left panels), soil carbon content (central panels), and soil phosphorus concentration (right panels). The upmost diagrams indicates the distribution of these variables across all study sites (null distribution). Note the 2-base logaritmic scale. Names of phyla are abbreviated in parentheses: A, Ascomycota; Aph, Aphelida; B, Basidiomycota; Bla, Blastocladiomycota; C, Chytridiomycota; E, Entorrhizomycota; G, Glomeromycota; R, Rozellomycota; Z, Zygomycota s.lat.; U, unassigned.

Text S1. Profiles of undescribed clades and prominent branches of fungi.

Clade GS01

Previous names for the clade: -
Corresponding PlutoF compound cluster: -

Representative sequence: UDB014611 (habitat: site S114; Czech Republic; 49.0174 N/ 13.4751 W; montane temperate coniferous forest dominated by *Picea abies*)
Contains other sequences: UDB014707, UDB014621, KF566362

Phylogenetic position: Along with an orphan sequence HQ191300 forms a sister group to the rest of the Fungi, albeit with no support. Group-specific 18S rRNA gene primers near the V9 subregion: fwd ACCGCCCGTCGCTRTTT; rev CTAAGCCATYCAATCGGAAA (specific positions underlined); several mismatches to ITS1F, ITS1, ITS2, and ITS3 primers

Ecological habitat: cool temperate climate over a wide pH range
Distribution: detected from most regions and biomes; strikingly more common in Europe and Southern South America

Other habitats: temperate forest soil in NC, USA (KF566362)

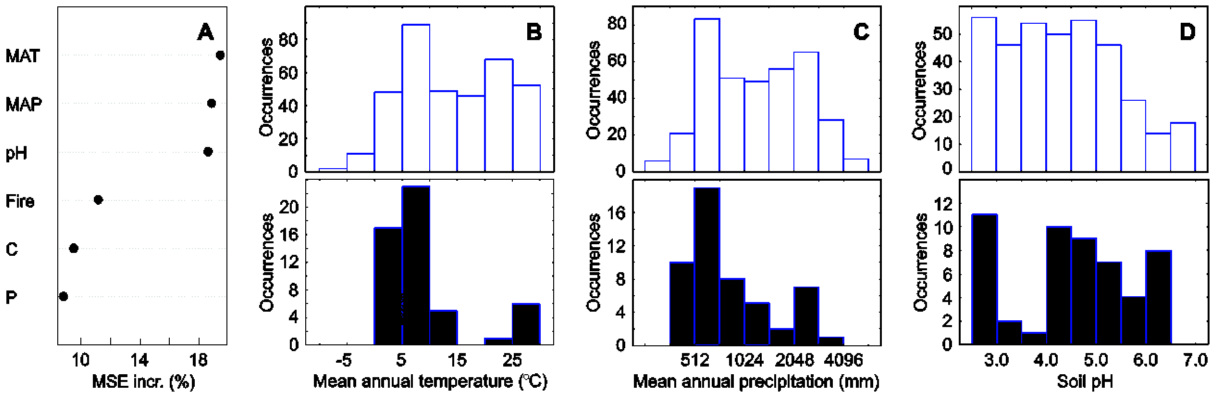

Figure. Niche analysis of the clade. A. Strongest predictors for the occurrence of clade members based on random forest analysis. B-D. Distribution of clade members (lower panels) in sites with (B) different mean annual temperature, (C) mean annual precipitation, and (D) average soil pH relative to all studied soils (upper panels).

Text S1.1

Clade GS02

Previous names for the clade: -
Corresponding PlutoF compound cluster: UCL7_001709

Representative sequence: UDB014756 (habitat: site S189; BJ, China; 42.4292 N/ 128.1167 E); montane temperate coniferous forest dominated by *Pinus sylvestriformis*)
Contains other sequences: UDB014795, UDB014794, GU067982, FJ976648

Phylogenetic position: a sister clade to the genus *Rozella* but with poor support. The sequences produced in this study form a well-supported monophyletic group.

Ecological habitat: prefers low to moderate rainfall and neutral and weakly acidic soils in temperate deciduous forests
Distribution: no significant trends but mostly found in Europe

Other habitats: freshwater lake (GU067982) and peat bog (FJ976648) in Europe

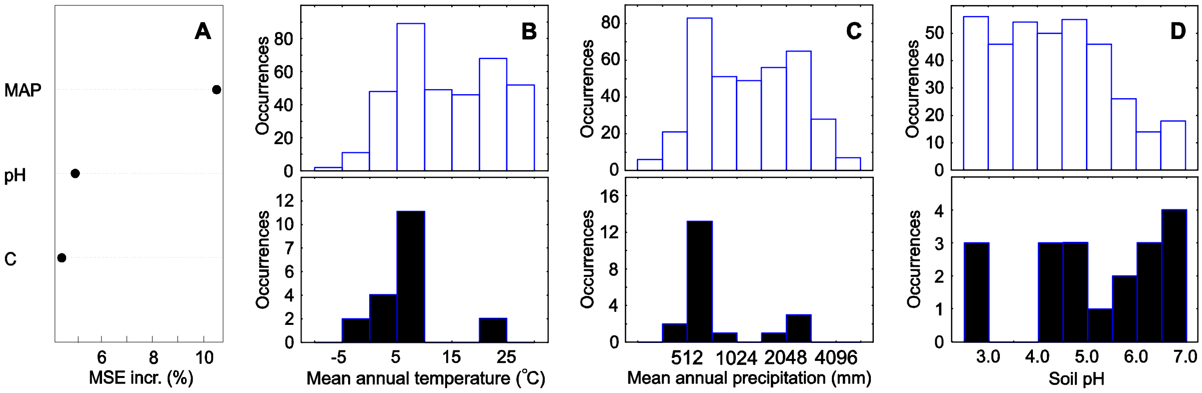

Figure. Niche analysis of the clade. A. Strongest predictors for the occurrence of clade members based on random forest analysis. B-D. Distribution of clade members (lower panels) in sites with (B) different mean annual temperature, (C) mean annual precipitation, and (D) average soil pH relative to all studied soils (upper panels).

Clade GS03

Previous names for the clade: -
Corresponding PlutoF compound cluster: -

Representative sequence: UDB014679 (habitat: site S136; Udmurtia, Russian Fed.; 57.1041 N/ 53.0757 E; boreal forest dominated by *Pinus sylvestris*)
Contains other sequences: AB695514

Phylogenetic position: one of the branches of Rozellomycota

Ecological habitat: relatively common in sites with recent fire. The clade tends to be more frequent in relatively cold habitats such as tundra and boreal forest biomes. Other studies suggest widespread distribution in saltwater, freshwater, and anoxic habitats.
both in extremely cold and hot ecosystems.

Distribution: present in most regions

Other habitats: Antarctic freshwater lake (AB695514)

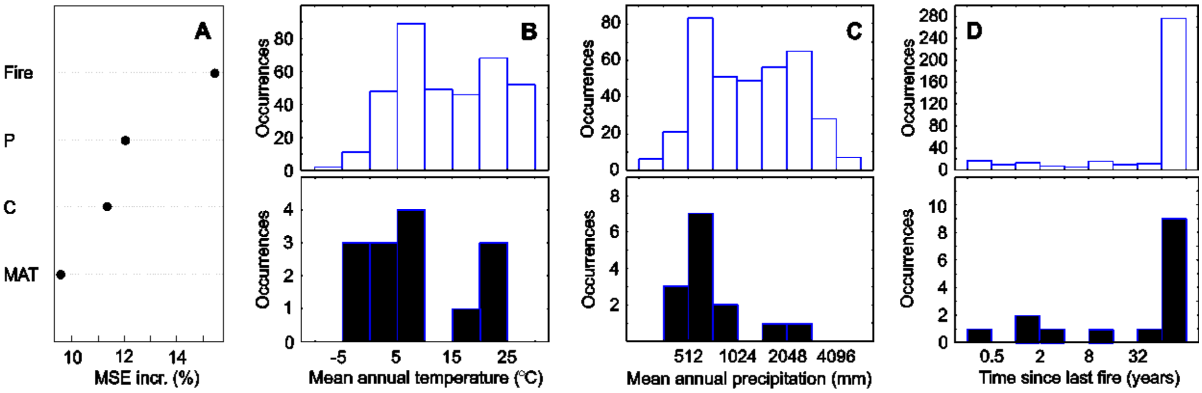

Figure. Niche analysis of the clade. A. Strongest predictors for the occurrence of clade members based on random forest analysis. B-D. Distribution of clade members (lower panels) in sites with (B) different mean annual temperature, (C) mean annual precipitation, and (D) average time since last fire relative to all studied soils (upper panels).

Text S1.3

Branch1

Previous names for the clade: -
Corresponding PlutoF compound cluster: -

Representative sequence: UDB014728 (habitat: site S234; Finland; 69.1691 N/ 26.9992 E; southern tundra dominated by dwarf *Betula pubescens*)
Contains other sequences: -

Phylogenetic position: A single deep branch within Rozellomycota

Ecological habitat: tolerates recent fire and cold; mostly found in tropical habitats.
Distribution: most frequent in Central America.

Other habitats: -

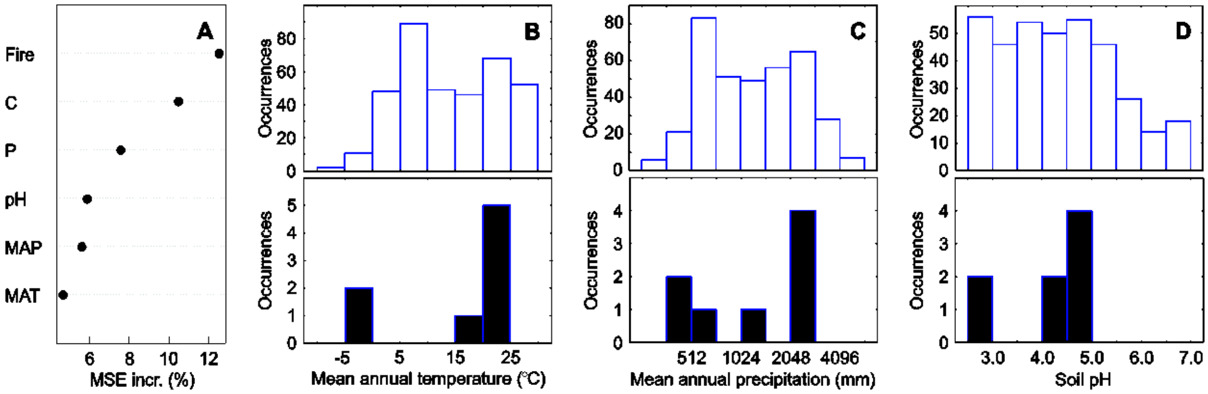

Figure. Niche analysis of the clade. A. Strongest predictors for the occurrence of clade members based on random forest analysis. B-D. Distribution of clade members (lower panels) in sites with (B) different mean annual temperature, (C) mean annual precipitation, and (D) average soil pH relative to all studied soils (upper panels).

Clade GS04

Previous names for the clade: -
Corresponding PlutoF compound cluster: -

Representative sequence: UDB014664 (habitat: site G2840; Finland; 68.0636 N/ 24.0339 E; mountain tundra dominated by *Betula nana*)
Contains other sequences: UDB014656, UDB014663, AB534337, KF566714

Phylogenetic position: one of the groups related to Mitosporidium within Rozellomycota

Ecological habitat: tends to be most abundant in the tundra biome in high-carbon soils
Distribution: present in cold and temperate habitats of the Northern and Southern Hemisphere

Other habitats: agricultural soil in Japan (AB534337), temperate forest soil in NC, USA (KF566714)

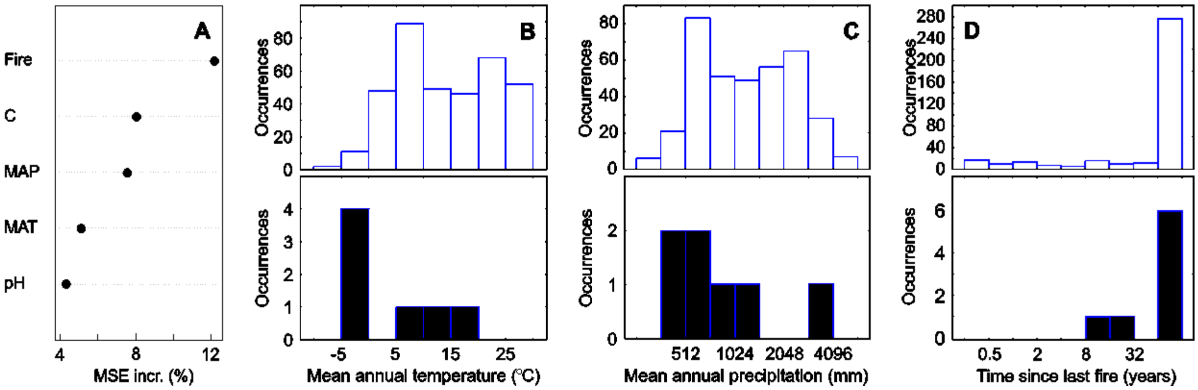

Figure. Niche analysis of the clade. A. Strongest predictors for the occurrence of clade members based on random forest analysis. B-D. Distribution of clade members (lower panels) in sites with (B) different mean annual temperature, (C) mean annual precipitation, and (D) average soil C content relative to all studied soils (upper panels).

Clade GS05

Previous names for the clade: LKM15 (van Hannen et al. 1999, Appl. Environ. Microbiol. 65: 2478; Panzer et al. 2015, PLoS ONE: e0134377)
Corresponding PlutoF compound cluster: -

Representative sequence: UDB014721 (habitat: site S132; VO, Russia; 51.9557 N/ 39.4676; temperate coniferous forest dominated by *Pinus sylvestris*)
Contains other sequences: UDB014673, UDB014705; AF372718, AF372717, AJ130850, EF023121, EF023117, EU910604, HM487053, HM487011, KF565786

Phylogenetic position: one of the groups related to Mitosporidium within Rozellomycota

Ecological habitat: frequent in all biomes, especially temperate deciduous forests; not observed in tundra; wide pH tolerance; relatively more common in unburnt sites.
Distribution: frequent in all ecoregions, especially in Europe and West Asia, but relatively less common in the Southern Hemisphere. Other studies have recovered it from lake and marine sediments and water.

Other habitats: amphipod feces (HM487053, HM487011), soil in deciduous forests of USA (HM487…, KF565786), temperate marine sediments (AF372…), temperate lake sediments (EU910604), and freshwater mesocosms (AJ130850)

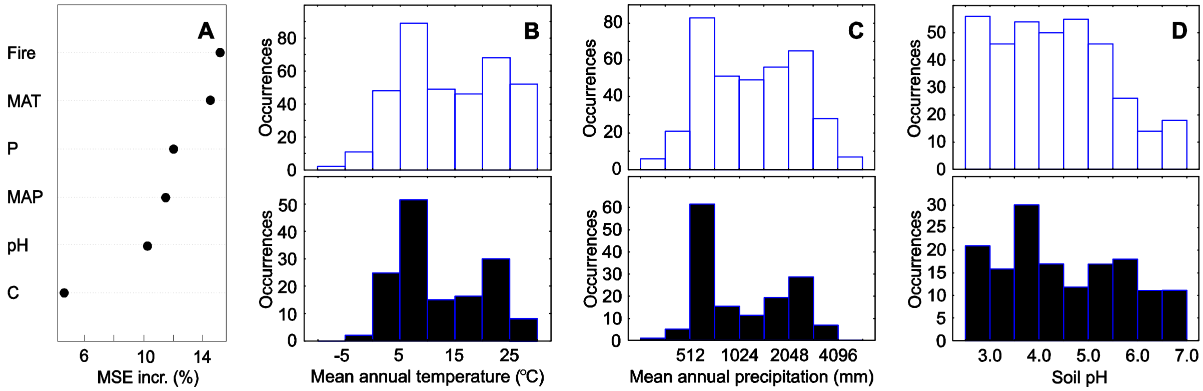

Figure. Niche analysis of the clade. A. Strongest predictors for the occurrence of clade members based on random forest analysis. B-D. Distribution of clade members (lower panels) in sites with (B) different mean annual temperature, (C) mean annual precipitation, and (D) average soil pH relative to all studied soils (upper panels).

Clade GS06

Previous names for the clade: -
Corresponding PlutoF compound cluster: -

Representative sequence: UDB014815 (habitat: site G2819; Estonia; 58.2341 N/ 22.4411 E; overgrown temperate woodland dominated by *Quercus robur*)
Contains other sequences: UDB014850, KF566593

Phylogenetic position: a clade within a large group of unidentified Rozellomycota

Ecological habitat: found mainly in boreal forest and tundra ecosystems across a wide range of soil pH.
Distribution: found only in Europe but also in North America based on another study.

Other habitats: temperate forest soil in NC, USA (KF566593)

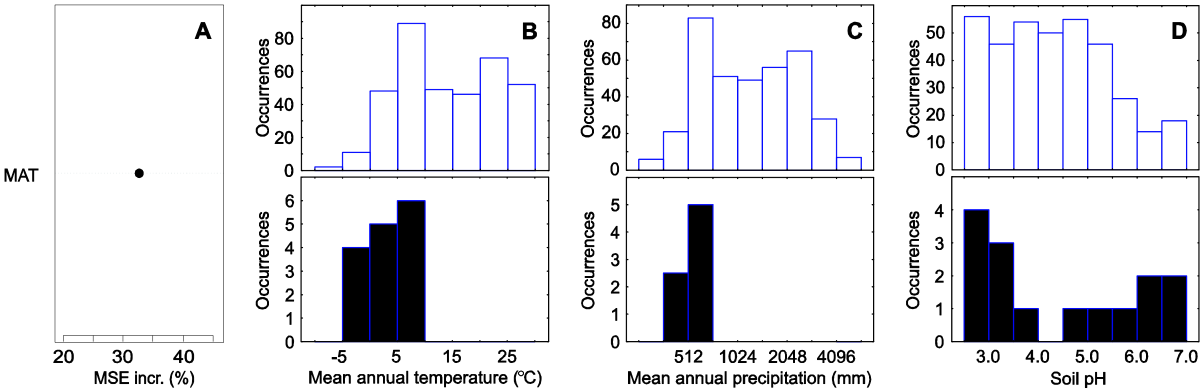

Figure. Niche analysis of the clade. A. Strongest predictors for the occurrence of clade members based on random forest analysis. B-D. Distribution of clade members (lower panels) in sites with (B) different mean annual temperature, (C) mean annual precipitation, and (D) average soil pH relative to all studied soils (upper panels).

Clade GS07

Previous names for the clade: -
Corresponding PlutoF compound cluster: -

Representative sequence: UDB014956 (habitat: site G2794; Estonia; 58.4865 N/ 22.8318 E; moderately grazed woodland dominated by *Corylus avellana*)
Contains other sequences: GQ995417

Phylogenetic position: a clade within a large group of unidentified Rozellomycota

Ecological habitat: probably very broad but not tested, because this clade was represented by a single OTU in this study
Distribution: Represented by a single widespread OTU with distribution in Europe, Southern South America, and Central Africa

Other habitats: alpine tundra soil from CO, USA, or Nepal (not specified by the sequence authors; GQ995417)

Clade GS08

Previous names for the clade: -
Corresponding PlutoF compound cluster: -

Representative sequence: UDB014958 (habitat: site G2819; Estonia; 58.2341 N/ 22.4411 E; overgrown temperate woodland dominated by *Quercus robur*)
Contains other sequences: FM178232

Phylogenetic position: a clade within a large group of unidentified Rozellomycota

Ecological habitat: no significant patterns; the clade is distributed in the cool temperate coniferous and deciduous forests.
Distribution: known only from Europe and East Asia.

Other habitats: aerobic sludge granules in Central Europe (FM178232)

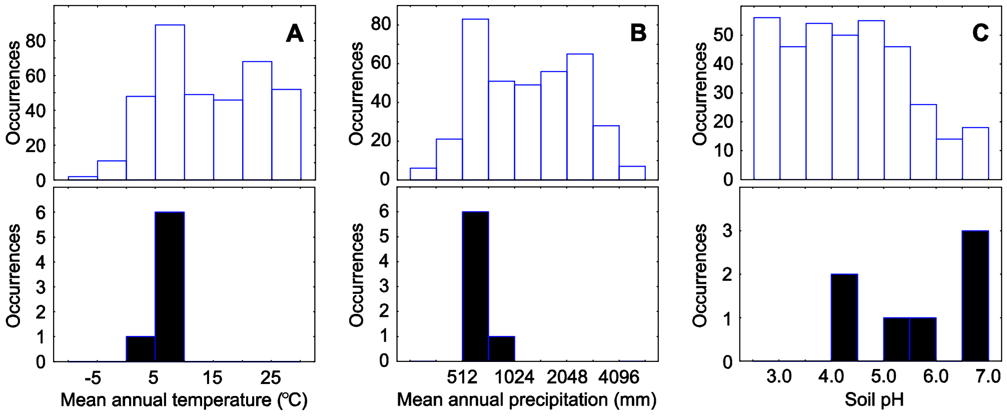

Figure. Niche analysis of the clade. A-C. Distribution of clade members (lower panels) in sites with (A) different mean annual temperature, (B) mean annual precipitation, and (C) average soil pH relative to all studied soils (upper panels).

Clade GS09

Previous names for the clade: -
Corresponding PlutoF compound cluster: -

Representative sequence: UDB014949 (habitat: site S131; VO, Russia; 51.9107 N/ 39.4572; temperate coniferous forest dominated by *Pinus sylvestris*)
Contains other sequences: KF568250

Phylogenetic position: A deeply branching clade within Rozellomycota

Ecological habitat: occurs both in fire-disturbed habitats and unburnt sites; narrow temperature range in cool temperate habitats.
Distribution: all continents of the Northern Hemisphere

Other habitats: temperate forest soil in NC, USA (KF568250)

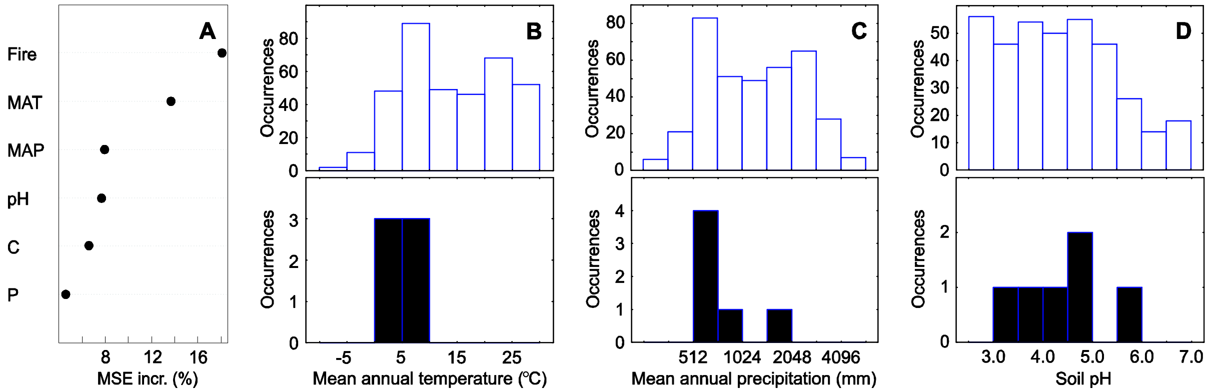

Figure. Niche analysis of the clade. A. Strongest predictors for the occurrence of clade members based on random forest analysis. B-D. Distribution of clade members (lower panels) in sites with (B) different mean annual temperature, (C) mean annual precipitation, and (D) average soil pH relative to all studied soils (upper panels).

Clade GS10

Previous names for the clade: -
Corresponding PlutoF compound cluster: -

Representative sequence: UDB014882 (habitat: site S084; Argentina; 40.1411 S/ 71.4692 W; montane temperate deciduous forest dominated by *Nothofagus obliqua*)
Contains other sequences: UDB014651, KF568205, AF372716, AY916638, AY916644, JN054654, EF024044, EF023474, EU091852

Phylogenetic position: One of the lineages of Rozellomycota

Ecological habitat: the frequency of taxa related to UDB014882 peaks in cool temperate climate and near-neutral soils, whereas OTUs related to UDB014651 are distinctly more common in acidic soils in tropical climate. Other studies suggest frequent occurrence in freshwater. Probably very wide ecological amplitude.
Distribution: both tropical amd cool temperate habitats in both hemispheres

Other habitats: temperate forest soil in USA (KF568205, EF024…), temperate lake sediments in USA and the Netherlands, (AF372716, EU091852), suplhide-rich spring in USA (AY916…), and aerated activated sludge in Australia (JN054654)

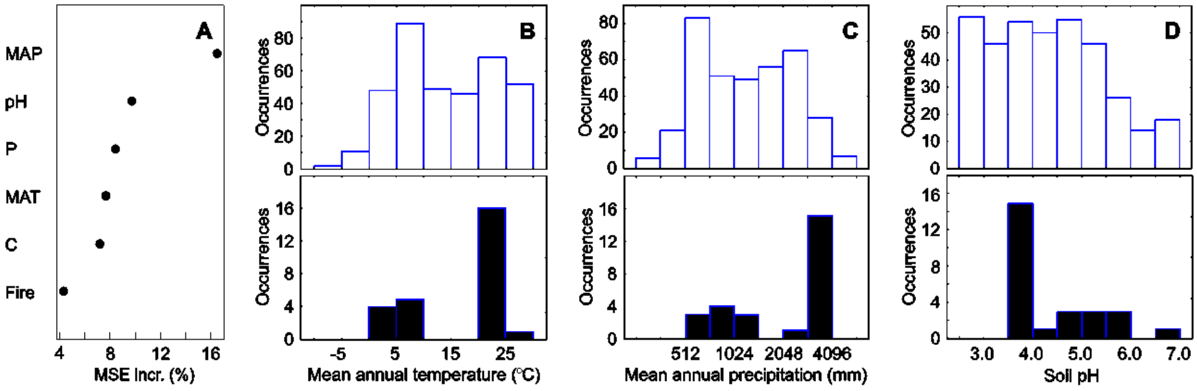

Figure. Niche analysis of the clade. A. Strongest predictors for the occurrence of clade members based on random forest analysis. B-D. Distribution of clade members (lower panels) in sites with (B) different mean annual temperature, (C) mean annual precipitation, and (D) average soil pH relative to all studied soils (upper panels).

Text S1.11

Clade GS11

Previous names for the clade: -
Corresponding PlutoF compound cluster: UCL7_003716, UCL7_007517

Representative sequence: UDB014836 (habitat: site S206; Q, Australia; 28.2733 S/ 153.1724 E; warm temperate forest dominated by *Nothofagus moorei*)
Contains other sequences: UDB014654, UDB014838, UDB014856, UDB014876, AJ506016, AJ506030, EU091829, EU091851, GU568155, HM487051

Phylogenetic position: a compact lineage within Rozellomycota with no long branches

Ecological habitat: widely distributed in all biomes except tundra; especially common in tropical lowland and montane rain forests and temperate deciduous forests; a wide pH range with an optimum at low and medium-low values, but tends to avoid pH<3.
Distribution: world-wide distribution but strikingly less common in southern South America.

Other habitats: amphipod feces (HM487051), temperate agricultural soil (GU568155, AJ506016, AJ506030), and lake sediments (EU091829, EU091851)

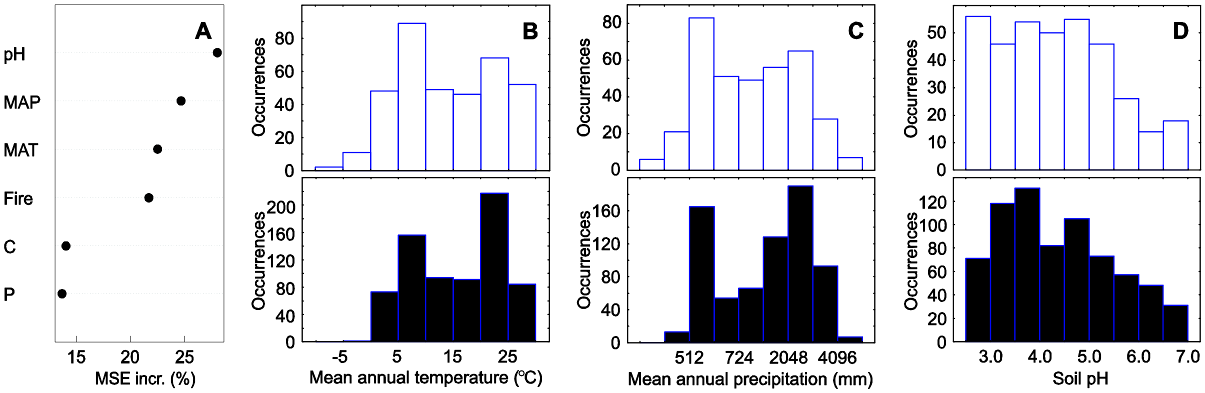

Figure. Niche analysis of the clade. A. Strongest predictors for the occurrence of clade members based on random forest analysis. B-D. Distribution of clade members (lower panels) in sites with (B) different mean annual temperature, (C) mean annual precipitation, and (D) average soil pH relative to all studied soils (upper panels).

Branch2

Previous names for the clade: -
Corresponding PlutoF compound cluster: -

Representative sequence: UDB014923 (habitat: site G2732; Papua New Guinea; 5.9305 S/ 145.0333 W; tropical montane forest dominated by *Lithocarpus* spp.)
Contains other sequences: -

Phylogenetic position: a single long branch within Rozellomycota

Ecological habitat: too rare to evaluate
Distribution: A single rare OTU distributed in Estonia and Papua New Guinea

Other habitats: -

Clade GS12

Previous names for the clade: -
Corresponding PlutoF compound cluster: -

Representative sequence: UDB014881 (habitat: site G2839; Norway; 70.1473 N/ 24.7869 E; the northernmost natural *Pinus sylvestris*-dominated boreal forest)
Contains other sequences: UDB014889, KC965522

Phylogenetic position: One of the lineages of Rozellomycota

Ecological habitat: mostly boreal forest and tundra ecosystems with relatively cool climate and acidic soils
Distribution: mostly northern parts of Europe, Asia, and North America; almost missing in lowland tropical habitats

Other habitats: North American tundra (KC965522)

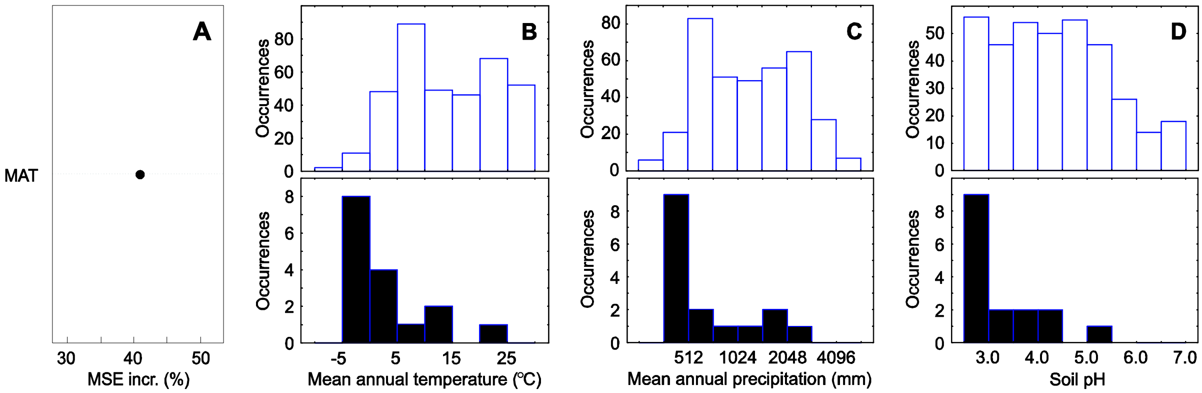

Figure. Niche analysis of the clade. A. Strongest predictors for the occurrence of clade members based on random forest analysis. B-D. Distribution of clade members (lower panels) in sites with (B) different mean annual temperature, (C) mean annual precipitation, and (D) average soil pH relative to all studied soils (upper panels).

Text S1.14

Branch3

Previous names for the clade: -
Corresponding PlutoF compound cluster: -

Representative sequence: UDB014895 (habitat: site G2677; Papua New Guinea; 6.0173 S; 145.4160 E; tropical montane forest dominated by Lauraceae)
Contains other sequences: -

Phylogenetic position: a single long branch within Rozellomycota

Ecological habitat: too rare to evaluate
Distribution: a single OTU found twice in tropical montane forests of New Guinea

Other habitats: -

Clade GS13

Previous names for the clade: -
Corresponding PlutoF compound cluster: -

Representative sequence: UDB014895 (habitat: site G2750; NT, Australia; 13.7655 S/ 131.4395 E; alluvial dry tropical forest dominated by *Melaleuca* sp.)
Contains other sequences: DQ244008, KC558245

Phylogenetic position: one of the lineages within Chytridiomycota *s. lat*.

Ecological habitat: narrow niche for high-temperature over wide pH and precipitation ranges
Distribution: most common in Australian dry tropical forests and savannas

Other habitats: lake water in France (DQ244008); desert soil in ID, USA (KC558245)

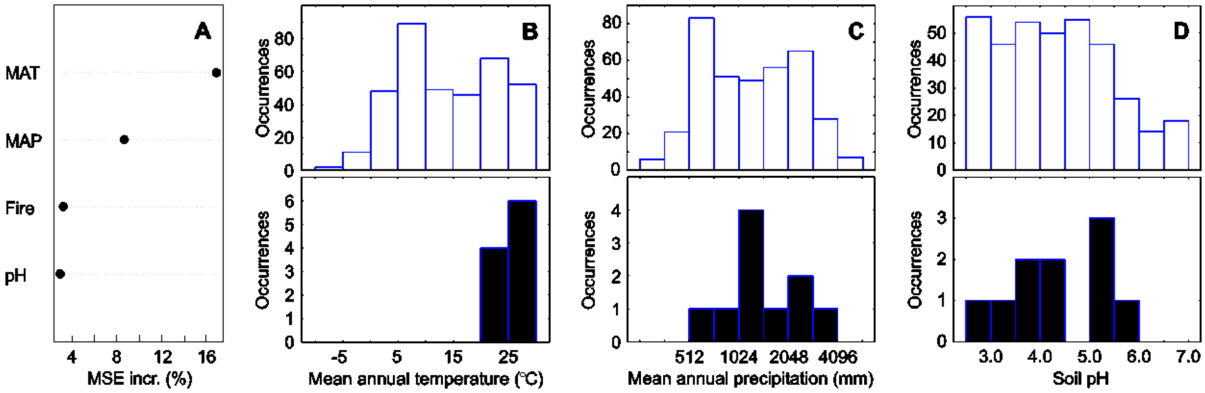

Figure. Niche analysis of the clade. A. Strongest predictors for the occurrence of clade members based on random forest analysis. B-D. Distribution of clade members (lower panels) in sites with (B) different mean annual temperature, (C) mean annual precipitation, and (D) average soil pH relative to all studied soils (upper panels).

Text S1.16

Clade GS14

Previous names for the clade: -
Corresponding PlutoF compound cluster: UCL7_005854

Representative sequence: UDB014658 (habitat: site S002; Madagascar; 24.9514 S/ 47.0001 E; dry tropical forest dominated by *Sarcolaena* spp.)
Contains other sequences: KF566531

Phylogenetic position: A well-supported sister group of Spizellomycetales within Chytridiomycota *s.str.*

Ecological habitat: tends to be more common in relatively warm and wet climate, moderately acid soil; found in all biomes except boreal forests and tundra
Distribution: present in most regions; tends to be more common in Southern Hemisphere

Other habitats: temperate forest soil in NC, USA (KF566531)

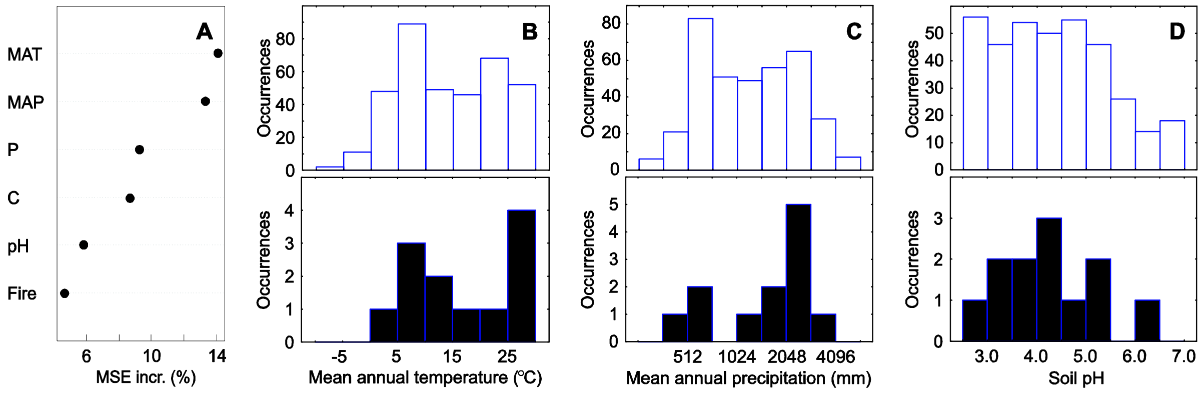

Figure. Niche analysis of the clade. A. Strongest predictors for the occurrence of clade members based on random forest analysis. B-D. Distribution of clade members (lower panels) in sites with (B) different mean annual temperature, (C) mean annual precipitation, and (D) average soil pH relative to all studied soils (upper panels).

Text S1.17

Clade GS15

Previous names for the clade: -
Corresponding PlutoF compound cluster: -

Representative sequence: UDB014619 (habitat: site S238; Finland; 68.9678 N/ 20.9432 E; mountain tundra dominated by dwarf *Betula pubescens*)
Contains other sequences: UDB014635, UDB014631, KF565916

Phylogenetic position: a sister group to Blastocladiales

Ecological habitat: non-tropical biomes; two distinct pH optima at low pH and nearly neutral soils
Distribution: mostly Southern South America but present in Australia, Europe, and Central America

Other habitats: temperate forest soil of NC, USA (KF565916)

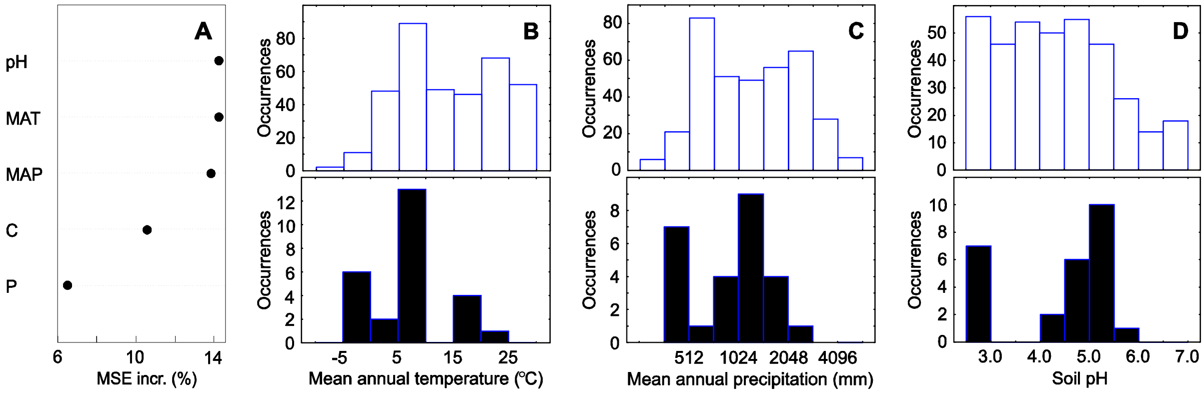

Figure. Niche analysis of the clade. A. Strongest predictors for the occurrence of clade members based on random forest analysis. B-D. Distribution of clade members (lower panels) in sites with (B) different mean annual temperature, (C) mean annual precipitation, and (D) average soil pH relative to all studied soils (upper panels).

Text S1.18

Clade GS16

Previous names for the clade: -
Corresponding PlutoF compound cluster: -

Representative sequence: UDB014729 (habitat: site S188; BJ, China; 42.3841 N/ 128.0935 E; montane temperate deciduous forest dominated by *Quercus mongolica*)
Contains other sequences: UDB014777, UDB014731, UDB014732, HM487005, AY180024, AB252775, GQ995421, GQ995419, GQ995418, FJ157332, AM114806, JX534788

Phylogenetic position: nested within Aphelidea forming a monophyletic group including multiple sequences of uncultured fungi

Ecological habitat: mostly temperate soils with high C content; wide pH amplitude
Distribution: present in most ecoregions with no trends; very wide ecological amplitude based on other studies

Other habitats: amphipod feces (HM487005), freshwater (FJ157332), marine sediments (AY180024), salt lake sediments (AB252775), tundra soil of Co, USA and/or Nepal (GQ995…), temperate agricultural soil in the Netherlands and China (AM114806, JX534788)

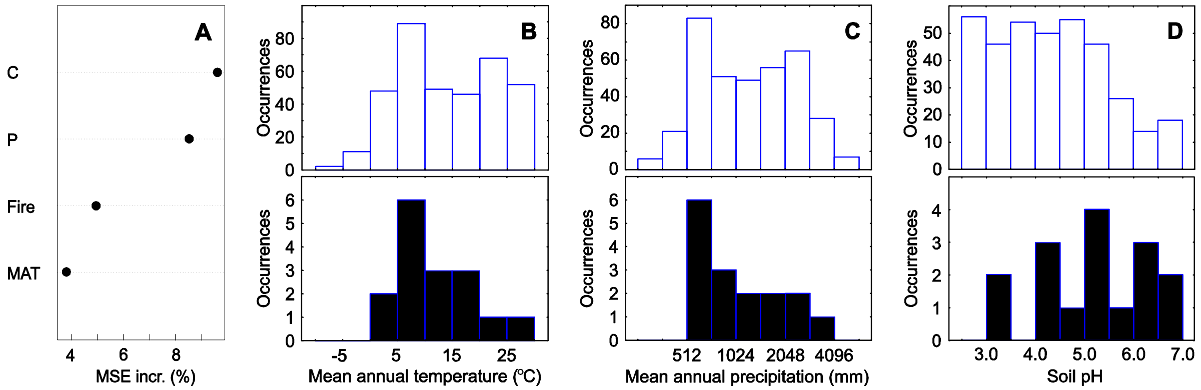

Figure. Niche analysis of the clade. A. Strongest predictors for the occurrence of clade members based on random forest analysis. B-D. Distribution of clade members (lower panels) in sites with (B) different mean annual temperature, (C) mean annual precipitation, and (D) average soil pH relative to all studied soils (upper panels).

Text S1.19

Clade GS17

Previous names for the clade: -
Corresponding PlutoF compound cluster: -

Representative sequence: UDB014847 (habitat: site S124; Italy; 37.8747 N/ 14.0158 E; montane temperate deciduous forest dominated by *Fagus sylvatica*)
Contains other sequences: KC671706

Phylogenetic position: one of the two well-supported sister lineages to Olpidiales

Ecological habitat: temperate forest soils with moderate precipitation and weakly acidic pH
Distribution: known only from the Northern Hemisphere

Other habitats: human skin (KC671706)

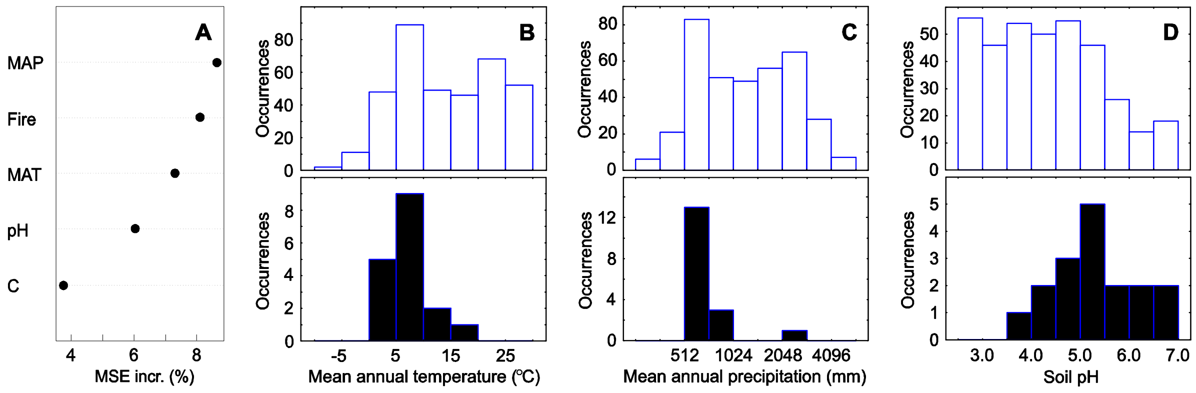

Figure. Niche analysis of the clade. A. Strongest predictors for the occurrence of clade members based on random forest analysis. B-D. Distribution of clade members (lower panels) in sites with (B) different mean annual temperature, (C) mean annual precipitation, and (D) average soil pH relative to all studied soils (upper panels).

Clade GS18

Previous names for the clade: -
Corresponding PlutoF compound cluster: UCL7_008108

Representative sequence: UDB014671 (habitat: site G2835; Sweden; 68.2259 N/ 21.9452 E; northern boreal forest dominated by *Pinus sylvestris*)
Contains other sequences: UDB014615, UDB014640, UDB014641, UDB014647, UDB014671, KF567173

Phylogenetic position: one of the two well-supported sister lineages to Olpidiales

Ecological habitat: prefers boreal and temperate coniferous forests with moderate precipitation and strongly to moderately acidic pH
Distribution: by far most common in temperate Eurasia

Other habitats: temperate forest soils in NC, USA (KF567173)

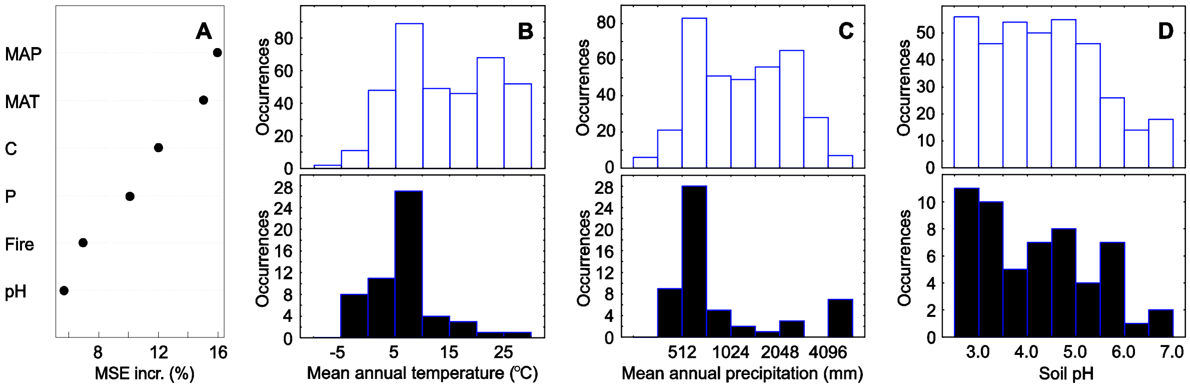

Figure. Niche analysis of the clade. A. Strongest predictors for the occurrence of clade members based on random forest analysis. B-D. Distribution of clade members (lower panels) in sites with (B) different mean annual temperature, (C) mean annual precipitation, and (D) average soil pH relative to all studied soils (upper panels).

Clade GS19

Previous names for the clade: -
Corresponding PlutoF compound cluster: UCL7_009149

Representative sequence: UDB014747 (habitat: site S008; Madagascar; 22.5615 S/ 45.3810 E; dry subtropical savanna dominated by *Sarcolaena* spp.)
Contains other sequences: UDB014614, UDB014678, GU055690

Phylogenetic position: An isolated lineage among zygomycetes *s.lat*.

Ecological habitat: preferential occurrence in humid tropical climate (especially tropical rain forests and dry forests) at low soil pH
Distribution: found in nearly all ecoregions; particularly common in SE Asia.

Other habitats: agricultural soil in Austria (GU055690)

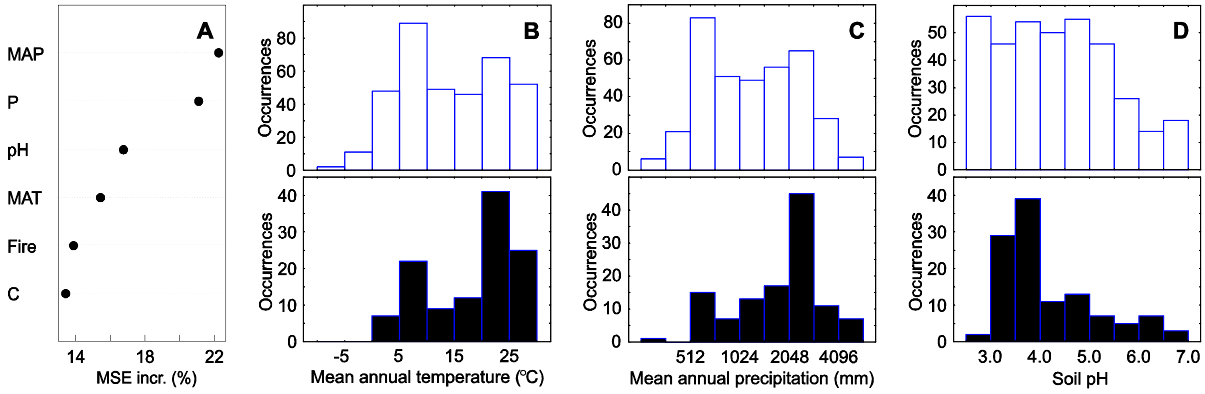

Figure. Niche analysis of the clade. A. Strongest predictors for the occurrence of clade members based on random forest analysis. B-D. Distribution of clade members (lower panels) in sites with (B) different mean annual temperature, (C) mean annual precipitation, and (D) average soil pH relative to all studied soils (upper panels).

Text S1.22

Clade GS20

Previous names for the clade: -
Corresponding PlutoF compound cluster: UCL7_006587

Representative sequence: UDB014697 (habitat: site G2660; Papua New Guinea; 9.4218 S/ 147.3692 E; tropical lowland rain forest with no dominant trees)
Contains other sequences: UDB014694, KF565030
Phylogenetic position: one of the basal lineages of Mucoromycotina, with unsupported relationship to Endogonales

Ecological habitat: widely distributed especially in tropical climate (rain forest and savanna habitats) in strongly to moderately acidic soils with relatively low P concentration.
Distribution: found in all regions, except New Zealand

Other habitats: temperate forest soil in NC, USA (KF565030)

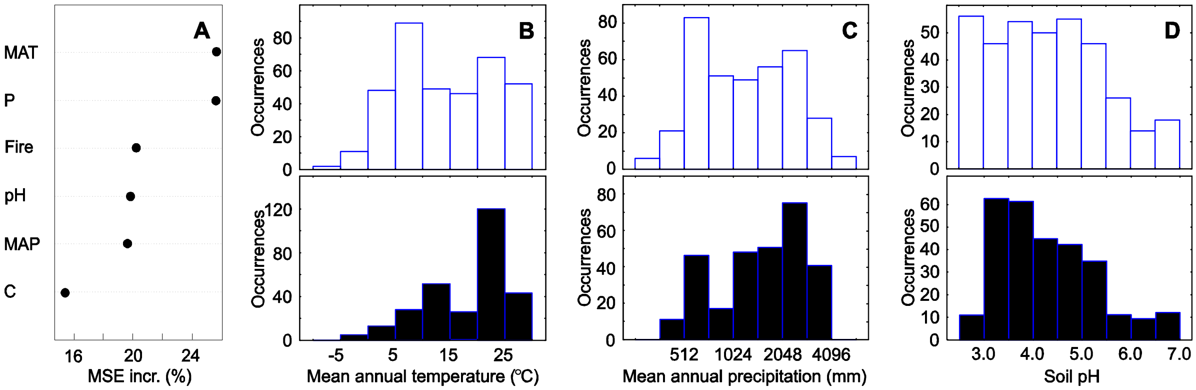

Figure. Niche analysis of the clade. A. Strongest predictors for the occurrence of clade members based on random forest analysis. B-D. Distribution of clade members (lower panels) in sites with (B) different mean annual temperature, (C) mean annual precipitation, and (D) average soil pH relative to all studied soils (upper panels).

Clade GS21

Previous names for the clade: -
Corresponding PlutoF compound cluster: -

Representative sequence: UDB014852 (habitat: site S049; Puerto Rico; 18.3210 N/ 65.8197 W; tropical lowland rain forest dominated by *Coccoloba swartzii*)
Contains other sequences: UDB014810, GU055679, KF565318

Phylogenetic position: one of the basal lineages of Mucoromycotina, with unsupported relationship to Endogonales

Ecological habitat: only in tropical rain forest and savanna habitats in soils with medium P and C concentrations and low pH
Distribution: present in tropical ecosystems of all continents. Other studies suggest wider distribution in temperate soils.

Other habitats: temperate forest soil in NC, USA (KF565318), agricultural soil in Austria (GU055679)

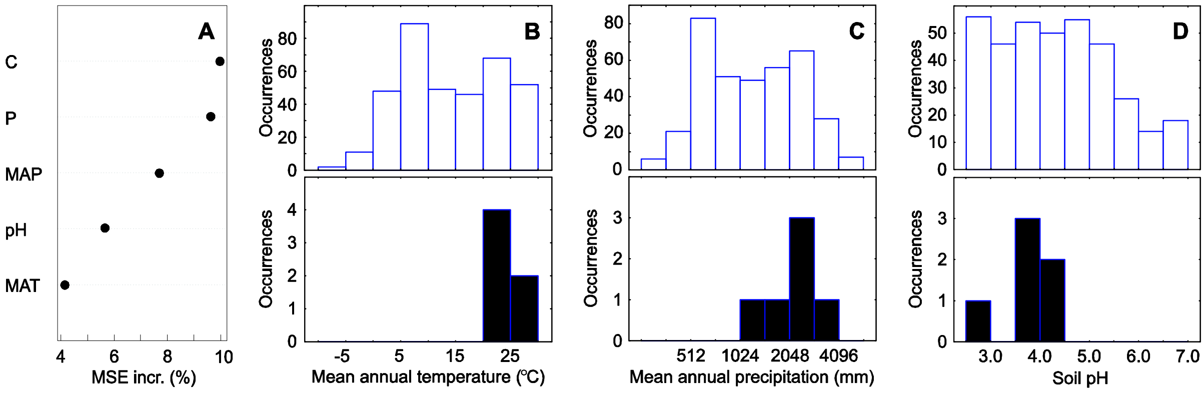

Figure. Niche analysis of the clade. A. Strongest predictors for the occurrence of clade members based on random forest analysis. B-D. Distribution of clade members (lower panels) in sites with (B) different mean annual temperature, (C) mean annual precipitation, and (D) average soil pH relative to all studied soils (upper panels).

Text S1.24

Clade GS22

Previous names for the clade: -
Corresponding PlutoF compound cluster: -

Representative sequence: UDB014740 (habitat: site S171; New Zealand; 42.3024 S/ 172.1041 E; montane temperate deciduous forest dominated by *Nothofagus menziesii*)
Contains other sequences: KF566241

Phylogenetic position: one of the basal lineages of Mucoromycotina, with unsupported relationship to Endogonales

Ecological habitat: especially common in boreal forests of Eurasia in highly acidic soils.
Distribution: widespread in non-tropical habitats, especially New Zealand; also relatively common in Eurasia.

Other habitats: temperate forest soil in NC, USA (KF566241)

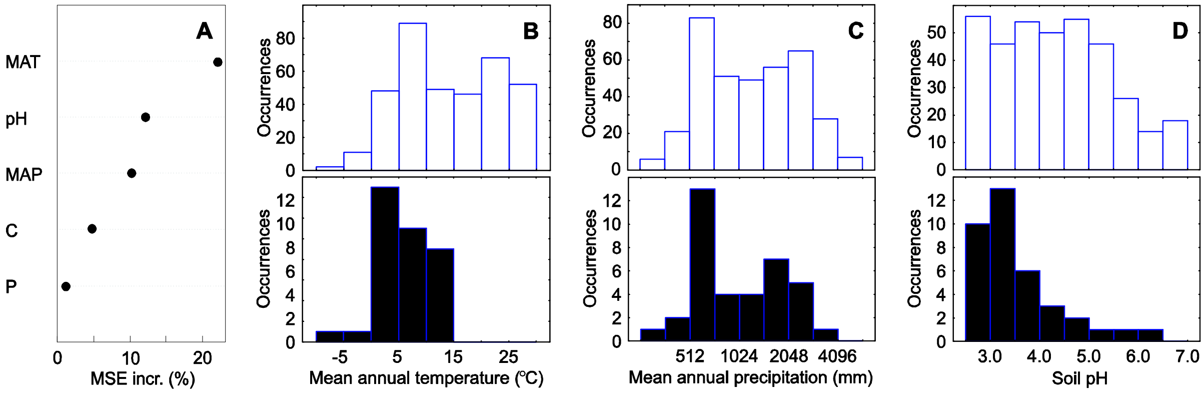

Figure. Niche analysis of the clade. A. Strongest predictors for the occurrence of clade members based on random forest analysis. B-D. Distribution of clade members (lower panels) in sites with (B) different mean annual temperature, (C) mean annual precipitation, and (D) average soil pH relative to all studied soils (upper panels).

Text S1.25

Clade GS23

Previous names for the clade: -
Corresponding PlutoF compound cluster: -

Representative sequence: UDB014792 (habitat: site G2643; WA, Australia; 34.9522 S/ 116.7588 E; Mediterranean woodland dominated by *Eucalyptus jacksonii*)
Contains other sequences: UDB014874, UDB014840, UDB014805, UDB014806

Phylogenetic position: well-supported sister group to the Umbelopsidaceae

Ecological habitat: tropical and subtropical with high humidity; in soils with low pH
Distribution: present in all continents but especially common in SE Asia

Other habitats: -

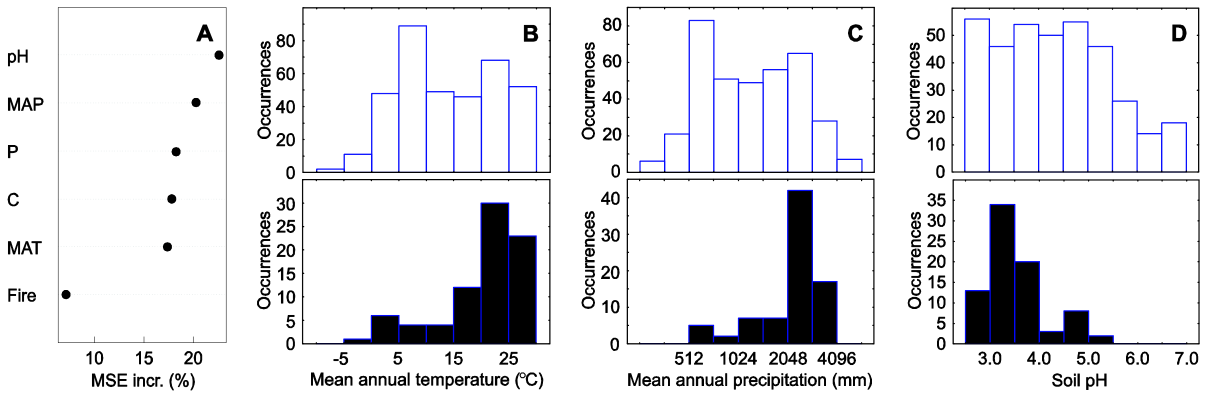

Figure. Niche analysis of the clade. A. Strongest predictors for the occurrence of clade members based on random forest analysis. B-D. Distribution of clade members (lower panels) in sites with (B) different mean annual temperature, (C) mean annual precipitation, and (D) average soil pH relative to all studied soils (upper panels).

Text S1.26

Clade GS24

Previous names for the clade: -
Corresponding PlutoF compound cluster: -

Representative sequence: UDB014833 (habitat: site S045; Puerto Rico; 18.3167 N/ 65.8167 W; tropical lowland rain forest with no clear dominant trees)
Contains other sequences: UDB014749, JN936327

Phylogenetic position: a group with a sister position to Paraglomales within Glomeromycota

Ecological habitat: mostly tropical soils with moderately acidic to neutral pH; most common in the dry tropical forests biome.
Distribution: several tropical ecoregions; particularly common in Central America and New Guinea.

Other habitats: desert soil in Oman (JN936327)

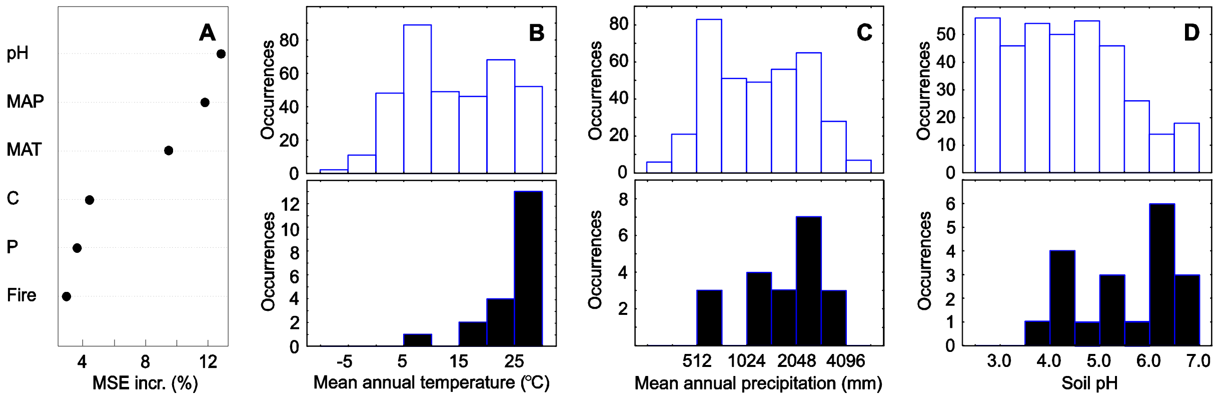

Figure. Niche analysis of the clade. A. Strongest predictors for the occurrence of clade members based on random forest analysis. B-D. Distribution of clade members (lower panels) in sites with (B) different mean annual temperature, (C) mean annual precipitation, and (D) average soil pH relative to all studied soils (upper panels).

Text S1.27

Branch4

Previous names for the clade: -
Corresponding PlutoF compound cluster: -

Representative sequence: UDB014934 (habitat: site G2745; NT, Australia; 12.7188 S/ 131.7455 E; dense savanna dominated by *Eucalyptus* spp.)
Contains other sequences: -

Phylogenetic position: a single lineage with a strongly supported position between Entorrhizomycetes and Talbotiomycetes within the Entorrhizomycota

Ecological habitat: tends to be more common in tropical habitats; found in moderately acidic soils
Distribution: most often found in *Eucalyptus* woodlands of the Northern Territory, Australia, with single occurrences in New Guinea, Southern South America, and Europe

Other habitats: -

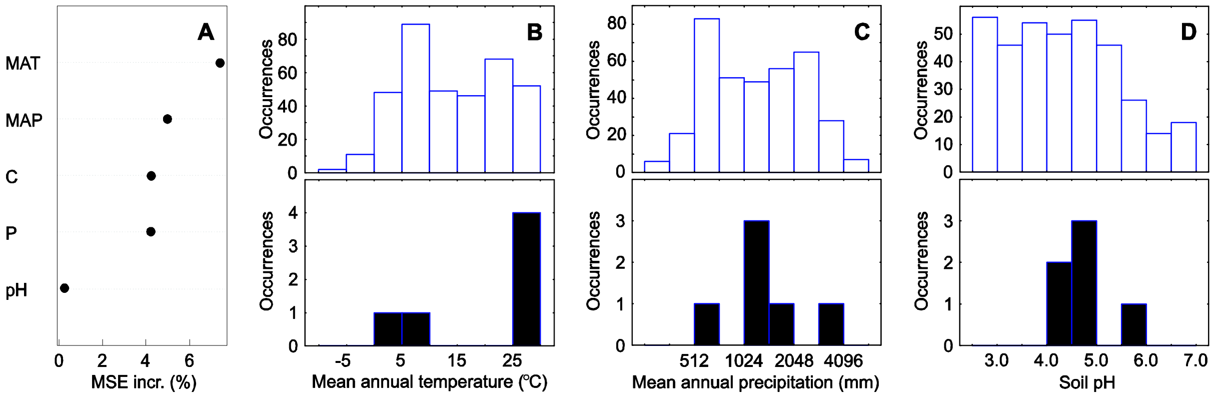

Figure. Niche analysis of the clade. A. Strongest predictors for the occurrence of clade members based on random forest analysis. B-D. Distribution of clade members (lower panels) in sites with (B) different mean annual temperature, (C) mean annual precipitation, and (D) average soil pH relative to all studied soils (upper panels).

Clade GS25

Previous names for the clade: -
Corresponding PlutoF compound cluster: -

Representative sequence: UDB014764 (habitat: site S159; NF, Canada; 48.9895 N/ 57.7870 W; temperate deciduous forest dominated by *Betula alleghanensis*)
Contains other sequences: UDB014726, EU522908, EF023636

Phylogenetic position: one of the early branching lineages of Pucciniomycotina within Basidiomycota

Ecological habitat: most common in temperate habitats and moderately acidic soils
Distribution: found in most continents with no evident biogeographic patterns

Other habitats: temperate forest soil in ON, Canada (EU522908) and WI, USA (EF023636)

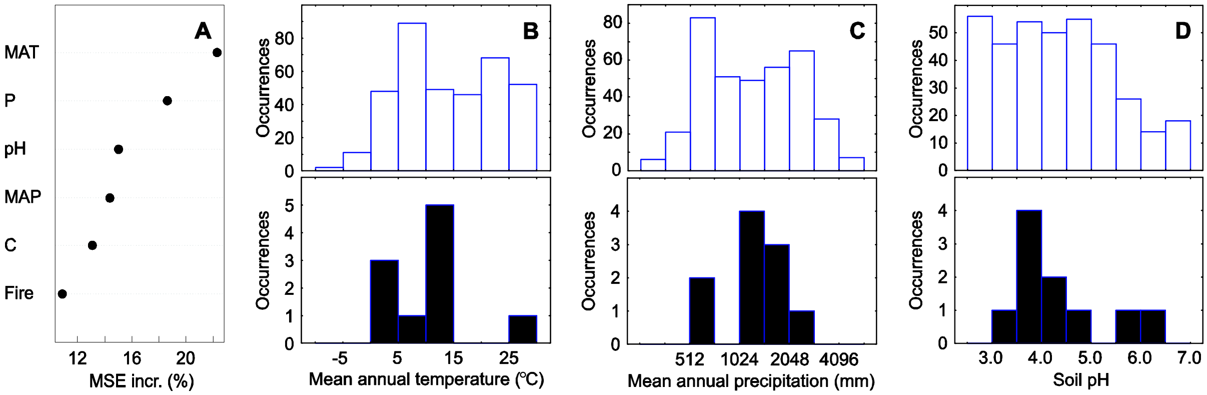

Figure. Niche analysis of the clade. A. Strongest predictors for the occurrence of clade members based on random forest analysis. B-D. Distribution of clade members (lower panels) in sites with (B) different mean annual temperature, (C) mean annual precipitation, and (D) average soil pH relative to all studied soils (upper panels).

Text S1.29

Clade GS26

Previous names for the clade: -
Corresponding PlutoF compound cluster: -

Representative sequence: UDB014723 (habitat: site S060; Guyana; 5.2795 N/ -59.9159; tropical lowland rain forest dominanted by *Dicymbe corymbosa*)
Contains other sequences: UDB014713, UDB014717

Phylogenetic position: one of the early branching lineages of Pucciniomycotina within Basidiomycota

Ecological habitat: extremely acidic tropical rain forest soils
Distribution: found only in the Roraima massif of Guyana

Other habitats: -

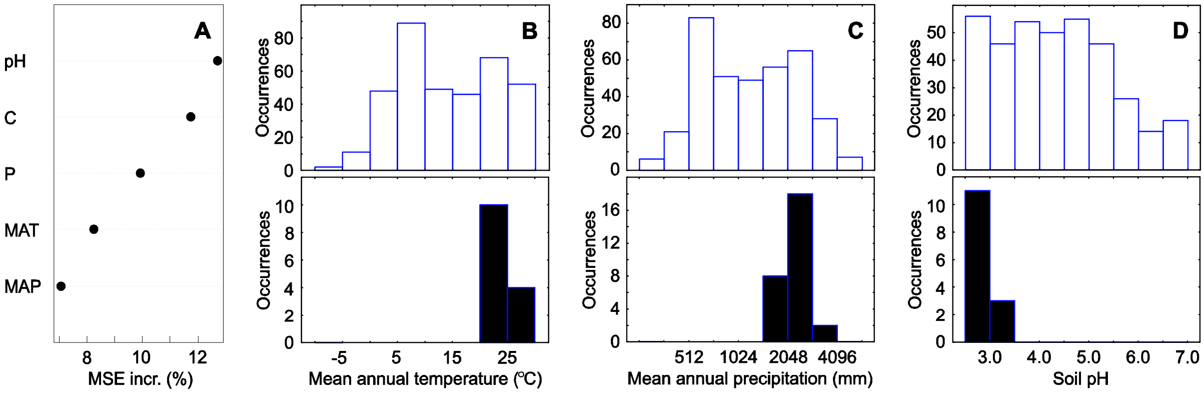

Figure. Niche analysis of the clade. A. Strongest predictors for the occurrence of clade members based on random forest analysis. B-D. Distribution of clade members (lower panels) in sites with (B) different mean annual temperature, (C) mean annual precipitation, and (D) average soil pH relative to all studied soils (upper panels).

Clade GS27

Previous names for the clade: -
Corresponding PlutoF compound cluster: -

Representative sequence: UDB014864 (habitat: site S114; Czech Republic; 49.0174 N/ 13.4751 W; montane temperate coniferous forest dominated by *Picea abies*)
Contains other sequences: UDB014861, UDB014624, UDB014761, UDB014779, DQ198784, KF565271

Phylogenetic position: related to Agaricostilbomycetes and Tritirachiomycetes within Pucciniomycotina (Basidiomycota); contains two coherent lineages, one of which is represented by a voucher specimen RB1040 and tentatively (mis)named as *Platygloea*

Ecological habitat: prefers cool temperate climate (especially boreal forests and temperate deciduous forests) and weakly acidic soils; wide tolerance to extreme values of soil pH
Distribution: present in all continents; common in the Northern and Southern Hemisphere

Other habitats: Other habitats: temperate forest soil in NC, USA; source of the culture RB1040 is unknown

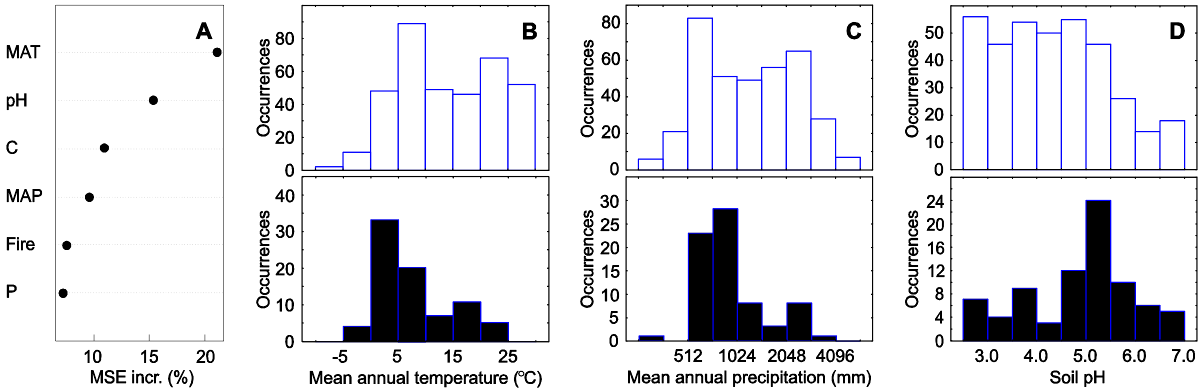

Figure. Niche analysis of the clade. A. Strongest predictors for the occurrence of clade members based on random forest analysis. B-D. Distribution of clade members (lower panels) in sites with (B) different mean annual temperature, (C) mean annual precipitation, and (D) average soil pH relative to all studied soils (upper panels).

Clade GS28

Previous names for the clade: -
Corresponding PlutoF compound cluster: -

Representative sequence: UDB014693 (habitat: site S004; Madagascar; 47.0020 S/ 24.9520 E; dry deciduous forest dominated by *Uapaca* spp.)
Contains other sequences: UDB014783, UDB014691, UDB014682, UDB014716

Phylogenetic position: a long but well-supported branch related to Sebacinales and Cantharellales at the base of Agaricomycetes (Basidiomycota)

Ecological habitat: wet tropical climate (rain forest and dry forest biomes); tends to prefer highly acidic soils
Distribution: pantropical distribution but particularly common in northern South America and Madagascar

Other habitats: -

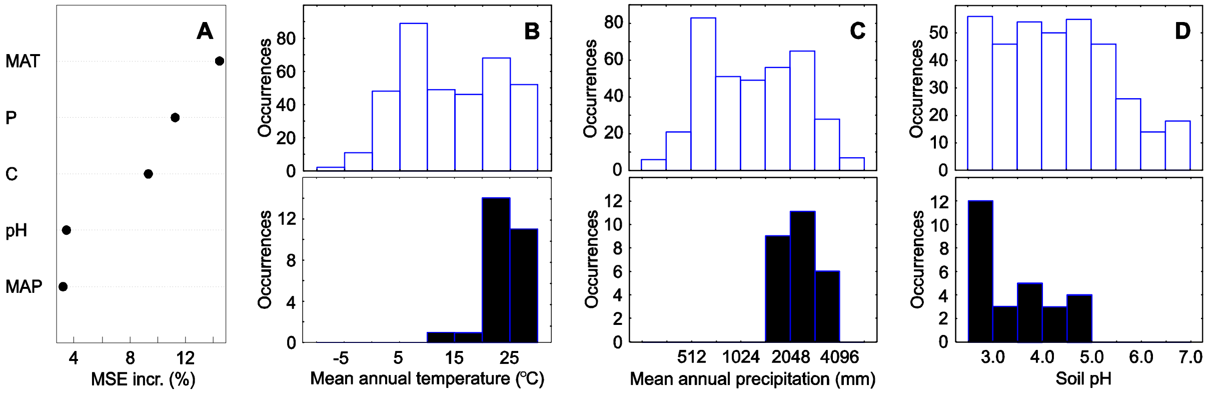

Figure. Niche analysis of the clade. A. Strongest predictors for the occurrence of clade members based on random forest analysis. B-D. Distribution of clade members (lower panels) in sites with (B) different mean annual temperature, (C) mean annual precipitation, and (D) average soil pH relative to all studied soils (upper panels).

Text S1.32

Branch5

Previous names for the clade: -
Corresponding PlutoF compound cluster: -

Representative sequence: UDB014858 (habitat: site G2647; WA, Australia; 34.9543 S/ 116.8395 E; Mediterranean sclerophyll forest dominated by *Eucalyptus jacksonii*)
Contains other sequences: -

Phylogenetic position: unsettled position in the Phallomycetidae within Basidiomycota; it may represent an early diverging group of Hysterangiales

Ecological habitat: moist tropical forests and savannas of Queensland and West Australia; Relatively more common in sites with recent fire (1-2 years prior to sampling)
Distribution: only known from Queensland and West Australia

Other habitats: -

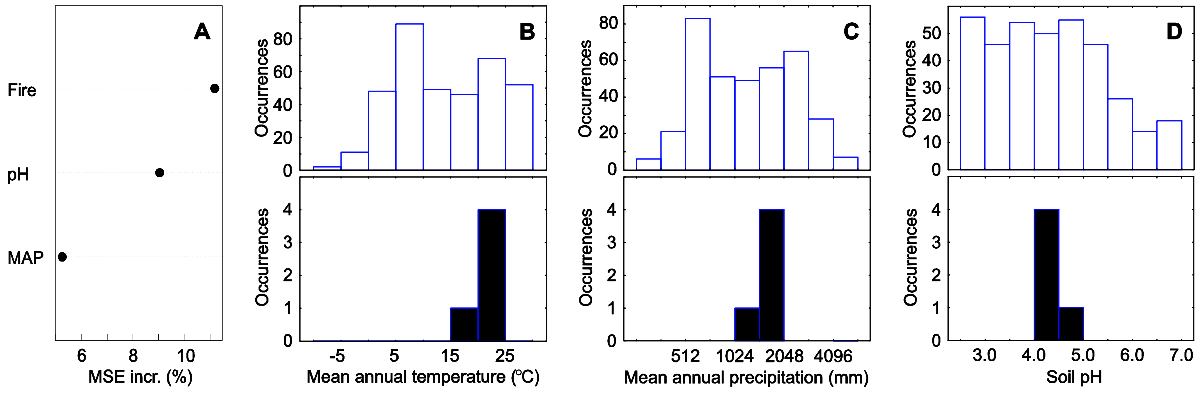

Figure. Niche analysis of the clade. A. Strongest predictors for the occurrence of clade members based on random forest analysis. B-D. Distribution of clade members (lower panels) in sites with (B) different mean annual temperature, (C) mean annual precipitation, and (D) average soil pH relative to all studied soils (upper panels).

Clade GS29

Previous names for the clade: -
Corresponding PlutoF compound cluster: UCL7_006369

Representative sequence: UDB014802 (habitat: site AV123; Colombia; 4.8670 N/ 75.2500 W; paramo montane grassland)
Contains other sequences: EU861637

Phylogenetic position: sister group of Auriculariales within Basidiomycota

Ecological habitat: prefers wet tropical climate and low pH
Distribution: found only in the Southern Hemisphere (New Guinea and northern South America), but other studies indicate much broader distribution and climate tolerance

Other habitats: grassland soil in CO, USA (EU861637)

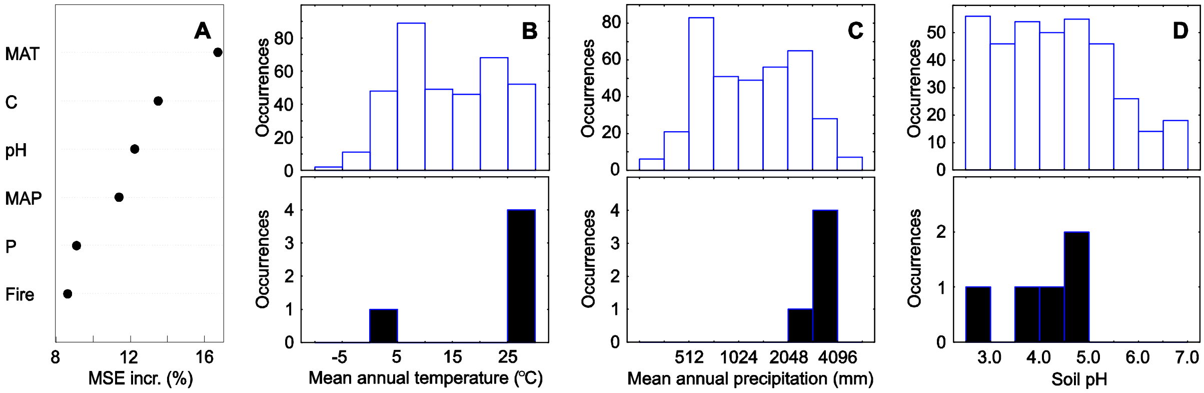

Figure. Niche analysis of the clade. A. Strongest predictors for the occurrence of clade members based on random forest analysis. B-D. Distribution of clade members (lower panels) in sites with (B) different mean annual temperature, (C) mean annual precipitation, and (D) average soil pH relative to all studied soils (upper panels).

Clade GS30

Previous names for the clade: covers the ectomycorrhizal lineage /agaricomycetes1 and its potentially non-mycorrhizal sister group (Tedersoo & Smith 2013; Fung. Biol. Rev. 27: 83-99.)
Corresponding PlutoF compound cluster: UCL7_008472

Representative sequence: UDB014766, (habitat: site G2641; WA, Australia; 34.3629 S/ 116.2800 E; Mediterranean forest dominated by *Eucalyptus patens*)
Contains other sequences: UDB014827, UDB014829, UDB014429

Phylogenetic position: related to Phallomycetidae within Basidiomycota

Ecological habitat: includes both the ectomycorrhizal and non-mycorrhizal members; temperate habitats in the southern and northern temperate deciduous forests and Mediterranean biome
Distribution: strikingly most common in the temperate habitats in southern South America, New Zealand, and Australia; less common in temperate habitats in North America and Europe

Other habitats: ectomycorrhizal root tips of *Nothofagus* in Argentina (UDB014429)

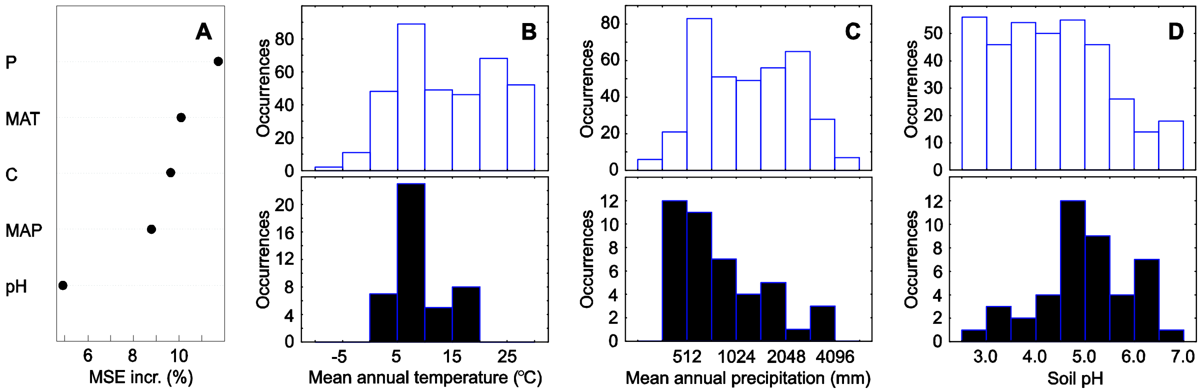

Figure. Niche analysis of the clade. A. Strongest predictors for the occurrence of clade members based on random forest analysis. B-D. Distribution of clade members (lower panels) in sites with (B) different mean annual temperature, (C) mean annual precipitation, and (D) average soil pH relative to all studied soils (upper panels).

Text S1.35

Clade GS31

Previous names for the clade: -
Corresponding PlutoF compound cluster: -

Representative sequence: UDB014859 (habitat: site S046; Puerto Rico; 18.3167 N/ 65.8167 W; tropical lowland rain forest with no clear dominants)
Contains other sequences: UDB014768, UDB014843, UDB014875, UDB014884

Phylogenetic position: well-supported sister group of the Archaeorhizomycetales of Taphrinomycotina (Ascomycota)

Ecological habitat: particularly common in humid tropical climate, especially rain forest and dry forest biomes; prefers soil with low pH
Distribution: found in nearly all ecoregions but strikingly more common in Central America

Other habitats: -

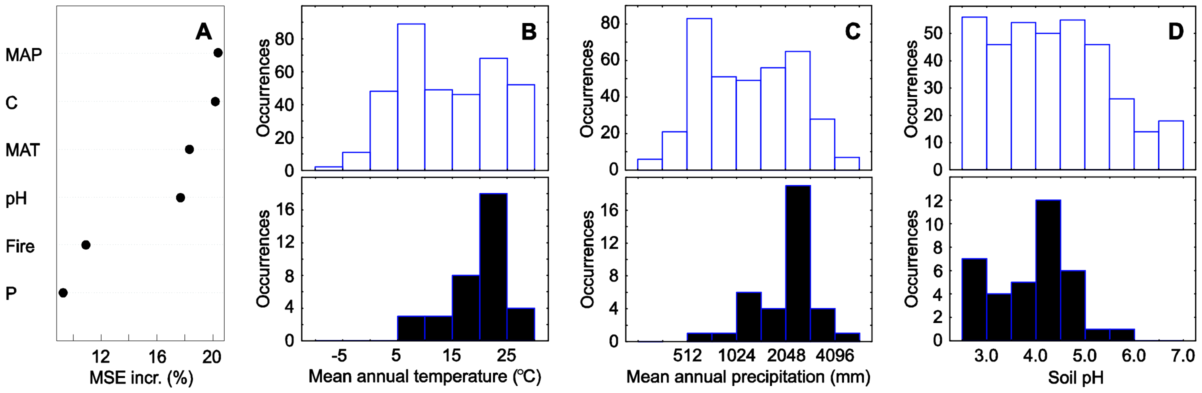

Figure. Niche analysis of the clade. A. Strongest predictors for the occurrence of clade members based on random forest analysis. B-D. Distribution of clade members (lower panels) in sites with (B) different mean annual temperature, (C) mean annual precipitation, and (D) average soil pH relative to all studied soils (upper panels).

Text S1.36

Clade GS32

Previous names for the clade: -
Corresponding PlutoF compound cluster: -

Representative sequence: UDB014870 (habitat: site G2660; Papua New Guinea; 9.4218 S/ 147.3692 E; moderately disturbed tropical savanna dominated by *Eucalyptus papuana*)
Contains other sequences: UDB014869

Phylogenetic position: one of the deep order-level lineages of Orbiliomycetes at the base of Pezizomycotina within Ascomycota

Ecological habitat: humid tropical climate (lowland rain forest) over a wide pH range
Distribution: Central America, with a single locality in New Guinea

Other habitats: -

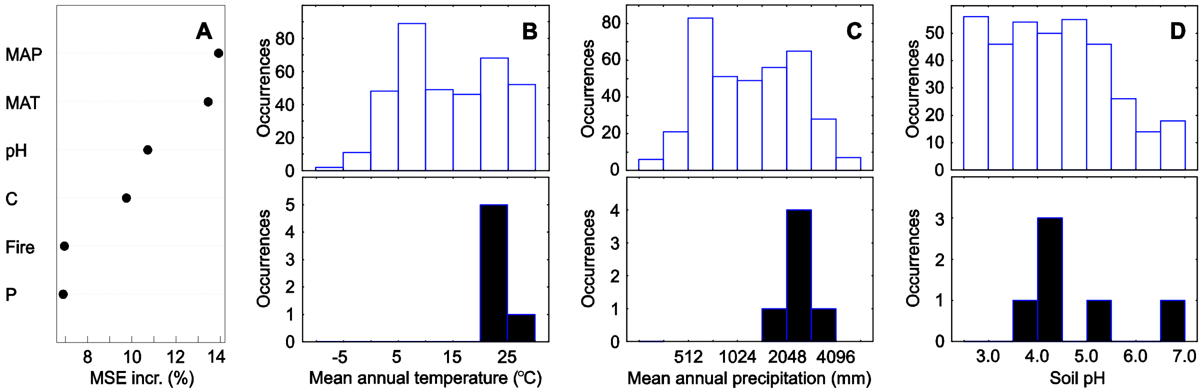

Figure. Niche analysis of the clade. A. Strongest predictors for the occurrence of clade members based on random forest analysis. B-D. Distribution of clade members (lower panels) in sites with (B) different mean annual temperature, (C) mean annual precipitation, and (D) average soil pH relative to all studied soils (upper panels).

Text S1.37

Clade GS33

Previous names for the clade: -
Corresponding PlutoF compound cluster: -

Representative sequence: UDB014886 (habitat: site S049; Puerto Rico; 18.3210 N/ 65.8197 W; tropical lowland rain forest dominated by *Coccoloba swartzii*)
Contains other sequences: UDB014844, UDB014906, UDB014896, UDB014888, KF566789

Phylogenetic position: one of the deep order-level lineages of Orbiliomycetes within Ascomycota

Ecological habitat: more common in tropical moderately acidic soils; most common in the savanna biome
Distribution: found in most ecoregions but most common in Australia, Central America, and Madagascar

Other habitats: temperate forest soil in NC, USA (KF566789)

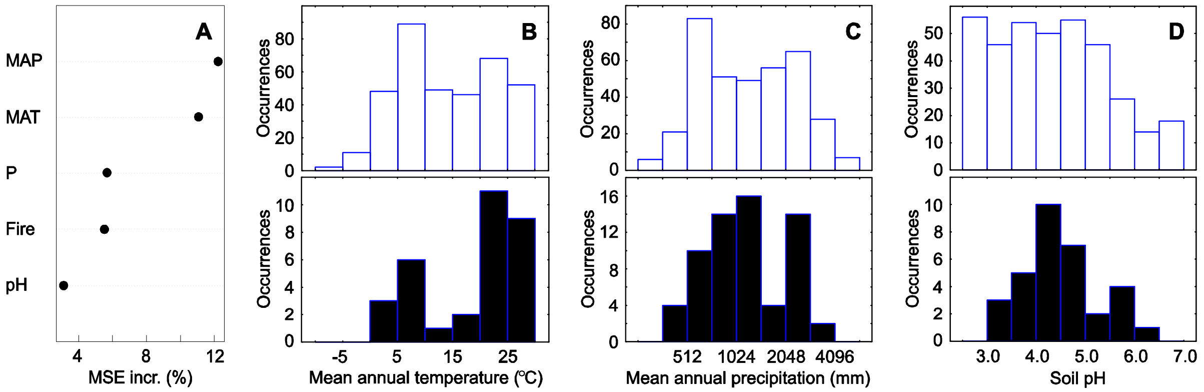

Figure. Niche analysis of the clade. A. Strongest predictors for the occurrence of clade members based on random forest analysis. B-D. Distribution of clade members (lower panels) in sites with (B) different mean annual temperature, (C) mean annual precipitation, and (D) average soil pH relative to all studied soils (upper panels).

Clade GS34

Previous names for the clade: -
Corresponding PlutoF compound cluster: -

Representative sequence: UDB014912 (habitat: site G2629; Estonia; 58.0458 N/ 24.7119 E; old-growth temperate coniferous forest dominated by *Picea abies*)
Contains other sequences: UDB014911, KF565042

Phylogenetic position: related to Symbiotaphrinales within Ascomycota, albeit with low support

Ecological habitat: particularly common in temperate habitats, especially grasslands and shrublands
Distribution: found in all continents but most common in North America

Other habitats: temperate forest soil in NC, USA (KF566789)

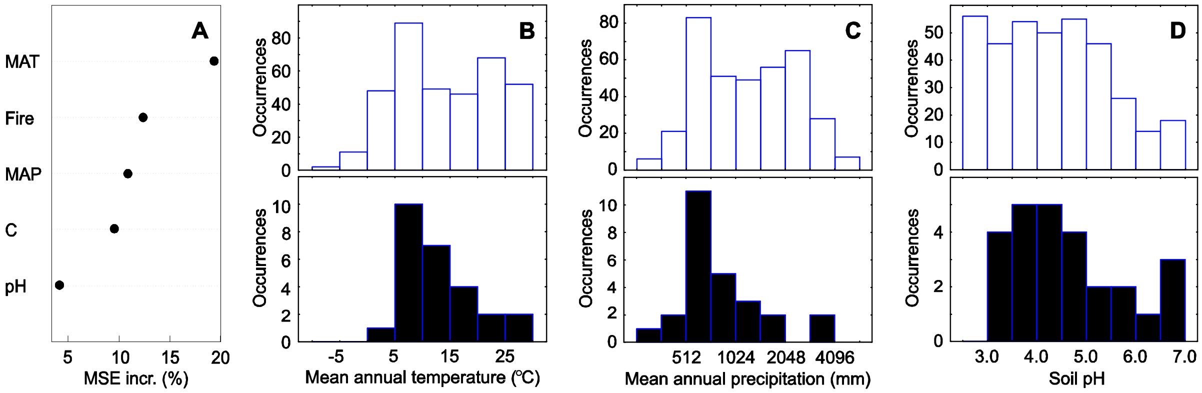

Figure. Niche analysis of the clade. A. Strongest predictors for the occurrence of clade members based on random forest analysis. B-D. Distribution of clade members (lower panels) in sites with (B) different mean annual temperature, (C) mean annual precipitation, and (D) average soil pH relative to all studied soils (upper panels).

Text S1.39

Clade GS35

Previous names for the clade: -
Corresponding PlutoF compound cluster: -

Representative sequence: UDB014945 (habitat: site S163; Mexico; 19.1697 N/ 97.3000 W; montane subalpine coniferous forest dominated by *Pseudotsuga menziesii*)
Contains other sequences: UDB014907, UDB014919, UDB014898, EF023999, KM096534

Phylogenetic position: a clade related to Leotiomycetes and Sordariomycetes, albeit with low support

Ecological habitat: very broad ecological amplitude regarding climate and soil pH; most common in grasslands and shrublands
Distribution: present in all continents and ecoregions without obvious differences.

Other habitats: temperate forest soils in WI, USA (EF023999), and uranium mine in Scotland (KM096534)

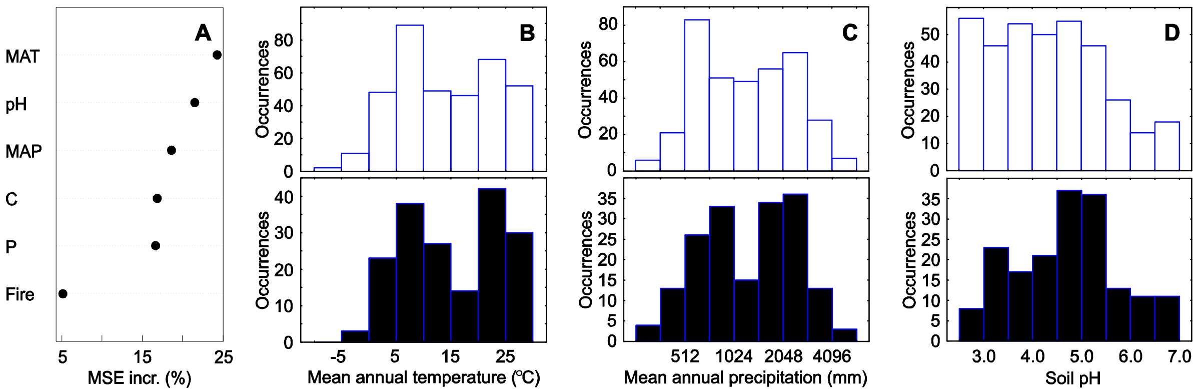

Figure. Niche analysis of the clade. A. Strongest predictors for the occurrence of clade members based on random forest analysis. B-D. Distribution of clade members (lower panels) in sites with (B) different mean annual temperature, (C) mean annual precipitation, and (D) average soil pH relative to all studied soils (upper panels).

Branch6

Previous names for the clade: -
Corresponding PlutoF compound cluster: -

Representative sequence: UDB014790 (habitat: site G2658; Papua New Guinea; 9.4350 S; 147.3755 E; burnt tropical savanna dominated by *Eucalyptus tereticornis*)
Contains other sequences: -

Phylogenetic position: a sister group to the rest of Lulworthiales within Sordariomycetes, Ascomycota

Ecological habitat: tropical dry forests but also savannas and lowland rain forests in soils of moderately high pH
Distribution: most common in Central America, Africa, Papua New Guinea, and India

Other habitats: -

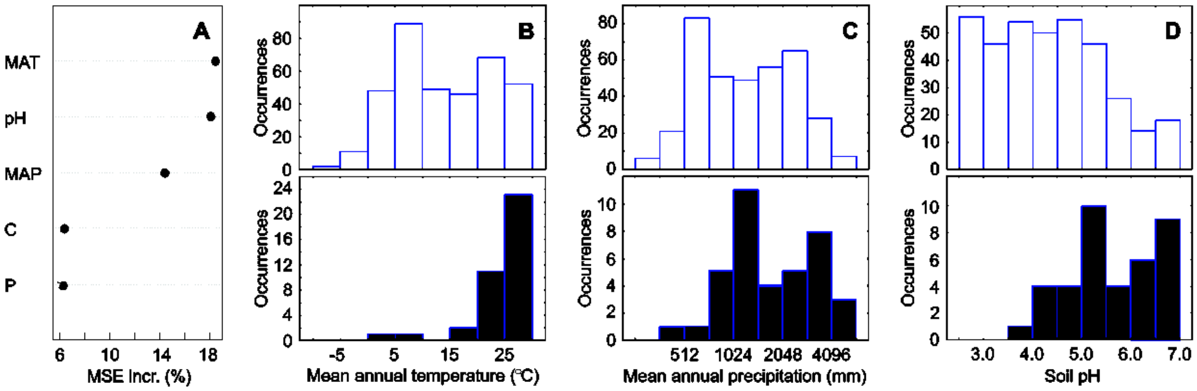

Figure. Niche analysis of the clade. A. Strongest predictors for the occurrence of clade members based on random forest analysis. B-D. Distribution of clade members (lower panels) in sites with (B) different mean annual temperature, (C) mean annual precipitation, and (D) average soil pH relative to all studied soils (upper panels).

Branch7

Previous names for the clade: -
Corresponding PlutoF compound cluster: -

Representative sequence: UDB014800 (habitat: site AV103; Colombia; 0.3919 S/ 72.0461 W; tropical lowland rain forest with no clearly dominant trees)
Contains other sequences: -

Phylogenetic position: a single divergent branch within Lecanoromycetes, Ascomycota

Ecological habitat: tropical lowland rain forests at extremely low pH
Distribution: almost exclusive to northern South America, except a single finding from India

Other habitats: -

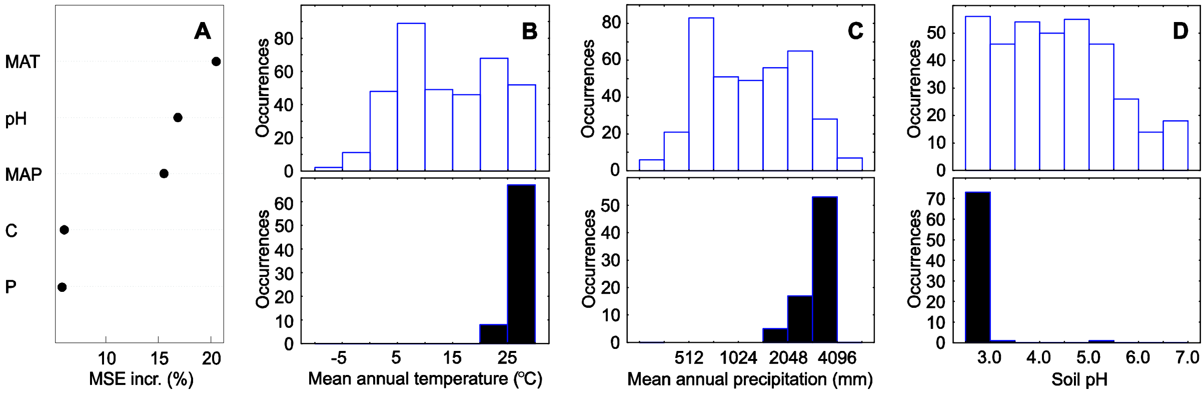

Figure. Niche analysis of the clade. A. Strongest predictors for the occurrence of clade members based on random forest analysis. B-D. Distribution of clade members (lower panels) in sites with (B) different mean annual temperature, (C) mean annual precipitation, and (D) average soil pH relative to all studied soils (upper panels).

Clade GS36

Previous names for the clade: -
Corresponding PlutoF compound cluster: -

Representative sequence: UDB014939 (habitat: site G2736; Papua New Guinea; 6.3755 S/ 145.2558 E; tropical montane rain forest dominated by *Castanopsis acuminatissima*)
Contains other sequences: UDB014955, KF567558

Phylogenetic position: a clade within Lecanoromycetes, Ascomycota

Ecological habitat: most common in various tropical biomes and Mediterranean habitats; very wide pH tolerance
Distribution: most common in Africa and Madagascar but present in all continents

Other habitats: temperate forest soil in NC, USA (KF567558)

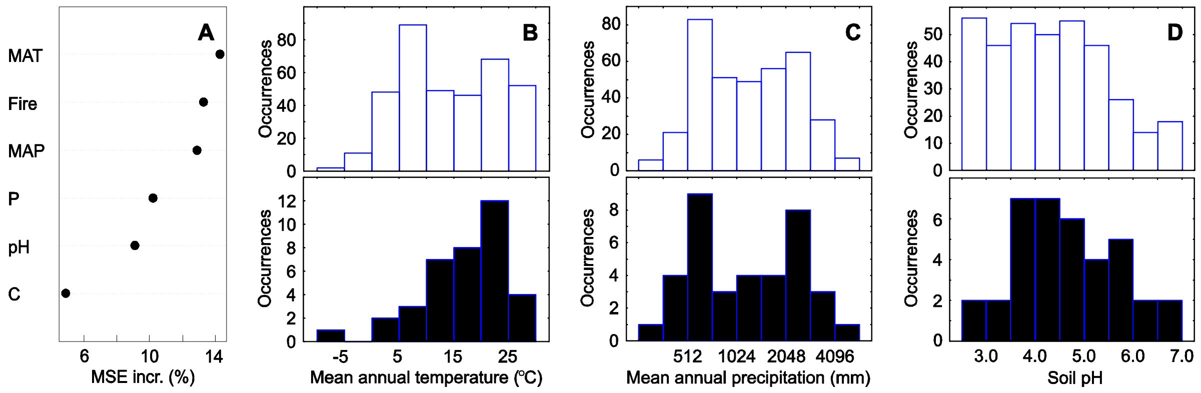

Figure. Niche analysis of the clade. A. Strongest predictors for the occurrence of clade members based on random forest analysis. B-D. Distribution of clade members (lower panels) in sites with (B) different mean annual temperature, (C) mean annual precipitation, and (D) average soil pH relative to all studied soils (upper panels).

Clade GS37

Previous names for the clade: -
Corresponding PlutoF compound cluster: -

Representative sequence: UDB014659 (habitat: site S123; Italy; 37.9017 N/ 14.0000 E; montane temperate deciduous forest dominated by *Fagus sylvatica*)
Contains other sequences: AB986436

Phylogenetic position: one of the early diverging lineages of Eurotiomycetes, Ascomycota

Ecological habitat: cool temperate climate, narrow preference for moderately acidic soils
Distribution: most common in southern South America but present in temperate Eurasia and tropical Africa

Other habitats: subtropical forest in FL, USA (AB986436)

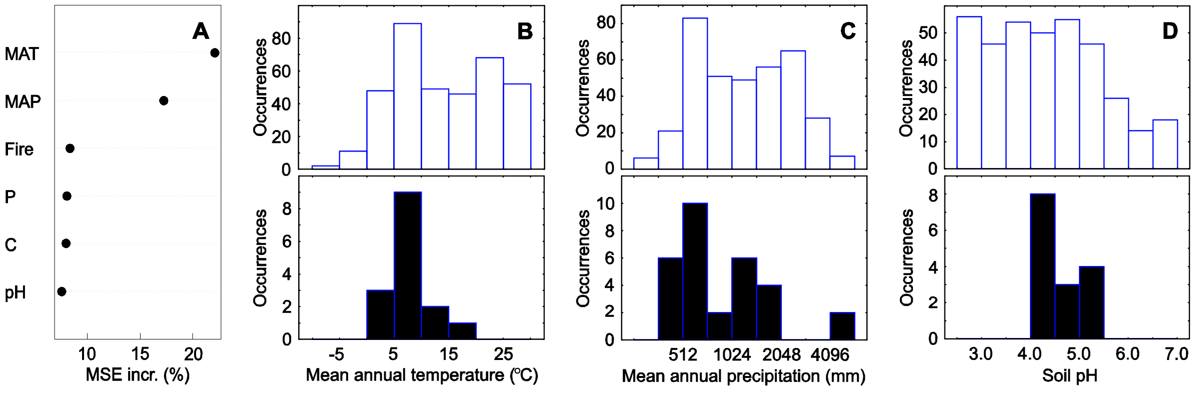

Figure. Niche analysis of the clade. A. Strongest predictors for the occurrence of clade members based on random forest analysis. B-D. Distribution of clade members (lower panels) in sites with (B) different mean annual temperature, (C) mean annual precipitation, and (D) average soil pH relative to all studied soils (upper panels).

Text S1.44
